# CHTOP regulates the NRF2/HO-1 axis to modulate chemoresistance in colorectal cancer

**DOI:** 10.1016/j.redox.2026.104353

**Published:** 2026-08-15

**Authors:** Jing Li, Xiaopeng Li, Chenye Zhao, Peiwen Wu, Weibin Hu, Xu Zhao, Yuan Ma, Zepeng Dong, Hang Yuan, Shihui Chen, Zilu Chen, Jing Lu, Wei Wang, Xuejun Sun, Qin Zhang, Mingchao Mu

**Affiliations:** a Department of Radiation Oncology, the First Affiliated Hospital of Xi'an Jiaotong University, Xi'an, China; b Department of General Surgery, the First Affiliated Hospital of Xi'an Jiaotong University, Xi'an, China; c Department of Dermatology, Northwest Hospital, the Second Affiliated Hospital of Xi'an Jiaotong University, Xi'an, China; d Center for Translational Medicine, the First Affiliated Hospital of Xi'an Jiaotong University, Xi'an, China

**Keywords:** CHTOP, NRF2, Oxidative stress, Chemoresistance, PSIP1

## Abstract

Chemotherapy resistance remains a significant challenge in colorectal cancer (CRC) treatment, with disrupted redox balance playing a central role. Here we identify CHTOP as a key regulator of oxidative stress and chemoresistance in CRC. Mechanistically, CHTOP promotes NRF2 transcriptional activity by recruiting the SENP3-containing 5FMC complex to deSUMOylate NRF2, thereby sustaining HO-1 expression and redox balance. Notably, CHTOP expression itself is tightly controlled by a feedback mechanism. The p52 isoform of PSIP1 inhibits CHTOP expression by interfering with HNRNPH1-mediated splicing, leading to CHTOP degradation via nonsense-mediated decay (NMD). Conversely, elevated oxidative stress stabilizes SENP3, which promotes deSUMOylation and degradation of p52, thereby relieving p52-mediated suppression of CHTOP expression. This establishes an oxidative stress-SENP3-p52-CHTOP feedback loop that fine-tunes CHTOP levels. In 5-FU-resistant CRC cells, CHTOP downregulation shifts cells into an elevated oxidative stress state, which correlates with reduced 5-FU sensitivity. Notably, either restoring CHTOP expression or further depleting CHTOP disrupts this redox balance and resensitizes resistant cells to 5-FU. These findings suggest that modulating CHTOP expression may offer a therapeutic strategy to overcome chemoresistance in CRC through redox regulation.

## 1 Introduction

Chemotherapy remains one of the main therapeutic strategies for colorectal cancer (CRC), with first-line regimens typically including fluoropyrimidines such as 5-fluorouracil (5-FU), often combined with oxaliplatin or irinotecan [1]. The development of chemoresistance, however, substantially limits clinical efficacy and remains a major obstacle to improving patient outcomes [2,3]. Understanding the molecular mechanisms underlying chemoresistance is therefore essential for developing targeted strategies to overcome therapeutic failure. Resistance involves diverse changes in signaling, metabolism, and stress responses, with dysregulated reactive oxygen species (ROS) and redox balance playing a central role in both chemotherapy-induced cytotoxicity and resistance [[4], [5], [6]].

ROS function as signaling molecules at physiological levels but cause DNA, protein, and lipid damage when in excess [7]. Many anticancer drugs, including cisplatin, doxorubicin, and 5-FU, exert part of their cytotoxicity by elevating intracellular ROS, which cancer cells can counteract by enhancing antioxidant defenses [[7], [8], [9]]. ROS are often elevated in chemoresistant cells, where they may promote resistance via pathways such as PI3K/AKT or DUOX2-mediated epithelial-mesenchymal transition [4,5]. To cope with ROS, cancer cells activate cytoprotective programs, most prominently the NF-E2-related factor 2 (NRF2) pathway [10]. NRF2 is a basic leucine zipper (bZIP) transcription factor that serves as the master regulator of the antioxidant response and contains multiple conserved functional domains (Neh1–Neh7) [11,12]. The KEAP1-NRF2 system operates as a thiol-based sensor-effector apparatus in which KEAP1 senses electrophilic and oxidative insults through its reactive cysteine residues, while NRF2 transduces these signals to activate cytoprotective gene expression [10,11]. Under basal conditions, NRF2 is kept low by KEAP1-CUL3 ubiquitination. Upon oxidative stress, NRF2 translocates to the nucleus and activates antioxidant genes, including HO-1, mitigating oxidative damage and maintaining redox homeostasis during chemotherapy [13,14].

Despite the well-established role of NRF2/HO-1 in oxidative stress adaptation, the upstream regulators of this pathway remain poorly defined. We focused on chromatin target of PRMT1 (CHTOP), a vertebrate-specific chromatin-associated protein that regulates transcription and RNA processing [15,16]. CHTOP expression is tightly controlled through an autoregulatory intron retention mechanism antagonized by heterogeneous nuclear ribonucleoprotein H1 (HNRNPH1) [17], ensuring precise cellular dosage. In addition, arginine-methylated CHTOP recruits the nuclear five friends of methylated CHTOP (5FMC) complex, which includes the SUMO-specific protease 3 (SENP3) [16]. Because SUMOylation can modulate NRF2 transcriptional activity [18,19], these findings raise the possibility that CHTOP, via SENP3, may influence NRF2-mediated redox homeostasis. However, whether CHTOP modulates the oxidative stress response and chemotherapeutic sensitivity in CRC remains unknown.

In this study, we identify CHTOP as a key regulator of oxidative stress and 5-FU response in CRC. Altering CHTOP expression perturbs the oxidative stress steady state that underlies acquired resistance, thereby restoring sensitivity to chemotherapy.

## 2 Results

### 2.1 CHTOP interacts with NRF2

**Fig. 1:**
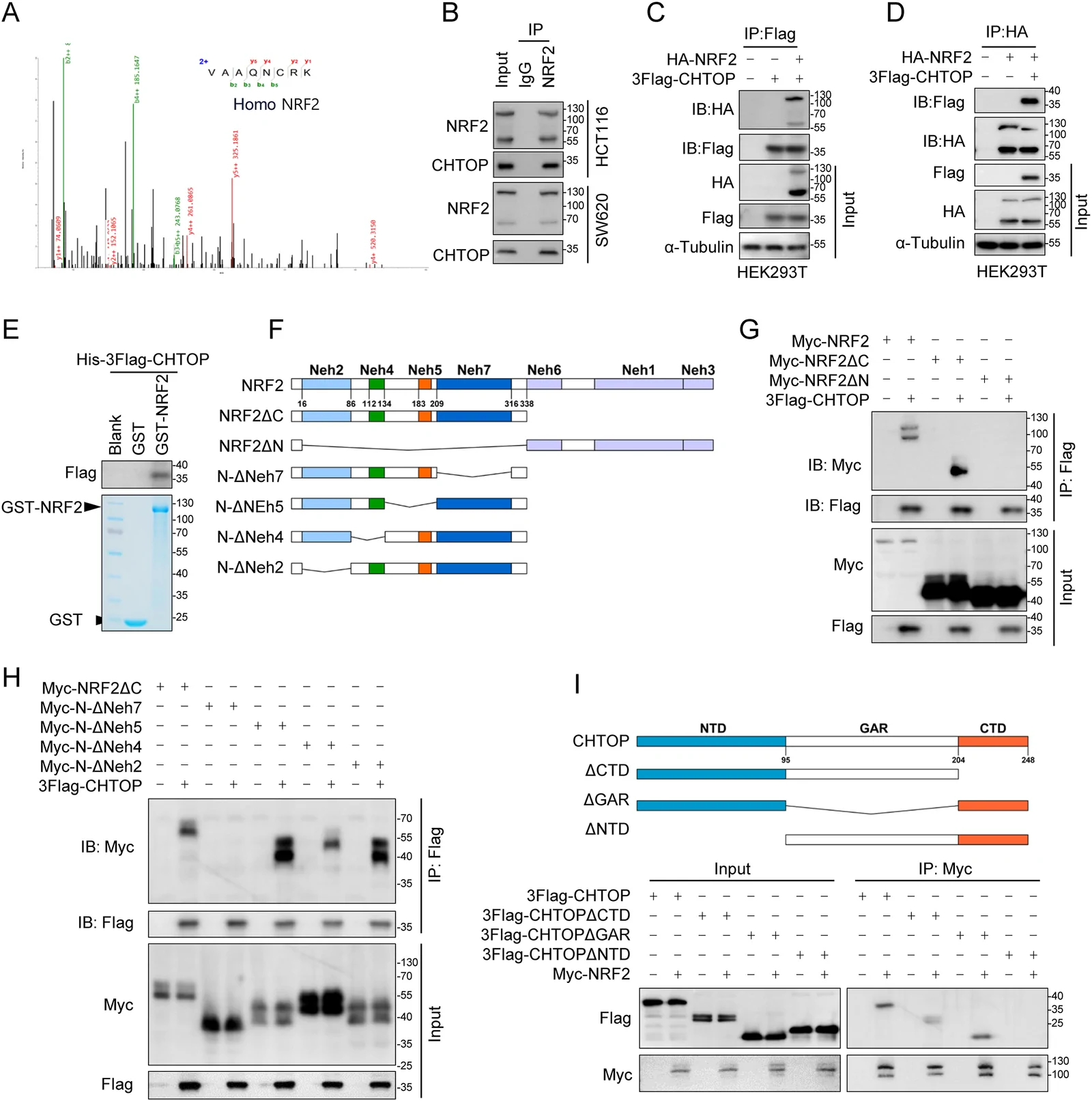
**CHTOP interacts with NRF2. A** Mass spectrometry spectrum of a peptide derived from human NRF2 protein. **B** Endogenous interaction between NRF2 and CHTOP in HCT116 and SW620 cells detected by immunoprecipitation with an anti-NRF2 antibody and immunoblotting with an anti-CHTOP antibody. **C** HEK293T cell lysates co-expressing HA-NRF2 and 3×Flag-CHTOP were subjected to immunoprecipitation with an anti-Flag antibody, followed by immunoblotting with an anti-HA antibody. **D** Reciprocal immunoprecipitation of HA-NRF2 from HEK293T cells co-expressing 3×Flag-CHTOP, followed by immunoblotting with an anti-Flag antibody. **E** GST pull-down assay demonstrating the direct interaction between NRF2 and CHTOP. **F** Schematic representation of full-length NRF2 and its truncation mutants. **G-H** HEK293T cell lysates expressing Myc-NRF2 or its truncation mutants together with 3×Flag-CHTOP were immunoprecipitated using an anti-Flag antibody and analyzed by immunoblotting with an anti-Myc antibody. **I** Schematic representation of full-length CHTOP and its truncation mutants (upper panel). HEK293T cells co-expressing Myc-NRF2 with 3×Flag-CHTOP or its truncation mutants were subjected to immunoprecipitation using an anti-Myc antibody and analyzed by immunoblotting with an anti-Flag antibody (lower panel).

To investigate the molecular mechanisms of CHTOP in CRC, we first sought to identify its interacting proteins. LC-MS/MS analysis of anti-Flag immunoprecipitates from HCT116 cells stably expressing 3×Flag-tagged CHTOP revealed NRF2 among the most prominent candidates (Fig. 1A). To confirm this interaction and enable reliable detection of NRF2 in subsequent experiments, we first validated the specificity of commercially available anti-NRF2 antibodies.

We characterized three commercially available anti-NRF2 antibodies by Western blot and immunofluorescence (IF), as NRF2 antibodies are known to exhibit variable specificity. In HCT116, SW480 and SW620 cell lysates, Abcam ab137550 detected a single band at approximately 110 kDa, whereas Santa Cruz sc-365949 and Proteintech 16396-1-AP both detected an additional lower band at approximately 68 kDa (Fig. S1A). To determine whether both bands represent NRF2-specific signals, we performed knockdown of NRF2 using two independent shRNAs in HCT116 cells. As shown in Fig. S1B, the intensities of both the 110 kDa and 68 kDa bands were markedly reduced in both cytoplasmic and nuclear fractions upon NRF2 knockdown. Consistently, IF staining showed a substantial decrease in NRF2 fluorescence intensity following knockdown with both shRNAs (Fig. S1C). These results confirm that both antibodies specifically recognize NRF2, and we therefore selected Santa Cruz sc-365949 and Proteintech 16396-1-AP for all subsequent experiments.

Having validated the specificity of NRF2 detection, we next sought to confirm the CHTOP-NRF2 interaction identified by LC-MS/MS. In both HCT116 and SW620 cells, endogenous CHTOP co-immunoprecipitated (Co-IP) with NRF2, as detected by both the 110 kDa and 68 kDa bands (Fig. 1B). Reciprocal Co-IP in HEK293T cells co-expressing HA-NRF2 and 3×Flag-CHTOP showed that HA-NRF2 was immunoprecipitated with anti-Flag antibody (Fig. 1C), and conversely, 3×Flag-CHTOP was immunoprecipitated with anti-HA antibody (Fig. 1D). Furthermore, an in vitro GST pull-down assay using purified proteins demonstrated a direct physical interaction between NRF2 and CHTOP (Fig. 1E).

To examine whether CHTOP and NRF2 colocalize in cells, we performed confocal microscopy in HCT116 and SW620 cells under basal conditions. In the absence of exogenous stimulation, NRF2 was predominantly cytoplasmic, with a relatively weak but detectable nuclear signal, while CHTOP was mainly nuclear; their colocalization was modest (Fig. S1D). Since NRF2 nuclear translocation can be induced by electrophilic agents, we treated cells with increasing concentrations of tert-Butylhydroquinone (tBHQ) (10-100 μM). While total NRF2 protein levels remained largely unchanged, its downstream target HO-1 showed a dose-dependent increase, confirming NRF2 activation (Fig. S1E). Based on these results, 50 μM tBHQ was selected for subsequent experiments. Under this condition, tBHQ treatment markedly induced NRF2 nuclear translocation and significantly enhanced its colocalization with CHTOP (Fig. S1F). Notably, Co-IP showed that tBHQ did not substantially increase the CHTOP-NRF2 binding affinity (Fig. S1G), suggesting that the enhanced colocalization reflects increased nuclear accumulation of NRF2, which bringing CHTOP and NRF2 into closer proximity, rather than increased binding affinity.

To map the regions mediating the CHTOP-NRF2 interaction, we generated a series of NRF2 truncation mutants (Fig. 1F). Co-IP showed that deletion of the Neh7 domain (ΔNeh7) abolished the interaction with CHTOP, whereas other truncation mutants retained binding (Fig. 1G and H). Conversely, deletion of the N-terminal domain (NTD) of CHTOP similarly disrupted its interaction with NRF2 (Fig. 1I), indicating that the Neh7 domain of NRF2 and the NTD of CHTOP are each required for their mutual binding.

To gain structural insight into this interaction, we performed AlphaFold-Multimer prediction for the CHTOP-NRF2 and CHTOP-Neh7 complexes. The models yielded low confidence scores (CHTOP-NRF2: pTM = 0.19, ipTM = 0.23; CHTOP-Neh7: pTM = 0.13, ipTM = 0.06) (Fig. S1H). As a control, we also predicted the known RXRα-NRF2 Neh7 interaction, which gave similarly low scores (RXRα-NRF2: pTM = 0.3, ipTM = 0.2; RXRα-Neh7: pTM = 0.44, ipTM = 0.4), consistent with the intrinsically disordered nature of the Neh7 domain. To biochemically validate the direct interaction, we performed an in vitro GST pull-down assay using purified GST-Neh7 and His-3×Flag-CHTOP proteins. The results showed that CHTOP was specifically pulled down by GST-Neh7, but not by GST alone (Fig. S1I), confirming a direct physical interaction between CHTOP and the Neh7 domain of NRF2.

### 2.2 CHTOP is required for NRF2 transcriptional activity

**Fig. 2:**
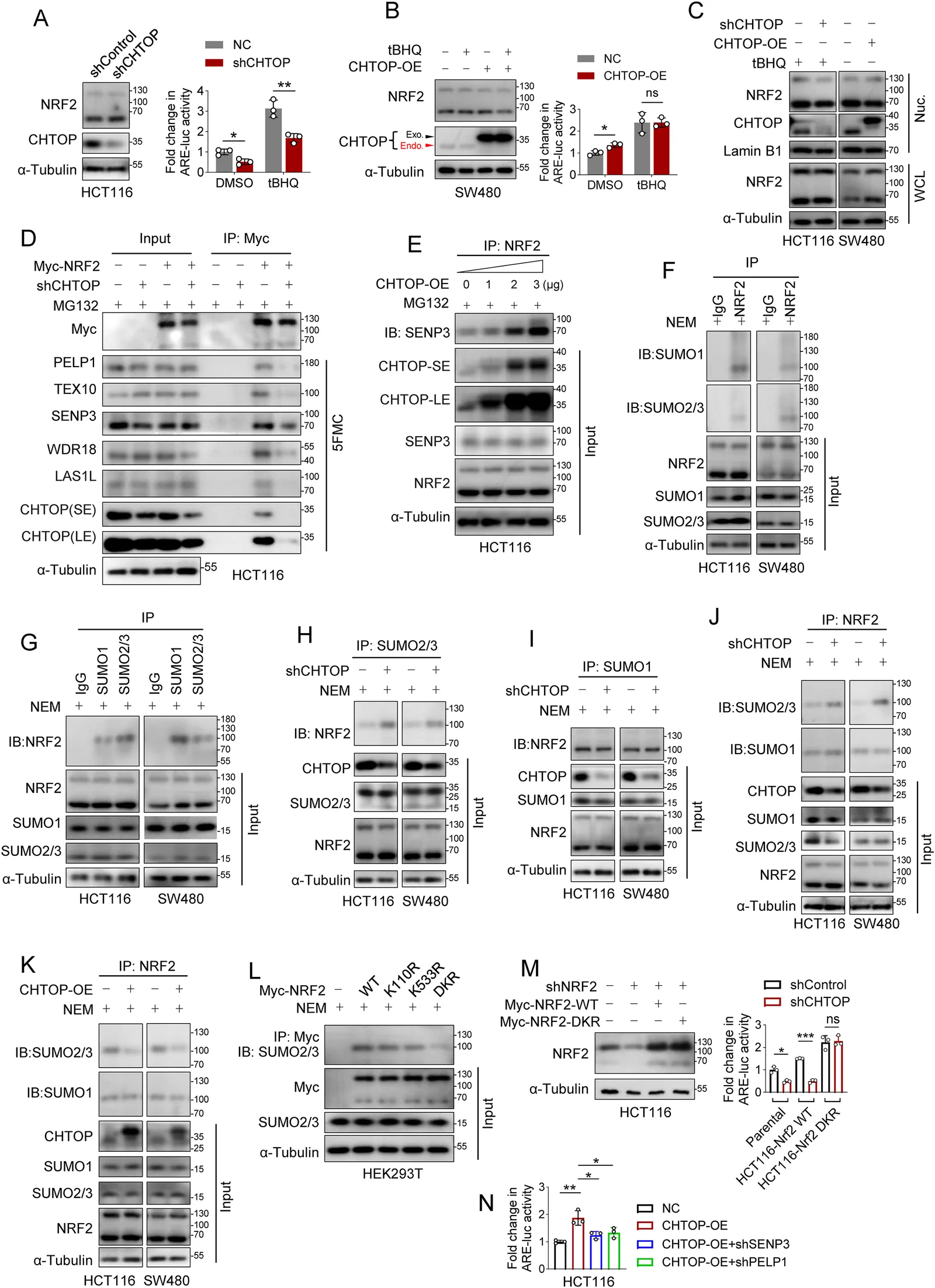
**CHTOP is required for NRF2 transcriptional activity. A** CHTOP knockdown did not alter NRF2 protein levels (left). Both basal and tBHQ-induced (50 μM, 24 h) ARE-luciferase activities were reduced upon CHTOP knockdown (right). **B** Neither CHTOP overexpression nor tBHQ treatment (50 μM, 24 h) affected NRF2 protein abundance (left). CHTOP overexpression enhanced basal ARE-luciferase activity but did not further increase tBHQ-induced ARE activation (right). **C** CHTOP knockdown or overexpression did not affect NRF2 protein levels in either nuclear fractions or whole-cell lysates. **D** Lysates from HCT116 cells expressing Myc-NRF2 with or without CHTOP knockdown were immunoprecipitated using an anti-Myc antibody and analyzed by immunoblotting with antibodies recognizing members of the 5FMC complex. **E** Lysates from HCT116 cells expressing increasing amounts of CHTOP were subjected to immunoprecipitation using an anti-NRF2 antibody, followed by immunoblotting with an anti-SENP3 antibody. **F** Endogenous NRF2 SUMOylation in HCT116 and SW480 cells was examined by immunoprecipitating NRF2 and immunoblotting with anti-SUMO1 or anti-SUMO2/3 antibodies. **G** Reciprocal immunoprecipitation with anti-SUMO1 or anti-SUMO2/3 antibodies followed by immunoblotting for NRF2. **H-I** Lysates from cells with or without CHTOP knockdown were immunoprecipitated using anti-SUMO2/3 (**H**) or anti-SUMO1 (**I**) antibodies and probed for NRF2. **J** NRF2 was immunoprecipitated from cells with or without CHTOP knockdown and analyzed for SUMO2/3 and SUMO1 conjugation. **K** NRF2 was immunoprecipitated from cells with or without CHTOP overexpression and examined for SUMO2/3 and SUMO1 modification. **L** Lysates from HEK293T cells expressing Myc-NRF2 or its SUMO-deficient DKR mutants (K110R, K533R) or single mutants (K110R or K533R) were subjected to anti-Myc immunoprecipitation and immunoblotted with anti-SUMO2/3 antibody. **M** Endogenous NRF2 was depleted, and shRNA-resistant Myc-NRF2-WT or the K110R/K533R mutant was re-expressed in HCT116 cells (left). CHTOP knockdown was then introduced into parental cells, WT-NRF2-reconstituted cells, and mutant-NRF2-reconstituted cells to assess its effects on ARE-luciferase activity (right). **N** ARE-luciferases activity in HCT116 cells overexpressing CHTOP alone or together with SENP3 or PELP1 knockdown. Exo.: Exogenous; Endo.: Endogenous. Data are expressed as mean ± SD, n = 3 biological replicates. *P < 0.05, **P < 0.01, ***P < 0.001.

To examine whether CHTOP affects NRF2 transcriptional activity, we knocked down CHTOP in HCT116 and SW480 cells using three independent shRNAs (shCHTOP#1-#3), among which shCHTOP#3 achieved the most efficient suppression (Fig. S2A) and was selected for subsequent experiments. Knockdown of CHTOP in HCT116 cells strongly suppressed both basal and tBHQ-induced NRF2 transcriptional activity, without affecting NRF2 total protein levels in whole-cell lysates (Fig. 2A). Conversely, overexpression of 3×Flag-CHTOP in SW480 cells enhanced basal NRF2 activity with little effect on tBHQ-induced activation (Fig. 2B). Consistently, CHTOP overexpression increased basal NRF2 activity with little effect on tBHQ-induced activation in HCT116 cells (Fig. S2B), while CHTOP knockdown markedly inhibited basal and tBHQ-induced NRF2 activity in SW480 cells (Fig. S2C). Notably, CHTOP knockdown in HCT116 cells and CHTOP overexpression in SW480 cells did not alter NRF2 protein levels in whole-cell lysates or nuclear fractions (Fig. 2C).

Previous studies indicate that SUMOylation plays a vital role in regulating NRF2 transcriptional activity [18,19], and CHTOP is known to recruit the nuclear 5FMC complex—including PELP1, SENP3, WDR18, TEX10, and LAS1L [16]. Based on these observations, we hypothesized that CHTOP may modulate NRF2 SUMOylation and thereby its transcriptional activity by recruiting SENP3.

In HCT116 cells, we confirmed that NRF2 co-immunoprecipitated with each 5FMC member, and this interaction was markedly disrupted upon CHTOP knockdown (Fig. 2D). Conversely, increasing exogenous 3×Flag-CHTOP led to a dose-dependent increase in the amount of SENP3 co-immunoprecipitated with NRF2 (Fig. 2E), demonstrating that CHTOP physically links SENP3 to NRF2.

We next assessed whether CHTOP-SENP3 binding influences NRF2 SUMOylation. Western blot analyses of immunoprecipitates revealed that NRF2 is conjugated with both SUMO1 and SUMO2/3 (∼100 kDa band; Fig. 2F and G). Notably, CHTOP knockdown selectively increased SUMO2/3- but not SUMO1-conjugated NRF2 in both anti-SUMO and anti-NRF2 immunoprecipitates (Fig. 2H–J), whereas CHTOP overexpression reduced SUMO2/3 conjugation without affecting SUMO1 modification (Fig. 2K). Furthermore, knockdown of SENP3, but not SENP1 or SENP2, attenuated the CHTOP-induced reduction of SUMO2/3-conjugated NRF2 in SW480 cells (Fig. S2D). Collectively, these results indicate that CHTOP bridges NRF2 and SENP3 to regulate the SUMO2/3 modification of NRF2.

To pinpoint the SUMO2/3 acceptor sites on NRF2, we used the online tool Joined Advanced Sumoylation Site and SIM Analyzer [20] and predicted 16 putative SUMO sites (Fig. S2E); however, mutation of any individual site did not significantly abolish SUMO2/3 conjugation of NRF2 (Fig. S2F). In contrast, simultaneous mutation of K110 and K533, which are located within the only two negatively charged amino acid-dependent SUMOylation motifs in NRF2, strongly diminished SUMO2/3 modification (Fig. 2L), indicating that these residues serve as the predominant SUMO2/3 acceptor sites.

To further determine the functional relevance of these modifications, endogenous NRF2 was depleted in HCT116 cells and rescued with shRNA-resistant wild-type or SUMO2/3-resistant (K110R/K533R) constructs. CHTOP knockdown suppressed the transcriptional activity of wild-type NRF2 but had minimal effect on the K110R/K533R mutant (Fig. 2M), demonstrating that CHTOP regulates NRF2 activity specifically through SUMO2/3 modification at these sites.

Finally, knockdown of PELP1, a core subunit essential for 5FMC stability [16], disrupted complex assembly and attenuated CHTOP-induced NRF2 activation, similar to the effect of SENP3 depletion in both HCT116 (Fig.S2G, Fig. 2N) and SW480 cells (Fig. S2H and I). Collectively, these findings establish that CHTOP bridges NRF2 to the SENP3-containing 5FMC complex to promote SENP3-dependent deSUMOylation of NRF2, thereby enhancing its transcriptional activity.

### 2.3 CHTOP maintains redox balance through the NRF2/HO-1 axis

**Fig. 3:**
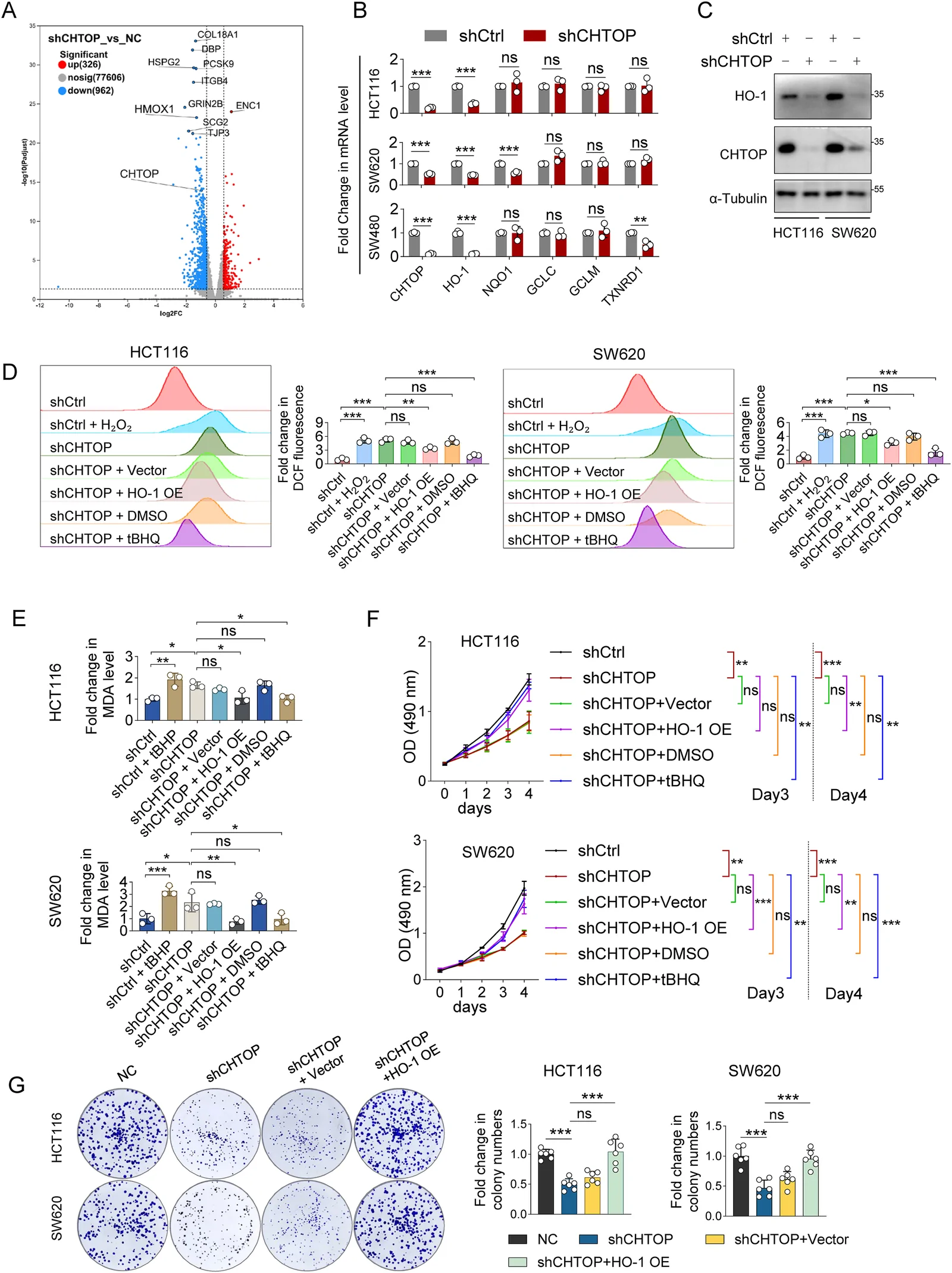
**CHTOP maintains redox balance through the NRF2/HO-1 axis. A** Volcano plot showing differentially expressed genes from RNA-seq analysis of HCT116 cells with CHTOP knockdown compared to control. **B** Relative mRNA level of HO-1, NQO1, GCLC, GCLM and TXNRD1 in HCT116, SW620 and SW480 cells following CHTOP knockdown. **C** Western blot analysis of HO-1 protein levels in HCT116 and SW620 cells following CHTOP knockdown. **D** Flow cytometry analysis of DCF fluorescence in HCT116 and SW620 cells with the indicated treatments. H_2_O_2_ (100 μM, 4 h) served as a positive control. **E** MDA levels in HCT116 and SW620 cells under the same experimental conditions, with tBHP (100 μM, 8 h) as a positive control. **F** MTT assay of HCT116 and SW620 cells with CHTOP knockdown, HO-1 overexpression, or tBHQ (50 μM, 24 h) treatment. **G** Colony formation assay of HCT116 and SW620 cells with the indicated treatments. Data are expressed as mean ± SD, n = 3 biological replicates in A, B, D, E and F, n = 6 biological replicates in G. *P < 0.05, **P < 0.01, ***P < 0.001.

Having established that CHTOP promotes NRF2 transcriptional activity, we next examined whether CHTOP is required for NRF2-dependent antioxidant gene expression and redox balance. Bulk RNA-seq analysis in HCT116 cells revealed that CHTOP knockdown selectively reduced HO-1 (HMOX1) mRNA levels, whereas other canonical NRF2 targets, including NQO1, GCLC, GCLM, and TXNRD1, were not significantly altered (Fig. 3A; Supplementary Data 1). To assess whether this selective regulation was cell line-specific, we also examined CHTOP knockdown in SW480 and SW620 cells. RT-qPCR analysis showed that HO-1 was the only NRF2 target gene consistently downregulated across all three cell lines, whereas NQO1 and TXNRD1 were reduced only in SW620 and SW480 cells, respectively (Fig. 3B). Given its conserved downregulation, we focused on HO-1 as the key functional mediator downstream of CHTOP. Consistently, CHTOP knockdown reduced HO-1 protein levels in both HCT116 and SW620 cells (Fig. 3C).

To evaluate whether CHTOP-modulated HO-1 expression affects cellular redox status, we measured oxidative stress levels using DCFH-DA fluorescence in HCT116 and SW620 cells. DCFH-DA is a cell-permeable probe that becomes fluorescent upon oxidation, serving as a surrogate marker of cellular oxidative stress. As shown in Fig. 3D, treatment with H_2_O_2_ (100 μM, 4 h), used as a positive control, markedly increased DCF fluorescence in both cell lines. CHTOP knockdown significantly elevated DCF fluorescence, indicating increased oxidative stress. Importantly, co-expression of HO-1 effectively reversed the CHTOP knockdown-induced increase in DCF fluorescence. Similarly, treatment with tBHQ (50 μM, 24 h) also reversed the effect of CHTOP knockdown, consistent with the restoration of HO-1 expression. To further assess oxidative damage, we measured malondialdehyde (MDA) levels, a stable end-product of lipid peroxidation, using tert-Butyl hydroperoxide (tBHP) (100 μM, 8 h) as a positive control. As expected, tBHP treatment markedly elevated MDA levels in both cell lines (Fig. 3E). CHTOP knockdown similarly increased MDA levels, which were reversed by HO-1 overexpression or tBHQ treatment (Fig. 3E).

To examine whether CHTOP-regulated HO-1 expression affects cell proliferation, we performed MTT assays in HCT116 and SW620 cells. In HCT116 cells, CHTOP knockdown significantly reduced cell viability at both day 3 and day 4. HO-1 overexpression did not rescue the growth defect at day 3 but almost completely restored viability at day 4, whereas tBHQ treatment (50 μM, 24 h) restored viability at both time points (Fig. 3F). In SW620 cells, CHTOP knockdown similarly reduced viability, and both HO-1 overexpression and tBHQ treatment (50 μM, 24 h) effectively reversed this effect at both time points (Fig. 3F). Colony formation assays confirmed that CHTOP knockdown decreased clonogenic capacity in both cell lines, and HO-1 overexpression largely restored this ability (Fig. 3G). These results indicate that CHTOP is required for cell proliferation largely through the NRF2/HO-1 axis.

### 2.4 The p52 isoform of PSIP1 facilitates CHTOP pre-mRNA degradation

Having established that CHTOP maintains redox balance through the NRF2/HO-1 axis, we next investigated how CHTOP expression itself is regulated. HNRNPH1 has been reported to bind to the intron-2 region of CHTOP pre-mRNA and promote its excision, which prevents intron-2 retention and subsequent degradation via nonsense-mediated decay (NMD) [17]. We therefore speculated that other HNRNPH1-interacting proteins may modulate the stability of CHTOP pre-mRNA.

**Fig. 4:**
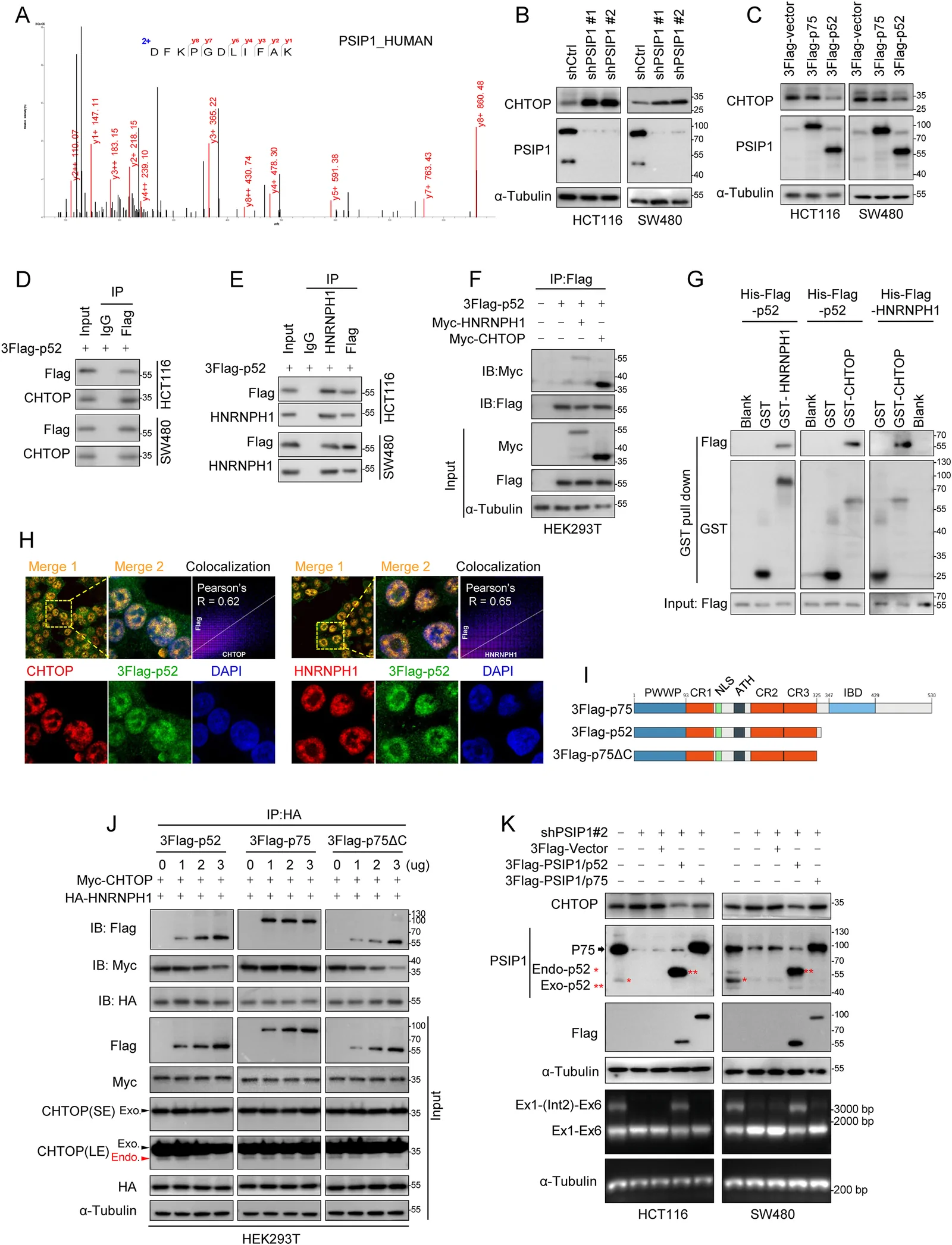
**The p52 isoform of PSIP1 facilitates CHTOP pre-mRNA degradation. A** Mass spectrometry spectrum of a peptide derived from PSIP1, which contains a common sequence shared by both p75 and p52 isoforms. **B** Protein level changes of CHTOP in HCT116 and SW480 cells following PSIP1 knockdown (knockdown of both p75 and p52). **C** Protein level changes of CHTOP in HCT116 and SW480 cells following overexpression of p75 or p52. **D** Lysates from cells expressing 3×Flag-p52 were subjected to immunoprecipitation using an anti-Flag antibody, followed by immunoblotting with an anti-CHTOP antibody. **E** Lysates from cells expressing 3×Flag-p52 were subjected to immunoprecipitation using an anti-HNRNPH1 antibody, followed by immunoblotting with an anti-Flag antibody, or using an anti-Flag antibody followed by immunoblotting with an anti-HNRNPH1 antibody. **F** Lysates from HEK293T cells co-expressing 3×Flag-p52, Myc-HNRNPH1, or Myc-CHTOP were subjected to immunoprecipitation using an anti-Flag antibody, followed by immunoblotting with an anti-Myc antibody. **G** GST pull-down assay showing the direct interaction between p52, HNRNPH1, and CHTOP. **H** Confocal microscopy images showing the co-localization of 3×Flag-p52 with CHTOP or HNRNPH1. **I** Schematic representation of 3×Flag-p75, 3×Flag-p52, and 3×Flag-p75ΔC. **J** Lysates from HEK293T cells co-expressing Myc-CHTOP, HA-HNRNPH1 with 3×Flag-p52, 3×Flag-p75, or 3×Flag-p75ΔC were subjected to immunoprecipitation using an anti-HA antibody, followed by immunoblotting with anti-Flag and anti-Myc antibodies. **K** Western blot showing changes in CHTOP protein levels, and agarose gel electrophoresis analysis of intron-2-containing pre-mRNA levels of CHTOP, following PSIP1 knockdown (both p75 and p52), or simultaneous PSIP1 knockdown with shRNA-resistant exogenous overexpression of 3×Flag-p75 or 3×Flag-p52. On the agarose gel, the band around 3000 bp corresponds to the intron-2-containing pre-mRNA of CHTOP, while the band around 2000 bp represents CHTOP mRNA.

We identified HNRNPH1-interacting proteins by LC-MS/MS analysis of anti-Myc immunoprecipitates from HCT116 cells stably expressing Myc-tagged HNRNPH1. Among the most prominent candidates were PSIP1, a known mRNA splicing factor interactor, and CHTOP (Fig. 4A, Fig. S3A and B). PSIP1 has two isoforms: the well-characterized p75 and the less-studied p52. HNRNPH1 co-precipitated peptides (DFKPGDLIFAK, aa4-aa14) that are shared by both isoforms. Interestingly, knockdown of PSIP1 (targeting both p52 and p75) led to a marked increase in CHTOP protein (Fig. 4B), whereas overexpression of p52, but not p75, resulted in a pronounced decrease in CHTOP levels (Fig. 4C). Analysis of the Gene Expression Omnibus (GEO) database (GSE99699) also showed that knockdown of p75 alone, without affecting p52, had no impact on CHTOP mRNA expression (Fig. S3C). Collectively, these results indicate that CHTOP expression is specifically regulated by the p52 isoform of PSIP1.

In both HCT116 and SW480 cells, endogenous CHTOP was co-immunoprecipitated with exogenous 3×Flag-p52 (Fig. 4D). Similarly, reciprocal co-immunoprecipitation confirmed the interaction between endogenous HNRNPH1 and exogenous 3×Flag-p52 (Fig. 4E). Moreover, in HEK293T cells co-expressing 3×Flag-p52 with Myc-HNRNPH1 or Myc-CHTOP, both Myc-HNRNPH1 and Myc-CHTOP were readily detected in the anti-Flag immunoprecipitates (Fig. 4F). GST pull-down assays further confirmed a direct physical interaction between purified CHTOP, HNRNPH1 and p52 (Fig. 4G). Immunofluorescence analysis revealed that both endogenous CHTOP and HNRNPH1 co-localized with exogenous 3×Flag-p52 in the nucleus (Fig. 4H).

We next asked whether p52 modulates the interaction between HNRNPH1 and CHTOP. In HEK293T cells, increasing amounts of exogenous p52 led to a dose-dependent reduction in CHTOP co-immunoprecipitated with HNRNPH1(Fig. 4I and J). Interestingly, a mutant form of p75 lacking the C-terminal region, which contains the IBD domain, exhibited a similar effect, while full-length p75 had no significant impact (Fig. 4I and J). Consistently, PSIP1 knockdown (p75 + p52) enhanced excision of intron-2 and increased CHTOP protein levels, whereas re-expression of p52, but not p75, promoted intron-2 retention and decreased CHTOP protein levels (Fig. 4K). These findings indicate that p52 interferes with HNRNPH1-mediated excision of CHTOP intron-2, thereby promoting pre-mRNA degradation and reducing CHTOP expression.

### 2.5 Oxidative stress–SENP3 axis drives p52 deSUMOylation and degradation upon CHTOP depletion

Previous studies reported that both PSIP1 isoforms, p75 and p52, are SUMO-3 targets, and SUMOylation of p75 modulates its half-life [21]; however, whether p52 stability is similarly affected remains unknown. Moreover, oxidative stress is known to stabilize SENP3 [22], a key regulator of SUMO2/3 modification [23,24]. We therefore speculated that oxidative stress may regulate p52 stability via SENP3.

**Fig. 5:**
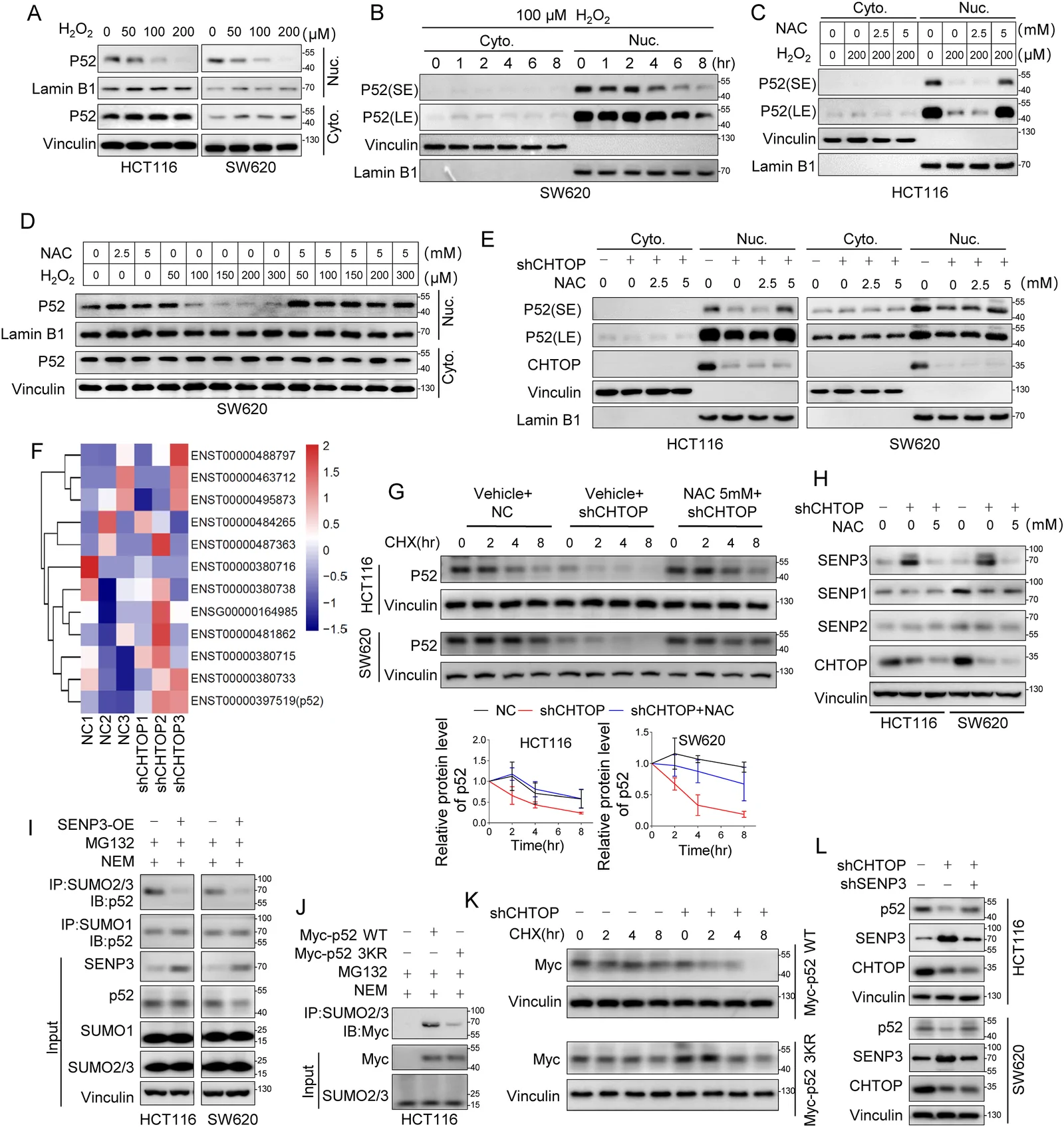
**Oxidative stress stabilizes SENP3 to complete a negative feedback loop via p52 degradation. A** Western blot analysis of nuclear and cytoplasmic p52 protein levels in HCT116 and SW620 cells treated with increasing concentrations of H_2_O_2_ (0-200 μM) for 4 h. **B** Time-course analysis of nuclear and cytoplasmic p52 protein levels in SW620 cells treated with 100 μM H_2_O_2_ for the indicated times. **C** Western blot analysis of nuclear and cytoplasmic p52 protein levels in HCT116 cells treated with H_2_O_2_ (200 μM, 4 h) alone or in combination with 2.5-5 mM NAC. **D** Western blot analysis of nuclear and cytoplasmic p52 protein levels in SW620 cells treated with H_2_O_2_ (50-300 μM, 4 h) alone or in combination with 5 mM NAC. **E** Western blot analysis of nuclear and cytoplasmic p52 protein levels in cells with CHTOP knockdown alone or combined with 2.5-5 mM NAC. **F** Expression changes of the different PSIP1 transcripts following CHTOP knockdown in HCT116 cells. **G** CHX chase assays showing p52 protein levels following CHX treatment for the indicated times in cells with CHTOP knockdown alone or combined with NAC. **H** Alterations in SENP3, SENP1, and SENP2 protein levels in cells following CHTOP knockdown alone or combined with NAC. **I** Lysates from cells with or without SENP3 knockdown were subjected to immunoprecipitation using anti-SUMO2/3 or anti-SUMO1 antibodies, followed by immunoblotting with an anti-PSIP1/p52 antibody. **J** Lysates from HCT116 cells expressing Myc-p52 WT or Myc-p52-3KR (K75R/K250R/K254R) were subjected to immunoprecipitation using anti-SUMO2/3 antibody, followed by immunoblotting with an anti-Myc antibody. **K** CHX chase assays showing Myc-tagged p52 protein levels following CHX treatment for the indicated times in HCT116 cells expressing Myc-p52-WT or Myc-p52-3KR, with or without CHTOP knockdown. **L** Alterations in p52 protein levels in cells with CHTOP knockdown alone or combined with SENP3 knockdown.

Treatment of HCT116 and SW620 cells with increasing concentrations of H_2_O_2_ (0-200 μM) for 4 h induced a dose-dependent decrease in p52 protein levels, predominantly in the nuclear fraction, with no significant change in the cytoplasmic fraction (Fig. 5A). Time-course analysis in SW620 cells treated with 100 μM H_2_O_2_ revealed that nuclear p52 began to decline at approximately 4 h, while cytoplasmic p52 showed no notable change (Fig. 5B). The H_2_O_2_-induced reduction of nuclear p52 was abolished by 5 mM NAC in both HCT116 (Fig. 5C) and SW620 cells (Fig. 5D), confirming that ROS promotes p52 degradation in the nucleus.

CHTOP knockdown significantly reduced p52 protein levels in the nuclear fraction, whereas cytoplasmic p52 remained largely unchanged; this effect was reversed by 5 mM NAC treatment (Fig. 5E), indicating that CHTOP regulates p52 stability in an oxidative stress-dependent manner, predominantly in the nucleus. Bulk RNA-sequencing revealed that p52 mRNA levels were not reduced upon CHTOP knockdown (Fig. 5F), indicating that this regulation occurs at the post-translational level. Consistently, cycloheximide (CHX) chase assays demonstrated that CHTOP knockdown accelerated p52 protein degradation, which was reversed by NAC treatment (Fig. 5G).

These results indicate that elevated oxidative stress caused by CHTOP depletion contributes to p52 destabilization. We next examined ROS-sensitive regulators and found that CHTOP knockdown markedly increased SENP3 protein levels, whereas SENP1 and SENP2 remained unchanged; this effect was reversed by NAC treatment (Fig. 5H). SENP3 specifically reduced SUMO2/3 conjugation of p52 without affecting SUMO1 (Fig. 5I). Exogenous Myc-p52 carrying the K75R/K250R/K254R (3 KR) mutations [21] exhibited reduced SUMO2/3 modification (Fig. 5J). Upon CHTOP knockdown, this mutant degraded more slowly than wild-type Myc-p52 (Fig. 5K). Furthermore, SENP3 knockdown attenuated the reduction of p52 induced by CHTOP depletion (Fig. 5L).

Taken together, these findings suggest that CHTOP depletion increases oxidative stress, leading to SENP3 stabilization. SENP3 then promotes deSUMOylation of p52, leading to enhanced p52 degradation. This process reveals an oxidative stress-SENP3-SUMO2/3-p52 regulatory axis downstream of CHTOP.

### 2.6 CHTOP deficiency contributes to chemoresistance in CRC

Given our findings that CHTOP regulates cellular redox homeostasis through the NRF2/HO-1 axis, we hypothesized that alterations in CHTOP expression may influence chemotherapy response. To test this, we focused on three clinically relevant chemotherapeutic agents used in CRC treatment: 5-FU, oxaliplatin, and irinotecan. Analysis of GEO datasets revealed that 5-FU treatment significantly downregulated CHTOP and upregulated HO-1 in HCT8 cells (Fig. S4A). Consistently, 5-FU-resistant HCT116 cells appeared to show lower CHTOP and higher HO-1 levels compared with parental cells (Fig. S4B). In contrast, oxaliplatin treatment increased HO-1 expression without affecting CHTOP, and oxaliplatin-resistant cells showed no changes in either CHTOP or HO-1 (Fig. S4C and D). Similarly, irinotecan treatment reduced CHTOP and elevated HO-1, whereas irinotecan-resistant cells maintained comparable CHTOP and HO-1 levels to their parental counterparts (Fig. S4E and F). Notably, both 5-FU treatment and 5-FU-resistant cells consistently displayed a reduction in CHTOP expression along with an increase in HO-1, suggesting a perturbation in oxidative stress regulation. Based on these findings, we chose to further investigate 5-FU as a model to explore whether CHTOP deficiency contributes to the development of chemoresistance in CRC.

**Fig. 6:**
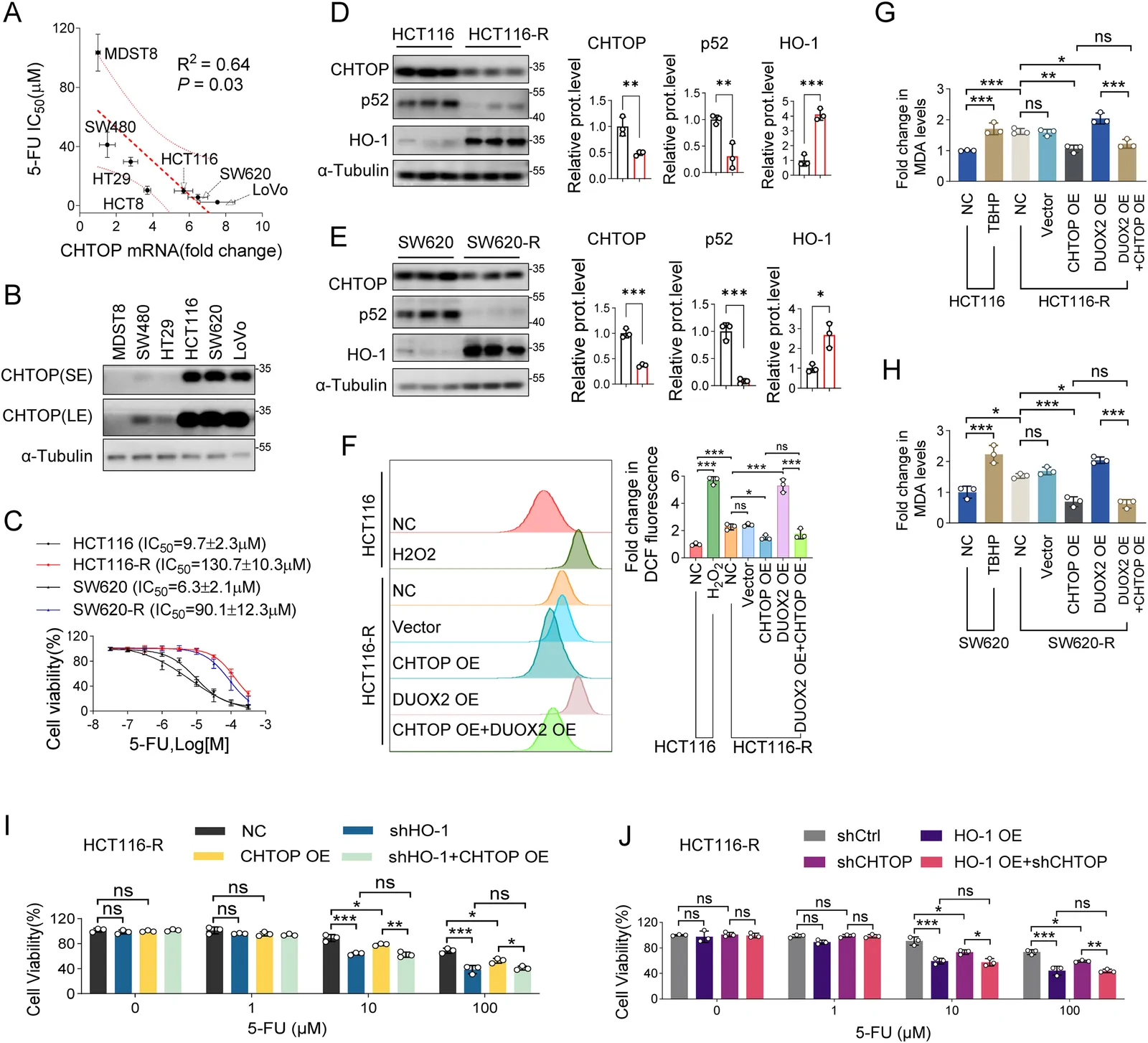
**CHTOP deficiency contributes to chemoresistance in CRC. A** Correlation between CHTOP mRNA levels and 5-FU IC50 values in a panel of CRC cell lines. **B** CHTOP protein levels in a panel of CRC cell lines. **C** Dose-response curve and IC50 values of 5-FU in HCT116, SW620, and their derived 5-FU-resistant cell lines (HCT116-R and SW620-R). **D-E** Western blot analysis and quantification of CHTOP, p52, and HO-1 protein levels in HCT116-R (**D**) and SW620-R (**E**) cells relative to their respective parental controls. **F.** Flow cytometry analysis of DCF fluorescence in HCT116 and HCT116-R cells expressing the indicated constructs. H_2_O_2_ (100 μM, 4 h) served as a positive control. **G.** MDA levels in HCT116 and HCT116-R cells expressing the indicated constructs. TBHP (100 μM, 8 h) served as a positive control. **H.** MDA levels in SW620 and SW620-R cells expressing the indicated constructs. **I.** MTT assay of HCT116-R cells treated with increasing concentrations of 5-FU (0, 1, 10, 100 μM) following HO-1 knockdown, CHTOP overexpression, or both. **J**. MTT assay of HCT116-R cells treated with increasing concentrations of 5-FU (0, 1, 10, 100 μM) following HO-1 overexpression, CHTOP knockdown, or both. Data are expressed as mean ± SD, n = 3 biological replicates. *P < 0.05, **P < 0.01, ***P < 0.001.

In our initial observations, we found a strong inverse correlation (R^2^ = 0.64) between the mRNA levels of CHTOP and the half-maximal inhibitory concentration (IC50) of 5-FU across a panel of CRC cell lines (Fig. 6A). Immunoblotting analysis revealed that cell lines with lower sensitivity to 5-FU (MDST8, SW480, and HT29) had significantly reduced CHTOP protein levels compared to the more 5-FU-sensitive cell lines (HCT116, SW620, and LoVo) (Fig. 6B).

Next, we generated two acquired 5-FU-resistant CRC cell lines, HCT116-R and SW620-R, by gradually exposing HCT116 and SW620 cells to increasing concentrations of 5-FU over a period of approximately 12 months. As a result, the IC50 values of 5-FU resistance increased from 9.7 ± 2.3 μM (HCT116) and 6.3 ± 2.1 μM (SW620) to 130.7 ± 10.3 μM (HCT116-R) and 90.1 ± 12.3 μM (SW620-R), respectively (Fig. 6C).

Consistent with the data from the GEO database, both the mRNA (Fig. S4G and H) and protein levels (Fig. 6D and E) of CHTOP were significantly decreased in HCT116-R and SW620-R cells. Additionally, the mRNA and protein levels of HO-1 were clearly elevated (Figs. S4G, H, Fig. 6D, E). While the mRNA levels of p52 remained unchanged (Fig. S4G and H), its protein levels were markedly reduced (Fig. 6D and E). Furthermore, CHTOP overexpression increased both p52 and HO-1 protein levels in HCT116-R and SW620-R cells (Fig. S4I).

Flow cytometry analysis revealed that DCF fluorescence was significantly elevated in HCT116-R cells compared to their parental counterparts, confirming a higher oxidative stress state in the drug-resistant cells (Fig. 6F). Overexpression of CHTOP in HCT116-R cells reduced DCF fluorescence, whereas overexpression of DUOX2, a ROS-generating enzyme [5], alone had the opposite effect; notably, co-expression of CHTOP with DUOX2 reversed the DUOX2-induced elevation, bringing DCF fluorescence to levels comparable to CHTOP overexpression alone (Fig. 6F). These results indicate that CHTOP overexpression counteracts DUOX2-driven oxidative stress in resistant cells, consistent with its role in regulating the NRF2/HO-1 axis. This oxidative stress regulation pattern was consistent in SW620-R cells (Fig. S4J). MDA assays corroborated these findings. MDA levels were significantly elevated in HCT116-R cells compared to their parental counterparts. In HCT116-R cells, CHTOP overexpression reduced MDA levels, whereas DUOX2 overexpression led to an increase; co-expression of CHTOP with DUOX2 reversed the DUOX2-induced elevation (Fig. 6G). Similar results were observed in SW620-R cells (Fig. 6H).

We next examined whether CHTOP-mediated redox regulation affects 5-FU sensitivity in resistant cells. HCT116-R cells were treated with increasing concentrations of 5-FU (0, 1, 10, 100 μM) following HO-1 knockdown, CHTOP overexpression, or both (Fig. 6I). At 10 μM and 100 μM 5-FU, HO-1 knockdown markedly reduced cell viability. CHTOP overexpression also decreased viability, albeit to a lesser extent. Notably, combining HO-1 knockdown with CHTOP overexpression did not further reduce viability beyond HO-1 knockdown alone, suggesting that the sensitizing effect of CHTOP overexpression depends on HO-1 and is mediated through the NRF2/HO-1 axis (Fig. 6I). Similar results were observed in SW620-R cells (Fig. S4K). In contrast, CHTOP knockdown in parental HCT116 and SW620 cells did not confer 5-FU resistance (Fig. S4L and M), despite reducing basal cell viability.

Interestingly, HO-1 overexpression also markedly reduced cell viability at 10 μM and 100 μM 5-FU, while CHTOP knockdown produced a similar but weaker effect (Fig. 6J). Combining HO-1 overexpression with CHTOP knockdown yielded no additional reduction beyond HO-1 overexpression alone, indicating that CHTOP knockdown also sensitizes resistant cells through the NRF2/HO-1 axis (Fig. 6J). This finding was also observed in SW620-R cells (Fig. S4N). Collectively, these results demonstrate that in resistant cells, both upregulation and downregulation of CHTOP disrupt the 5-FU-resistant phenotype.

### 2.7 CHTOP modulation disrupts chemoresistance and sensitizes 5-FU-resistant colorectal tumors in vivo

**Fig. 7:**
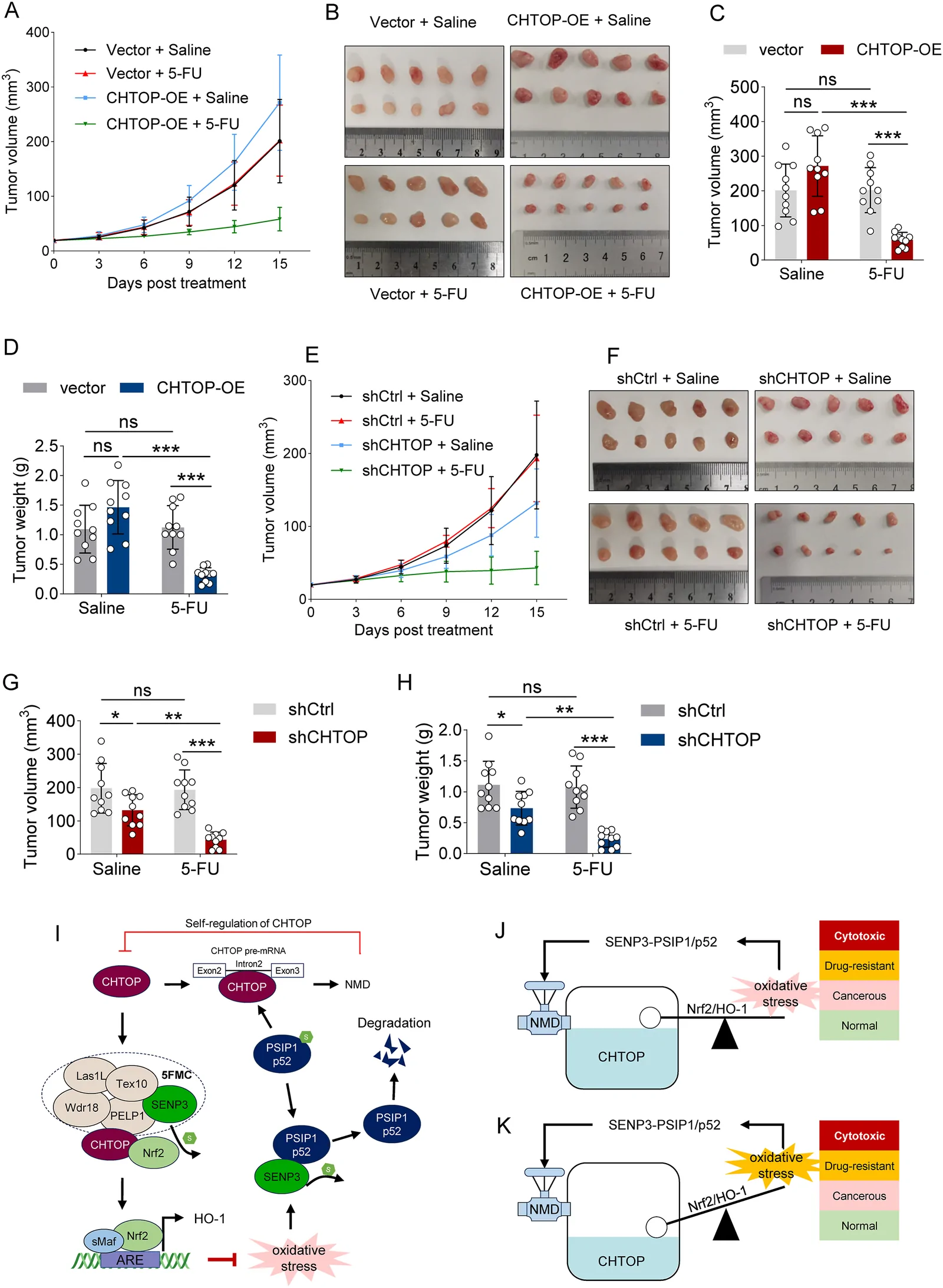
**CHTOP modulation disrupts chemoresistance and sensitizes 5-FU-resistant colorectal tumors in vivo. A-D** HCT116-R cells with or without CHTOP overexpression were subcutaneously implanted to form tumor xenografts, followed by treatment with either saline or 5-FU. (**A)** Tumor growth curve showing volume progression. (**B)** Images of subcutaneous tumors at the endpoint. (**C)** Statistical analysis of tumor volume at the end of treatment. (**D)** Statistical analysis of tumor weight at the end of treatment. **E-H** HCT116-R cells with or without CHTOP knockdown were subcutaneously implanted to form tumor xenografts, followed by treatment with either saline or 5-FU. (**E)** Tumor growth curve showing volume progression. (**F)** Images of subcutaneous tumors at the endpoint. (**G)** Statistical analysis of tumor volume at the end of treatment. (**H)** Statistical analysis of tumor weight at the end of treatment. **I** Mechanism diagram of CHTOP-mediated oxidative stress regulation. **J-K** Water tank model illustrating CHTOP regulation of oxidative stress. **(J)** When CHTOP levels increase (“water in the tank” rises), CHTOP promotes its own pre-mRNA degradation via NMD (“overflow”). Through the NRF2/HO-1 axis, oxidative stress decreases, leading to reduced SENP3 levels and increased SUMOylation of PSIP1/p52. p52 accumulation further promotes NMD-mediated CHTOP pre-RNA degradation (“overflow valve” opens), resulting in CHTOP reduction. (**K)** When CHTOP levels decrease (“water in the tank” drops), NRF2/HO-1 is suppressed, and oxidative stress increases. SENP3 accumulates, promoting p52 deSUMOylation and degradation. Reduced p52 levels relieve the suppression on CHTOP (“overflow valve” closes). However, this closure is insufficient to fully restore CHTOP expression. Thus, moderate downregulation of CHTOP may help maintain oxidative stress within a pro-resistance range. Data are expressed as mean ± SD, n = 10 biological replicates. *P < 0.05, **P < 0.01, ***P < 0.001.

We next investigated whether CHTOP overexpression could restore 5-FU sensitivity in HCT116-R tumors in vivo using a subcutaneous xenograft model (Fig. 7A–D). Two-way ANOVA revealed a significant interaction between CHTOP overexpression and 5-FU treatment on both tumor volume and tumor weight (P < 0.0001 for both). While CHTOP overexpression alone did not affect tumor growth in the absence of 5-FU, it markedly enhanced tumor sensitivity to 5-FU, as evidenced by significantly reduced tumor volume and weight in CHTOP-overexpressing tumors compared to vector controls under 5-FU treatment (Fig. 7C and D).

We then examined whether CHTOP knockdown similarly sensitizes HCT116-R tumors to 5-FU (Fig. 7E–H). Two-way ANOVA revealed a significant interaction between CHTOP knockdown and 5-FU treatment on both tumor volume and tumor weight (P < 0.05). CHTOP knockdown alone significantly reduced tumor growth under saline conditions, and this effect was further enhanced by 5-FU treatment, resulting in the most pronounced tumor regression in the combination group (Fig. 7G and H). These results suggest that modulation of CHTOP expression, through either overexpression or knockdown, disrupts the chemoresistant phenotype and restores 5-FU sensitivity in colorectal tumors in vivo.

## 3 Discussion

ROS levels are tightly regulated in cells, but in cancer, disrupted ROS homeostasis affects tumor behavior and therapy responses in a context-dependent manner [[25], [26], [27], [28]]. Many chemotherapeutic agents further increase ROS to suppress cancer growth; however, tumor cells can adapt by reinforcing antioxidant defenses and maintaining ROS at elevated but sub-suppressive levels, which is closely associated with drug resistance [4,5,27,29,30]. Consistently, our study shows that CHTOP downregulation shifts cells into an elevated oxidative stress state, correlating with reduced 5-FU sensitivity, which may result from cells surviving the initial oxidative stress increase adapting to maintain this elevated oxidative stress.

NRF2 contains multiple conserved functional domains (Neh1–Neh7) [11,12]. Among these, the Neh7 domain has been reported to interact with RXRα and suppress NRF2 transcriptional activity [12]. In contrast to this repressive function, our study reveals that CHTOP acts through the same Neh7 domain to promote NRF2 transcriptional activity. Interestingly, analysis of additional canonical NRF2 target genes indicated that CHTOP does not uniformly regulate the NRF2 transcriptional program. Instead, among the NRF2 target genes examined in CRC cells, HO-1 showed the most consistent response to CHTOP modulation, suggesting a preferential effect of CHTOP on the NRF2/HO-1 axis in this context. We delineate a molecular mechanism by which CHTOP bridges NRF2 to the SENP3-containing 5FMC complex, promoting SUMO2/3 deconjugation of NRF2 at K110 and K533 and enhancing its transcriptional activity, thereby sustaining HO-1 expression and maintaining basal oxidative stress levels. The p52 isoform of PSIP1 modulates CHTOP pre-mRNA stability by interfering with HNRNPH1-mediated intron-2 excision, leading to NMD of CHTOP transcripts. When CHTOP expression declines, oxidative stress levels rise and stabilize SENP3; stabilized SENP3 promotes deSUMOylation and degradation of p52, thereby alleviating p52-mediated inhibition of HNRNPH1-dependent CHTOP splicing and helping maintain CHTOP expression. This establishes an oxidative stress -SENP3-p52-CHTOP negative feedback loop. Thus, CHTOP not only sustains HO-1 expression and basal oxidative stress levels through NRF2, but also engages a self-stabilizing mechanism that protects its own expression.

Notably, HO-1 is widely recognized for its cytoprotective functions, and its upregulation is frequently associated with chemoresistance in cancer cells, whereas HO-1 downregulation has been linked to increased drug sensitivity [31,32]. However, emerging evidence indicates that the relationship between HO-1 levels and cell fate is not strictly linear [33,34]. In certain contexts, excessive HO-1 expression can be detrimental, primarily through iron accumulation and Fenton chemistry-driven ROS production, which promote lipid peroxidation and ferroptosis [[35], [36], [37]]. Our observation that both CHTOP overexpression and knockdown disrupt the oxidative stress steady state and resensitize resistant cells to 5-FU supports the notion that CHTOP maintains HO-1 expression within a functionally optimal range, such that perturbation in either direction yields convergent phenotypic outcomes.

We use “water tank model” to illustrate how CHTOP regulate oxidative stress. In tumor cells (Fig. 7J), CHTOP expression is regulated by an “NMD overflow valve” that is controlled by the oxidative stress-dependent p52 “gate”. When CHTOP expression increases (water in the tank rises), the overflow valve is activated, and the reduction in oxidative stress induced by elevated CHTOP expression leads to the upregulation of p52. This upregulation of p52 further opens the NMD overflow valve, promoting the degradation of CHTOP via NMD (overflow). As a result, CHTOP levels decrease. Thus, the upregulation of CHTOP is tightly controlled. When CHTOP expression is downregulated (water in the tank declines), oxidative stress increases. Oxidative stress -induced SENP3 accumulation, along with p52 deSUMOylation and degradation, may partially close the NMD overflow valve. However, this process is insufficient to fully restore CHTOP expression. Consequently, persistent downregulation of CHTOP leads to sustained oxidative stress elevation, which may contribute to chemoresistance by promoting a high-oxidative stress state. In cells treated with 5-FU, when CHTOP is excessively downregulated, oxidative stress levels increase to a level that induces cell death. However, when CHTOP is moderately downregulated, oxidative stress levels reach a threshold that may promote chemoresistance, allowing surviving cells to adapt and establish a drug-resistant steady state.

This model provides a coherent explanation for the seemingly paradoxical observations: both CHTOP overexpression and knockdown can resensitize 5-FU-resistant cells to 5-FU. In 5-FU-resistant cells, where CHTOP expression is low, oxidative stress is maintained within a narrow but tolerable elevated range. Both decreasing oxidative stress levels (via CHTOP overexpression) and pushing oxidative stress beyond the tolerable threshold (via CHTOP depletion) disrupt this redox steady state, thereby re-sensitizing resistant cells to 5-FU.

SUMO1 modification has been reported to enhance NRF2 nuclear localization and transcriptional activity, thereby reducing ROS levels in hepatocytes and beta cells [38,39], whereas SUMO2/3 modification is generally associated with decreased NRF2 nuclear levels and reduced transcriptional activity [18,19]. Consistently, our study demonstrates that CHTOP-mediated deconjugation of SUMO2/3 from NRF2 is essential for maintaining its transcriptional activity. In this context, CHTOP is critical for SENP3 to regulate NRF2, as loss of CHTOP disrupts the SENP3-NRF2 interaction, providing new insight into how SENP3 modulates oxidative stress [40,41].

The study also has some limitations. Although we observed that CHTOP overexpression can reduce tumor growth in vivo, consistent with previous reports that CHTOP reduced the percentage of cells in the G2/M phase and increased cell death in HeLa cells [42], the precise mechanisms behind this effect in CRC cells are still not fully clear. Additionally, while CHTOP modulation affects oxidative stress levels in vitro, the relationship between CHTOP and oxidative stress regulation within the tumor microenvironment in vivo has not been directly assessed. More detailed studies on oxidative stress dynamics in vivo would help clarify whether the therapeutic effects of CHTOP are mainly driven by changes in oxidative stress levels. Lastly, although our preclinical data is promising, further validation using patient-derived models or primary tumor samples is needed to better understand the clinical relevance of CHTOP modulation in overcoming 5-FU resistance in CRC.

In summary, our study identifies CHTOP as a key modulator of oxidative stress in CRC and chemoresistance. By regulating NRF2 activity and HO-1 expression, CHTOP maintains oxidative stress within a tolerable range and thereby sustains the chemoresistant phenotype. Perturbing CHTOP expression—either by overexpression or depletion—disrupts this oxidative stress steady state, thereby breaking the redox equilibrium that supports resistance. These findings highlight CHTOP as a potential therapeutic target for overcoming chemoresistance in CRC, and suggest that disrupting its regulatory function may offer a strategy to re-sensitize resistant cells through redox-based interventions.

## 4 Materials and methods

### 4.1 Reagents

MG132, cycloheximide (CHX), N-acetylcysteine (NAC), tert-butyl hydroperoxide (tBHP) and tert-butylhydroquinone (tBHQ) were purchased from Selleck (Houston, TX). N-ethylmaleimide (NEM) was obtained from Macklin (Shanghai, China). 5-Fluorouracil (5-FU) was purchased from Solarbio (Beijing, China). Hydrogen peroxide (H_2_O_2_) was obtained from Sigma-Aldrich (Darmstadt, Germany).

### 4.2 Cell cultures

HCT116, SW620, LoVo, HCT8, SW480, and HEK293T cell lines were purchased from Hysigen Bioscience (Suzhou, China). The MDST8 cell line was obtained from Sigma-Aldrich (Darmstadt, Germany), and the HT29 cell line was purchased from Procell Life Science & Technology Co., Ltd. (Wuhan, China). HCT116, SW480, MDST8, HT29, and HEK293T cells were cultured in Dulbecco's modified Eagle's medium (DMEM; Gibco, Grand Island, NY) supplemented with 10% fetal bovine serum (FBS; Gibco, Grand Island, NY). SW620, LoVo, and HCT8 cells were cultured in Roswell Park Memorial Institute medium (RPMI-1640; Gibco, Grand Island, NY) supplemented with 10% FBS. All cells were maintained at 37 °C in a humidified incubator with 5% CO_2_. All cell lines were recently authenticated by short tandem repeat (STR) profiling and tested negative for mycoplasma contamination.

### 4.3 Transfection of plasmid and lentiviral vectors

Lentiviral vectors pCMV-CHTOP, -NRF2, -HO-1, -PSIP1/p52, -PSIP1/p75, and -DUOX2 (GeneChem, Shanghai, China) were used to overexpress the respective genes. Plasmid expression constructs pCMV-NRF2 and its truncation mutants, as well as pCMV-CHTOP and its truncation mutants, were prepared by Fitgene Biotech (Guangzhou, China). Lentiviral phU6-EGFP-shRNA vectors (GeneChem, Shanghai, China) were used to silence CHTOP, HO-1, SENP1, SENP2, SENP3, PELP1, PSIP1, NRF2 and DUOX2. The plasmids pT7-PSIP1/p52, pT7-HNRNPH1, and pT7-CHTOP (GeneChem, Shanghai, China) were used to express the respective genes in *E. coli*. All transfections and viral infections were performed according to the manufacturers’ instructions. The target sequences of the shRNAs and the sequences of the shRNA-resistant constructs are provided in Supplementary Data 2.

### 4.4 Cell viability assay

Cell viability was assessed using the MTT assay (3-(4,5-dimethylthiazol-2-yl)-2,5-diphenyltetrazolium bromide (Sigma-Aldrich, Darmstadt, Germany). Cells were plated in 96-well plates, and at the designated time points, 10 μL of MTT solution (5 mg/mL) was added to each well containing 100 μL of medium. After a 4-h incubation, the supernatant was discarded and 150 μL of dimethyl sulfoxide (DMSO) was added to dissolve the formazan crystals. Absorbance was recorded at 490 nm with a reference wavelength of 630 nm.

### 4.5 RNA-sequencing

RNA-sequencing of HCT116 cells with or without CHTOP knockdown was performed on NovaSeq X Plus by Majorbio Pharmaceutical Technology (Shanghai, China). The data were analyzed by Majorbio Cloud online tools (https://cloud.majorbio.com/page/tools/). The results, including the top 50 ranked by *P*-value, are summarized in Supplementary Data 1.

### 4.6 Western blot analysis

Cells were lysed and subjected to SDS-PAGE after heat denaturation. Nuclear and cytoplasmic fractions were prepared using a Nuclear Extraction Kit (Abcam, ab113474, Cambridge, UK) according to the manufacturer's instructions. Proteins were transferred onto PVDF membranes (Millipore, Burlington, MA, USA) and blocked with 5% non-fat milk in TBST. Membranes were incubated with primary antibodies overnight at 4 °C, followed by HRP-conjugated secondary antibodies for 1 h at room temperature. Signals were detected using an ECL substrate (Abbkine Scientific, Wuhan, China). Antibody details are listed in Supplementary Data 3.

### 4.7 RT-qPCR and PCR analysis

Total RNA was extracted using the RNA Easy Fast Tissue/Cell Kit (TIANGEN, Beijing, China), and cDNA was synthesized with a reverse transcription kit (Takara, Tokyo, Japan). RT-qPCR was performed using the 2× RealStar Fast SYBR qPCR Mix (GenStar, Suzhou, China), and each reaction was conducted in triplicate and the relative gene expression levels were calculated using the 2^−ΔΔCt^ method with α-tubulin mRNA as the endogenous control. For conventional PCR, amplification was carried out using the 2× UltraTaq PCR StarMix (GenStar, Suzhou, China), and PCR products were resolved on 1% agarose gels (Beyotime, Shanghai, China) and visualized under UV light. All procedures were performed according to the manufacturers’ instructions. Primer sequences are provided in Supplementary Data 4.

### 4.8 Confocal microscopy

Cells were fixed with 4% paraformaldehyde for 10 min and permeabilized with 0.2% Triton X-100 (Solarbio, Beijing, China) for 10 min. After blocking with 1% BSA for 30 min at room temperature, the cells were incubated with primary antibodies overnight at 4 °C. The samples were then washed three times with PBS and incubated with secondary antibodies for 1 h at room temperature. Nuclei were counterstained with DAPI (Solarbio, Beijing, China) for 5 min. Images were acquired using a confocal fluorescence microscope (Leica Microsystems, Heidelberg, Germany). All confocal images were analyzed and quantified using the Colocalization Finder plugin (Version 1.9) in ImageJ [43]. Antibody details are in Supplementary Data 3.

### 4.9 Immunoprecipitation (IP) assay

Cell lysates were prepared using IP lysis buffer (Beyotime, Shanghai, China). Immunoprecipitation was performed using an IP/Co-IP Kit (Thermo Fisher Scientific, Waltham, MA). Briefly, lysates were incubated with 5 μg of primary antibody at 4 °C overnight. Magnetic beads were then added and incubated for 15 min, followed by three washes. Bound proteins were eluted using a low-pH elution buffer and neutralized with neutralization buffer. The eluates were subsequently analyzed by western blotting or subjected to mass spectrometry using a Thermo Scientific Q Exactive mass spectrometer (Fitgene Biotech, Guangzhou, China). When performing an IP assay to determine the level of SUMOylation, 20 mM NEM, an inhibitor of SUMO-specific proteases (SENPs), was added to the cell lysates. Antibody details are in Supplementary Data 3.

### 4.10 GST pull-down assay

His-Flag-p52, -CHTOP and -HNRNPH1 were expressed in *E. coli* BL21 (DE3) cells and purified using the His-tag Protein Purification Kit (Beyotime, Shanghai, China). Briefly, cells were harvested after induction, sonicated, and centrifuged to collect the supernatant. The supernatant was loaded onto a resin column, washed, and His-tagged proteins were eluted. Protein purity was confirmed by SDS-PAGE and Western blotting. GST, GST-CHTOP, GST-NRF2, GST-Neh7 and GST-HNRNPH1 fusion proteins were expressed in *E. coli* BL21(DE3) cells, and the bacterial lysates containing these GST-fusion proteins were incubated with glutathione sepharose 4B resin (Cytiva, Uppsala, Sweden) for affinity binding. After washing, the beads with immobilized GST-fusion proteins were incubated with the purified His-tagged protein for 2 h at 4 °C in an in vitro binding buffer [44]. Following incubation, the beads were washed three times and then eluted with sample buffer. The bound and input proteins were subjected to analysis via SDS-PAGE and Western blotting. Antibody details are in Supplementary Data 3.

### 4.11 Colony formation assay

For colony formation, 800 HCT116 cells or 1000 SW620 cells were plated into six-well plates and cultured for 10 or 14 days, respectively. At the end of the incubation period, colonies containing more than 50 cells were fixed, stained, and manually counted.

### 4.12 Malondialdehyde (MDA) assay

MDA levels were measured using a Lipid Peroxidation MDA Assay Kit (Beyotime, Shanghai, China) according to the manufacturer's protocol. Briefly, cells were lysed and centrifuged, and the resulting supernatants were incubated with the MDA working reagent at 100 °C for 15 min to form the MDA-TBA adduct. After cooling to room temperature and a second centrifugation, 200 μL of the clarified supernatant was transferred to a 96-well plate, and absorbance was measured at 532 nm. MDA concentrations were calculated from a standard curve and normalized to protein content.

### 4.13 Cellular oxidative stress assay

Intracellular oxidative stress levels were assessed using a DCFH-DA probe (Beyotime, Shanghai, China) following the manufacturer's instructions. Cells were collected and incubated with the DCFH-DA probe for 10 min at 37 °C in 5% CO_2_. After two washes with cold PBS, fluorescence intensity was analyzed by flow cytometry as a surrogate marker of cellular oxidative stress.

### 4.14 Luciferase reporter assay

The ARE-Luc Reporter Plasmid and pRL-TK Plasmids were obtained from GeneChem (Shanghai, China). 1 × 10^4^ cells were seeded into each well of a 96-well plate. Transfection was carried out by introducing 100 ng of firefly luciferase plasmid and 10 ng of pRL-TK plasmid using Lipo3000 (Thermo Fisher Scientific, Waltham, MA) as the transfection reagent. After 48 h of transfection, luminescence was measured using the Dual-Luciferase Reporter Gene Assay Kit II (Beyotime, Shanghai, China) with the Varioskan Flash system (Thermo Fisher Scientific, Waltham, MA).

### 4.15 Xenograft tumor models

The Ethics Committee of the Biomedical Ethics Committee of Health Science Center of Xi'an Jiaotong University approved the experiment. Sample sizes of 10 per group, with an equal distribution of males and females, were selected based on common practices in the field for similar phenotypic analyses. All mice were purchased from Shanghai SLAC Laboratory Animal Co., Ltd. (Shanghai, China) and housed in the animal facility of Xi'an Jiaotong University under specific-pathogen-free (SPF) conditions. 5 × 10^5^ cells suspended in 50 μL of PBS were subcutaneously injected into the right dorsal flanks of 8-week-old BALB/c nude mice. Treatment began when tumor volumes reached approximately 20 mm^3^. Tumor-bearing mice were randomly allocated to either the saline or 5-FU treatment group using a computer-generated random number sequence. Mice in the 5-FU group were treated with 5-FU via intraperitoneal injection at a dose of 20 mg/kg every 2 days. Mice in the saline group received an equal volume of saline via intraperitoneal injection every 2 days. The investigator assessing tumor outcomes was blinded to the treatment group allocation to minimize bias in data analysis. Tumor size was measured every three days using calipers, and tumor volume was calculated as ½ (L × W^2^). Fifteen days post-treatment, mice were euthanized by CO_2_ inhalation followed by cervical dislocation.

### 4.16 Statistical analysis

Data are expressed as mean ± SD. For comparisons between two groups, Student's t-test was used. For comparisons among more than two groups, one-way analysis of variance (ANOVA) was performed, followed by Tukey's post hoc test for pairwise comparisons. For comparisons among groups with two independent variables, two-way ANOVA was used, with Tukey's post hoc test for multiple comparisons between all groups. For comparison with GEO data, analysis was performed using the online tool GEO2R. A *P*-value of less than 0.05 was considered statistically significant.

## CRediT authorship contribution statement

**Jing Li:** Conceptualization, Funding acquisition, Methodology, Validation, Writing – original draft, Writing – review & editing. **Xiaopeng Li:** Conceptualization, Methodology, Project administration, Validation, Writing – original draft, Writing – review & editing. **Chenye Zhao:** Conceptualization, Methodology, Validation, Writing – original draft, Writing – review & editing. **Peiwen Wu:** Project administration. **Weibin Hu:** Project administration. **Xu Zhao:** Data curation, Formal analysis, Software. **Yuan Ma:** Data curation, Formal analysis. **Zepeng Dong:** Data curation, Formal analysis, Software. **Hang Yuan:** Project administration. **Shihui Chen:** Project administration. **Zilu Chen:** Data curation, Formal analysis, Software. **Jing Lu:** Conceptualization, Project administration, Supervision. **Wei Wang:** Conceptualization, Project administration, Supervision. **Xuejun Sun:** Conceptualization, Project administration, Supervision. **Qin Zhang:** Conceptualization, Investigation, Methodology, Project administration, Resources, Supervision, Validation, Writing – original draft, Writing – review & editing. **Mingchao Mu:** Conceptualization, Formal analysis, Funding acquisition, Methodology, Project administration, Resources, Supervision, Validation, Writing – original draft, Writing – review & editing.

## Ethics declaration

This study was conducted in accordance with the ARRIVE (Animal Research: Reporting of In Vivo Experiments) guidelines. This study was approved by the Biomedical Ethics Committee of Health Science Center of Xi'an Jiaotong University. (Approval No. XJTUAE2024-744; No. XJTUAE2025-2231)

## Data availability

RNA-seq data have been deposited in the GEO database under accession number GSE313017. Additional data related to this study are available upon request from the corresponding author.

## Declaration of competing interests

The authors declare the following financial interests/personal relationships which may be considered as potential competing interests: Jing Li reports financial support was provided by the National Natural Science Foundation of China. Jing Li reports financial support was provided by the Natural Science Basic Research Program of Shaanxi, China. Mingchao Mu reports financial support was provided by the Natural Science Basic Research Program of Shaanxi, China. Mingchao Mu reports financial support was provided by The First Affiliated Hospital of Xi'an Jiaotong University. If there are other authors, they declare that they have no known competing financial interests or personal relationships that could have appeared to influence the work reported in this paper.
