# Supplementary material for: CHTOP regulates the NRF2/HO-1 axis to modulate chemoresistance in colorectal cancer
Source: Redox Biol. 2026 Aug 15;96:104353. doi: 10.1016/j.redox.2026.104353 (PMC13521099; doi:10.1016/j.redox.2026.104353)
Supplement: Supplementary file 1 [file mmc1.pdf]

Supplementary data 1. Genes regulated by CHTOP knockdown (top 50)

| Gene id | Gene name | Log2FC(shCHTOP/NC) | Pvalue | Padjust | Regulate |
|---|---|---|---|---|---|
| ENSG00000182871 | COL18A1 | -1.340485237 | 6.22E-38 | 9.15E-34 | down |
| ENSG00000105516 | DBP | -1.552190117 | 1.77E-36 | 1.30E-32 | down |
| ENSG00000142798 | HSPG2 | -1.468958731 | 5.00E-34 | 2.45E-30 | down |
| ENSG00000169174 | PCSK9 | -1.306646839 | 8.44E-34 | 3.11E-30 | down |
| ENSG00000132470 | ITGB4 | -1.484206071 | 6.00E-32 | 1.77E-28 | down |
| ENSG00000273079 | GRIN2B | -2.077755888 | 1.18E-28 | 2.89E-25 | down |
| ENSG00000171617 | ENC1 | 1.106798387 | 4.98E-28 | 1.05E-24 | up |
| ENSG00000100292 | HMOX1 | -1.275006153 | 3.22E-27 | 5.93E-24 | down |
| ENSG00000171951 | SCG2 | -1.815162727 | 1.93E-25 | 3.15E-22 | down |
| ENSG00000105289 | TJP3 | -1.537401294 | 4.40E-25 | 6.48E-22 | down |
| ENSG00000179094 | PER1 | -1.025509747 | 1.46E-24 | 1.95E-21 | down |
| ENSG00000185033 | SEMA4B | -1.304015153 | 2.45E-24 | 2.87E-21 | down |
| ENSG00000103888 | CEMIP | -2.505853221 | 2.54E-24 | 2.87E-21 | down |
| ENSG00000074181 | NOTCH3 | -1.127558673 | 1.71E-23 | 1.80E-20 | down |
| ENSG00000137440 | FGFBP1 | -1.990540044 | 3.69E-23 | 3.62E-20 | down |
| ENSG00000172819 | RARG | -0.958469485 | 8.55E-22 | 7.86E-19 | down |
| ENSG00000105376 | ICAM5 | -1.359844703 | 1.48E-21 | 1.28E-18 | down |
| ENSG00000146242 | TPBG | -1.298186663 | 1.43E-20 | 1.17E-17 | down |
| ENSG00000163435 | ELF3 | -1.227572823 | 2.24E-20 | 1.73E-17 | down |
| ENSG00000196422 | PPP1R26 | -1.043924177 | 3.06E-20 | 2.21E-17 | down |
| ENSG00000234745 | HLA-B | -0.999238286 | 3.16E-20 | 2.21E-17 | down |
| ENSG00000174938 | SEZ6L2 | -1.144557065 | 3.49E-20 | 2.33E-17 | down |
| ENSG00000184254 | ALDH1A3 | -1.079657069 | 4.74E-20 | 2.97E-17 | down |
| ENSG00000052344 | PRSS8 | -1.264406977 | 4.84E-20 | 2.97E-17 | down |
| ENSG00000189143 | CLDN4 | -1.159609335 | 5.82E-20 | 3.43E-17 | down |
| ENSG00000142173 | COL6A2 | -1.071769202 | 1.25E-19 | 7.09E-17 | down |
| ENSG00000164292 | RHOBTB3 | 1.135127314 | 1.73E-19 | 9.44E-17 | up |
| ENSG00000117984 | CTSD | -0.934395415 | 2.90E-19 | 1.52E-16 | down |
| ENSG00000149948 | HMGA2 | 0.71838155 | 3.71E-19 | 1.88E-16 | up |
| ENSG00000089351 | GRAMD1A | -1.009140072 | 5.88E-19 | 2.89E-16 | down |
| ENSG00000128342 | LIF | -0.905367904 | 9.63E-19 | 4.57E-16 | down |
| ENSG00000141738 | GRB7 | -1.035393189 | 1.18E-18 | 5.42E-16 | down |
| ENSG00000183248 | PRR36 | -1.350762289 | 1.43E-18 | 6.38E-16 | down |
| ENSG00000142156 | COL6A1 | -0.96982544 | 2.09E-18 | 9.07E-16 | down |
| ENSG00000154945 | ANKRD40 | 0.81291329 | 3.08E-18 | 1.30E-15 | up |
| ENSG00000129824 | RPS4Y1 | 1.271580612 | 5.21E-18 | 2.12E-15 | up |
| ENSG00000171401 | KRT13 | -2.8701709 | 5.34E-18 | 2.12E-15 | down |
| ENSG00000126368 | NR1D1 | -1.084220717 | 7.45E-18 | 2.89E-15 | down |
| ENSG00000006534 | ALDH3B1 | -1.339794585 | 1.48E-17 | 5.60E-15 | down |
| ENSG00000077150 | NFKB2 | -0.934016371 | 1.84E-17 | 6.78E-15 | down |
| ENSG00000204525 | HLA-C | -0.83874613 | 1.99E-17 | 7.11E-15 | down |
| ENSG00000005884 | ITGA3 | -0.798446657 | 2.03E-17 | 7.11E-15 | down |
| ENSG00000160679 | CHTOP | -0.942803464 | 3.44E-17 | 1.18E-14 | down |
| ENSG00000170412 | GPRC5C | -1.331708098 | 3.94E-17 | 1.32E-14 | down |
| ENSG00000174791 | RIN1 | -1.012988363 | 4.31E-17 | 1.41E-14 | down |
| ENSG00000140961 | OSGIN1 | -1.285448657 | 5.07E-17 | 1.62E-14 | down |
| ENSG00000261150 | EPPK1 | -1.079774573 | 1.09E-16 | 3.40E-14 | down |
| ENSG00000111319 | SCNN1A | -0.8440786 | 1.26E-16 | 3.86E-14 | down |
| ENSG00000104881 | PPP1R13L | -1.114040803 | 1.41E-16 | 4.24E-14 | down |
