# Supplementary material for: CHTOP regulates the NRF2/HO-1 axis to modulate chemoresistance in colorectal cancer
Source: Redox Biol. 2026 Aug 15;96:104353. doi: 10.1016/j.redox.2026.104353 (PMC13521099; doi:10.1016/j.redox.2026.104353)
Supplement: Supplementary file 2 [file mmc2.pdf]

Supplementary Data 2. Target sequences for shRNAs and sequences of the shRNA-resistant construct.

Gene	Target sequences for shRNAs
CHTOP	#1: CAGCTGGACAACCAATTGGAT, TRCN0000139145, Sigma-Aldrich<br>#2: GATGGAGAATAGACCCTCTGT, TRCN0000140444, Sigma-Aldrich<br>#3: GTTAGTCAACACATCTGTAAA, TRCN0000121881, Sigma-Aldrich
PELP1	GAAGAGGATTTGACAGTTATT, TRCN0000159883, Sigma-Aldrich
DUOX2	CCCAACGTCTTTGTGAATGAT, TRCN0000045963, Sigma-Aldrich
SENP1	TGACCATTACACGCAAAGATA, TRCN0000004397, Sigma-Aldrich
SENP2	ACAATGCTGCCAGCTTATTTG, TRCN0000355816, Sigma-Aldrich
SENP3	CTGCCTGCCAGATCTTCAAAC, TRCN0000369030, Sigma-Aldrich
PSIP1	#1: GCAGCTACAGAAGTCAAGATT, TRCN0000286344, Sigma-Aldrich<br>#2: TTTAGGACCAAAGGATATATT, TRCN0000298567, Sigma-Aldrich
NRF2	#1:AGAGCAAGATTTAGATCATTT, TRCN0000281950, Sigma-Aldrich<br>#2:AGTTTGGGAGGAGCTATTATC, TRCN0000281950, Sigma-Aldrich
HO-1	ACAGTTGCTGTAGGGCTTTAT, TRCN0000045250, Sigma-Aldrich

Sequences of the shRNA-resistant Nrf2:

Nrf2-WT:

…ATTCCCAAATCAGAT…...GAATTGGAACAGGACCTGGACCACTTAAAAGAT…

Nrf2-(K110R/K533R) :

…ATTCCCCGTTCAGAT…...GAATTGGAACAGGACCTGGACCACTTACGTGAT…

Sequences of the shRNA-resistant PSIP1/p52:

…TTCCTGGGGCCCAAAGACATTTTTCC…

Sequences of the shRNA-resistant PSIP1/p75:

…TTCCTGGGGCCCAAAGACATTTTTCC…
