# Supplementary material for: CHTOP regulates the NRF2/HO-1 axis to modulate chemoresistance in colorectal cancer
Source: Redox Biol. 2026 Aug 15;96:104353. doi: 10.1016/j.redox.2026.104353 (PMC13521099; doi:10.1016/j.redox.2026.104353)
Supplement: Supplementary file 3 [file mmc3.pdf]

Supplementary data 3. Information on all antibodies.

**Antibody**	**Information**	**Application**
Flag tag	F1804, sigma-aldrich, Darmstadt, Germany	IP, IF,WB
Flag tag	20543-1-AP, Proteintech, Chicago, IL	WB, IP
Myc tag	ab32, Abcam, Cambridge, UK	IP
Myc tag	ab9106, Abcam, Cambridge, UK	WB
Myc tag	16286-1-AP, Proteintech, Chicago, IL	WB
CHTOP	21456-1-AP, Proteintech, Chicago, IL	WB
CHTOP	NBP1-88085, Novus biologicals, Centennial, CO	WB, IF
NRF2	sc-365949, Santa Cruz Biotechnology, Santa Cruz, CA	IP, IF
NRF2	16396-1-AP, Proteintech, Chicago, IL	WB
NRF2	Ab137550, Abcam, Cambridge, UK	WB
Lamin B1	12987-1-AP, Proteintech, Chicago, IL	WB
PELP1	30135-1-AP, Proteintech, Chicago, IL	WB
TEX10	17372-1-AP, Proteintech, Chicago, IL	WB
WDR18	15165-1-AP, Proteintech, Chicago, IL	WB
LAS1L	16010-1-AP, Proteintech, Chicago, IL	WB
SUMO1	sc-5308, Santa Cruz Biotechnology, Santa Cruz, CA	IP
SUMO1	10329-1-AP, Proteintech, Chicago, IL	WB
SUMO2/3	sc-393144, Santa Cruz Biotechnology, Santa Cruz, CA	IP
SUMO2/3	11251-1-AP, Proteintech, Chicago, IL	WB
HO-1	10701-1-AP, Proteintech, Chicago, IL	WB
PSIP1	25504-1-AP, Proteintech, Chicago, IL	WB
HNRNPH1	14774-1-AP, Proteintech, Chicago, IL	IP, IF
HNRNPH1	67375-1-Ig, Proteintech, Chicago, IL	WB
SENP1	ab108981, Abcam, Cambridge, UK	WB
SENP2	ab124724, Abcam, Cambridge, UK	WB
SENP3	ab124790, Abcam, Cambridge, UK	WB
HA	ab1424, Abcam, Cambridge, UK	IP
HA	ab9110, Abcam, Cambridge, UK	WB
GST tag	10000-0-AP, Proteintech, Chicago, IL	WB
α-Tubulin	66031-1-Ig, Proteintech, Chicago, IL	WB
Vinculin	sc-73614, Santa Cruz Biotechnology, Santa Cruz, CA	WB
HRP-conjugated Goat Anti-Rabbit IgG	abs20147, Absin, Shanghai, China	Secondary antibody, WB
HRP-conjugated Goat Anti-Mouse IgG	abs20001, Absin, Shanghai, China	Secondary antibody, WB
FITC–conjugated Goat Anti-Mouse IgG	SA00003-1, Proteintech, Chicago, IL	Secondary antibody, IF
Cy3–conjugated Goat Anti-Rabbit IgG	SA00009-2, Proteintech, Chicago, IL	Secondary antibody, IF
