# Supplementary material for: CHTOP regulates the NRF2/HO-1 axis to modulate chemoresistance in colorectal cancer
Source: Redox Biol. 2026 Aug 15;96:104353. doi: 10.1016/j.redox.2026.104353 (PMC13521099; doi:10.1016/j.redox.2026.104353)
Supplement: Supplementary file 4 [file mmc4.pdf]

Supplementary Data 4. Primers for RT-qPCR and PCR

For RT-qPCR

1	CHTOP-F	TTAAAGCAGCGCCTGGGTAA
2	CHTOP-R	GAGCAGGTTTTGACCTCGGA
3	P52-1-F	GCAGATCGAAAACGCAAGCA
4	P52-1-R	GGGCCCCATTCTCAGGGATA
5	P52-2-F	AGCAGATCGAAAACGCAAGC
6	P52-2-R	CAGGAATGATGGCCGACCTTA
7	HO-1-F	GTGCCACCAAGTTCAAGCAG
8	HO-1-R	GCAACTCCTCAAAGAGCTGGA
9	GCLC-F	GTTCTCAAGTGGGGCGATGA
10	GCLC-R	CCGGCTTAGAAGCCCTTGAA
11	NQO1-F	TGCTTACACTTACGCTGCCAT
12	NQO1-R	CCAGTGGTGATGGAAAGCAC
13	GCLM-F	TTTGGAATCAGCTCTCCCGC
14	GCLM-R	CTCGTGCGCTTGAATGTCAG
15	TXNRD1-F	CTCAAATTCTTAGGACGGTCGGG
16	TXNRD1-R	AAGATCTTCAGGGCCGTTCAT
17	α-Tubulin-F	ACCTGATGTATGCCAAGCGT
18	α-Tubulin-R	AGCTGAAATTCTGGGAGCATGA

For PCR

1	CHTOP2-F	GAGCTCGCATACTACCGCTT
2	CHTOP2-R	TGACCTCTTCCAGCAGTTCG
3	α-Tubulin-F	ACCTGATGTATGCCAAGCGT
4	α-Tubulin-R	AGCTGAAATTCTGGGAGCATGA
