# Supplementary material for: CHTOP regulates the NRF2/HO-1 axis to modulate chemoresistance in colorectal cancer
Source: Redox Biol. 2026 Aug 15;96:104353. doi: 10.1016/j.redox.2026.104353 (PMC13521099; doi:10.1016/j.redox.2026.104353)
Supplement: Supplementary file 5 [file mmc5.pdf]

Figure S1

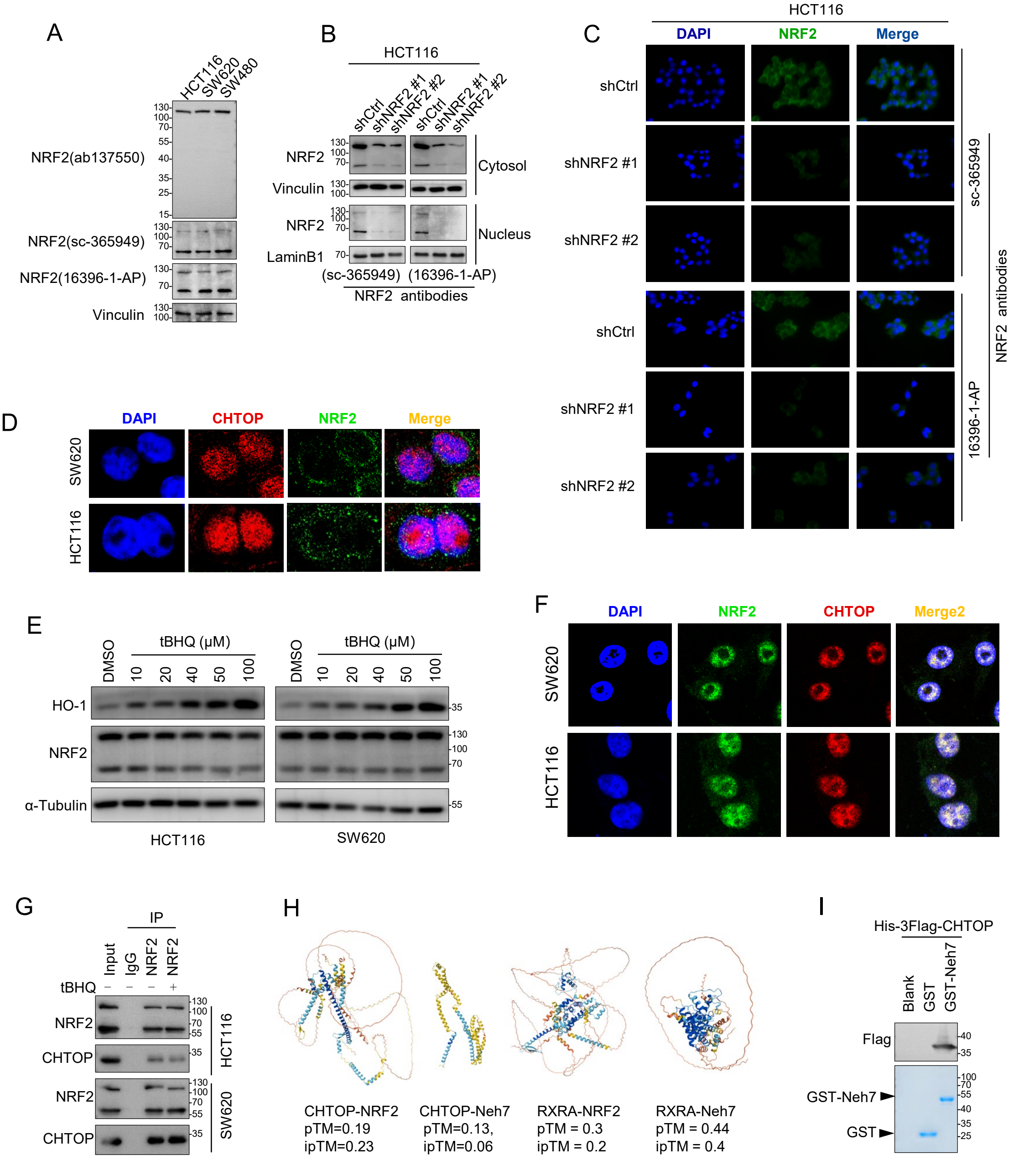

Supplementary Figure 1.

A. Western blot analysis of NRF2 protein levels in HCT116, SW480 and SW620 cells using three commercially available anti-NRF2 antibodies (Abcam ab137550, Santa Cruz sc-365949, and Proteintech 16396-1-AP). Abcam ab137550 detected a single band at approximately 110 kDa, whereas Santa Cruz sc-365949 and Proteintech 16396-1-AP both detected an additional lower band at approximately 68 kDa. **B.** Nuclear-cytoplasmic fractionation followed by Western blot analysis of NRF2 in HCT116 cells transfected with control shRNA (shCtrl) or two independent NRF2-targeting shRNAs. Both the 110 kDa and 68 kDa bands were markedly reduced in both fractions upon NRF2 knockdown. **C.** Immunofluorescence staining of NRF2 in HCT116 cells transfected with control shRNA (shCtrl) or NRF2-targeting shRNAs. Knockdown of NRF2 markedly reduced the fluorescence signal, confirming antibody specificity. **D.** Confocal microscopy analysis of NRF2 and CHTOP subcellular localization in untreated HCT116 and SW620 cells. NRF2 was predominantly cytoplasmic with weak nuclear signal, whereas CHTOP was mainly nuclear; their colocalization was modest under basal conditions. **E.** Western blot analysis of total NRF2 and HO-1 protein levels in HCT116 and SW620 cells treated with increasing concentrations of tBHQ for 24 h. Total NRF2 levels remained largely unchanged, whereas HO-1 showed a dose-dependent increase. **F.** Confocal microscopy analysis of NRF2 and CHTOP colocalization in HCT116 and SW620 cells treated with 50 μM tBHQ for 24 h. tBHQ treatment markedly induced NRF2 nuclear translocation and enhanced its colocalization with CHTOP. **G.** Co-IP analysis of CHTOP-NRF2 interaction in HCT116 and SW620 cells treated with vehicle or 50 μM tBHQ. TBHQ treatment did not substantially alter the CHTOP-NRF2 interaction. **H.** AlphaFold-Multimer predicted model of the indicated complex. The model is colored by pLDDT confidence score (blue: high confidence; red: low confidence). For each prediction, the pTM and ipTM scores are indicated. **I.** In vitro GST pull-down assay using purified GST-Neh7 and His-3×Flag-CHTOP proteins. CHTOP was specifically retained by GST-Neh7 but not by GST tag.
