# Supplementary material for: CHTOP regulates the NRF2/HO-1 axis to modulate chemoresistance in colorectal cancer
Source: Redox Biol. 2026 Aug 15;96:104353. doi: 10.1016/j.redox.2026.104353 (PMC13521099; doi:10.1016/j.redox.2026.104353)
Supplement: Supplementary file 6 [file mmc6.pdf]

Figure S2

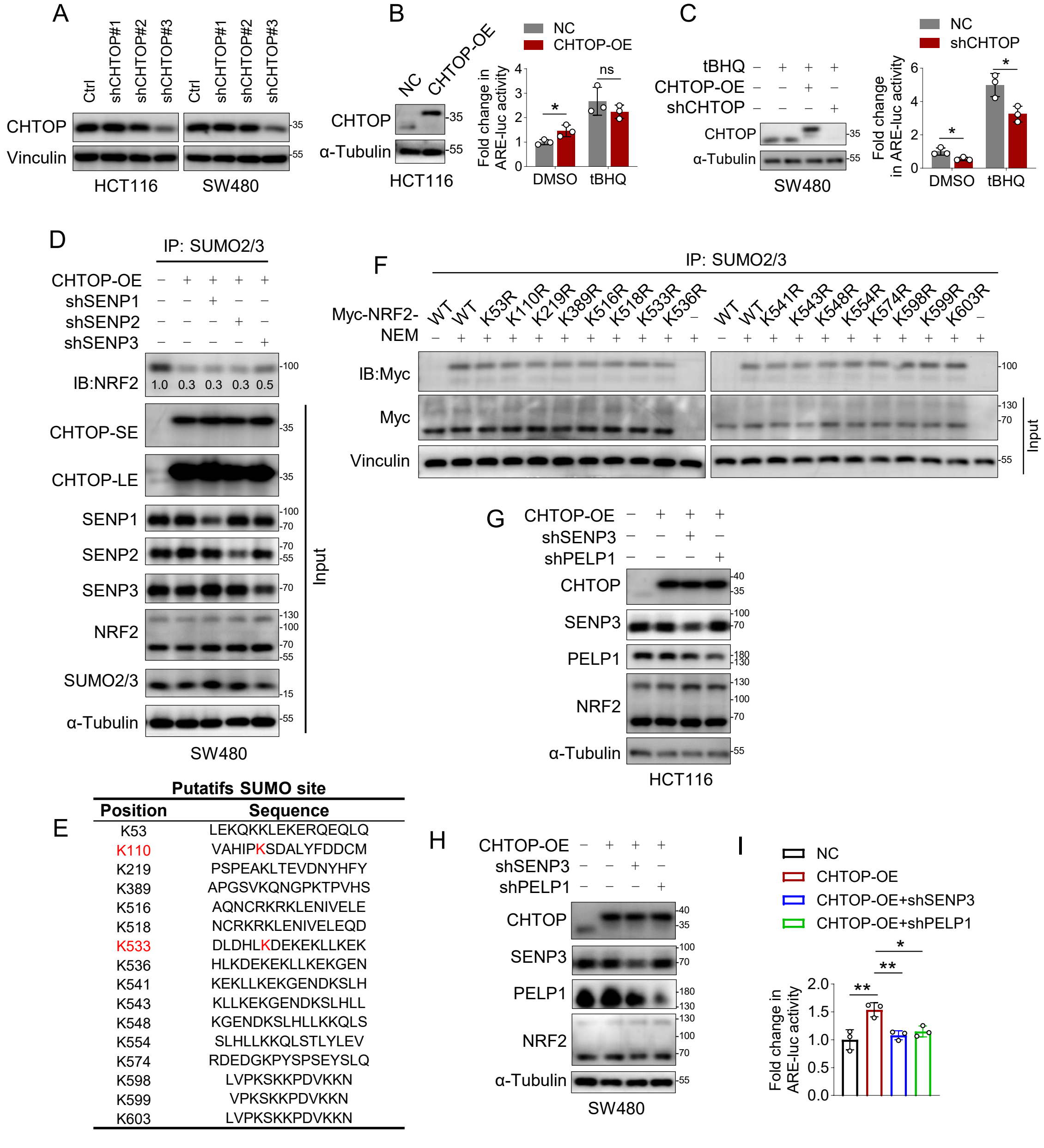

E

Putatifs SUMO site

Position	Sequence
K53	LEKQKKLEKERQEQLQ
K110	VAHIPKSDALYFDDCM
K219	PSPEAKLTEVDNYHFY
K389	APGSVKQNGPKTPVHS
K516	AQNCRKRKLENIVELE
K518	NCRKRKLENIVELEQD
K533	DLDHLKDEKEKLLKEK
K536	HLKDEKEKLLKEKGEN
K541	KEKLLKEKGENDKSLH
K543	KLLKEKGENDKSLHLL
K548	KGENDKSLHLLKKQLS
K554	SLHLLKKQLSTLYLEV
K574	RDEDGKPYSPSEYSLQ
K598	LVPKSKKPDVKKN
K599	VPKSKKPDVKKN
K603	LVPKSKKPDVKKN

Supplementary Figure 2.

A. Western blot analysis of CHTOP protein levels in HCT116 cells transfected with three independent CHTOP-targeting shRNAs. **B.** CHTOP overexpression in HCT116 cells (left) and the effect on basal or tBHQ (50 μM for 24 hours) induced ARE activity (right). **C.** CHTOP overexpression or knockdown in SW480 cells (left) and the corresponding effects on basal or tBHQ-induced (50 μM, 24 h) ARE activity (right). **D.** SW480 cells with CHTOP overexpression alone or combined with SENP1, SENP2, or SENP3 knockdown were subjected to immunoprecipitation with anti-SUMO2/3 antibody, followed by immunoblotting with anti-NRF2 antibody. **E.** Putative SUMOylation sites in NRF2 predicted by the Joined Advanced Sumoylation Site and SIM Analyzer. **F.** HEK293T cells expressing Myc-NRF2 wild-type (WT) or the indicated point mutants were subjected to immunoprecipitation with anti-SUMO2/3 antibody, followed by immunoblotting with anti-Myc antibody. **G-H.** CHTOP overexpression alone or with SENP3 or PELP1 knockdown in HCT116 (G) or SW480 (H) cells. **I.** ARE luciferase activity in cells with CHTOP overexpression alone or combined with SENP3 or PELP1 knockdown. Data are expressed as mean ± SD, n = 3 biological replicates. *P<0.05, **P<0.01.
