# Supplementary material for: CHTOP regulates the NRF2/HO-1 axis to modulate chemoresistance in colorectal cancer
Source: Redox Biol. 2026 Aug 15;96:104353. doi: 10.1016/j.redox.2026.104353 (PMC13521099; doi:10.1016/j.redox.2026.104353)
Supplement: Supplementary file 7 [file mmc7.pdf]

Figure S3

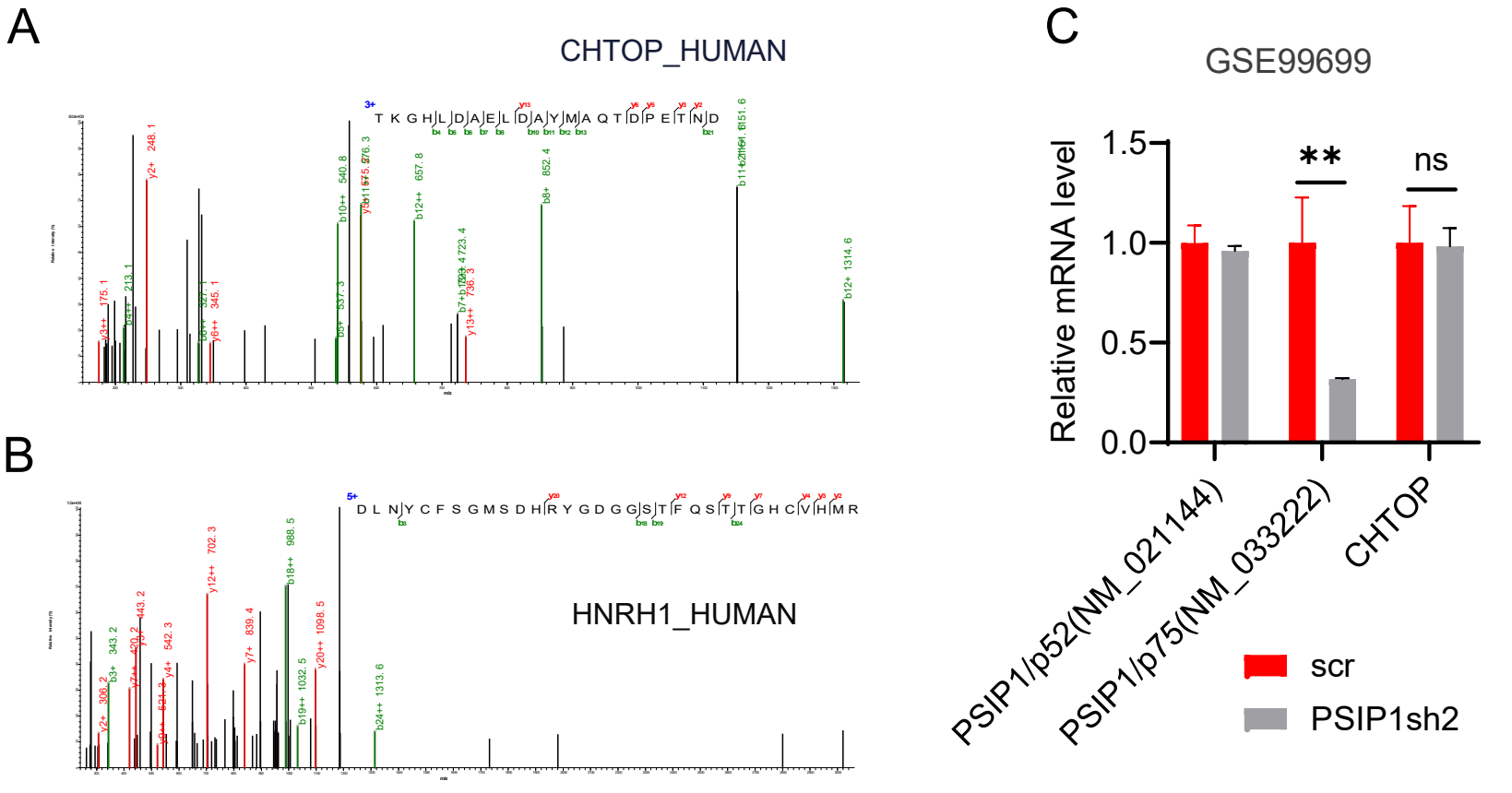

Supplementary Figure 3.

A. Mass spectrometry spectrum of a peptide derived from CHTOP. **B**. Mass spectrometry spectrum of a peptide derived from HNRNPH1. **C**. GEO dataset showing the alteration of CHTOP mRNA after p75 knockdown (without p52 knockdown). Data are expressed as mean ± SD, n = 3 biological replicates. ***P*<0.01.
