# Supplementary material for: CHTOP regulates the NRF2/HO-1 axis to modulate chemoresistance in colorectal cancer
Source: Redox Biol. 2026 Aug 15;96:104353. doi: 10.1016/j.redox.2026.104353 (PMC13521099; doi:10.1016/j.redox.2026.104353)
Supplement: Supplementary file 8 [file mmc8.pdf]

Figure S4

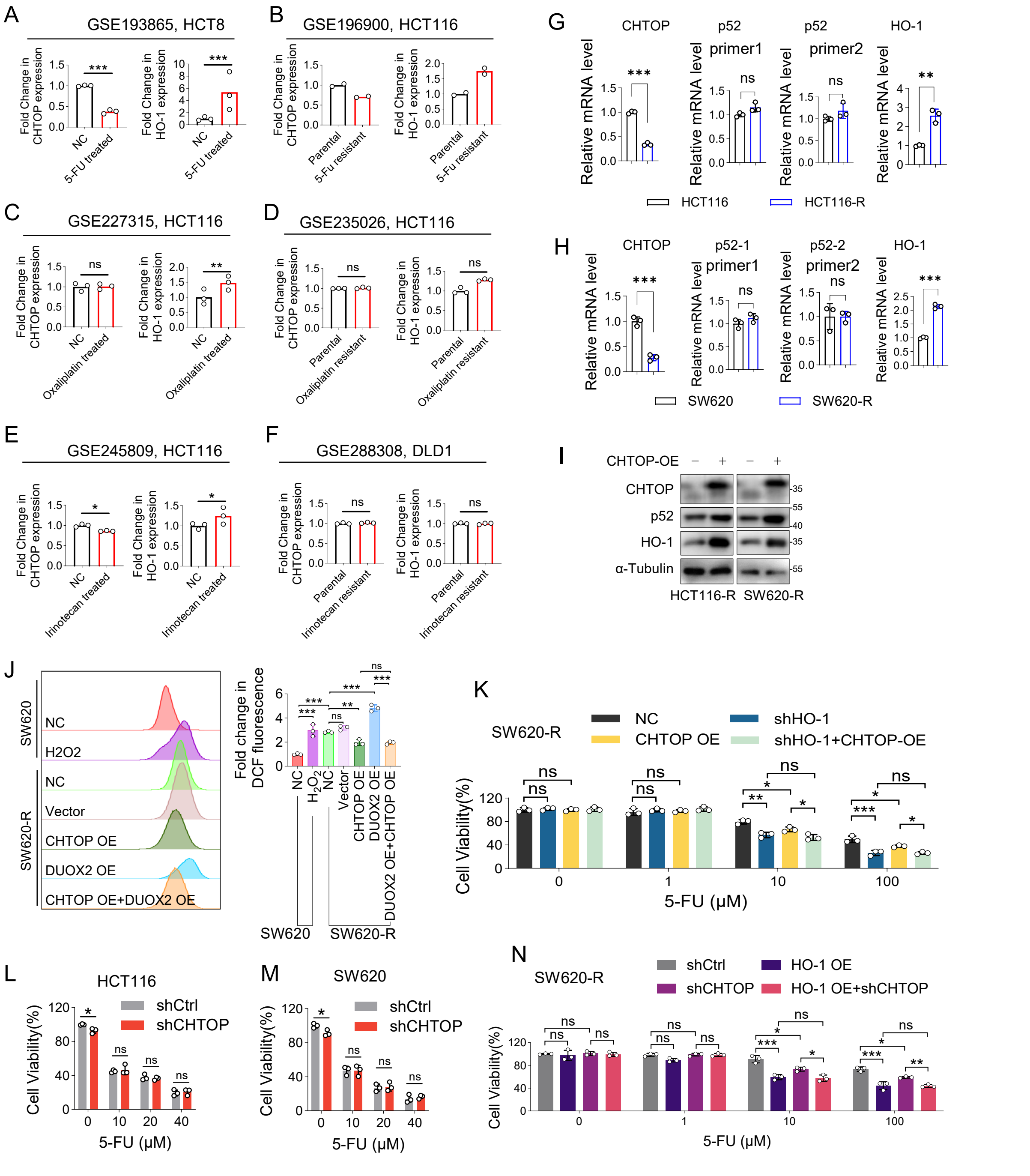

Supplementary Figure 4.

A-F. GEO dataset showing alterations in CHTOP and HO-1 mRNA levels after 5-FU treatment or the development of acquired 5-FU resistance. **G-H.** Relative mRNA levels of CHTOP, p52, and HO-1 in HCT116-R (G) and SW620-R (H) cells compared to their respective parental controls. **I.** Protein alterations of p52 and HO-1 in cells following CHTOP overexpression. **J**. Flow cytometry analysis of DCF fluorescence in SW620 and SW620-R cells expressing the indicated constructs. H_2O_2 (100 μM, 4 h) served as a positive control. **K.** MTT assay of SW620-R cells treated with increasing concentrations of 5-FU (0, 1, 10, 100 μM) following HO-1 knockdown, CHTOP overexpression, or both. **L-M.** The effect of CHTOP knockdown on the response to 5-FU in HCT116 (**L**) and SW620 (**M**) cells. **N.** MTT assay of SW620-R cells treated with increasing concentrations of 5-FU (0, 1, 10, 100 μM) following HO-1 overexpression, CHTOP knockdown, or both. Data are expressed as mean ± SD, n = 3 biological replicates. *P<0.05, **P<0.01, ***P<0.001.
