# Supplementary material for: CHTOP regulates the NRF2/HO-1 axis to modulate chemoresistance in colorectal cancer
Source: Redox Biol. 2026 Aug 15;96:104353. doi: 10.1016/j.redox.2026.104353 (PMC13521099; doi:10.1016/j.redox.2026.104353)

Fig.1B

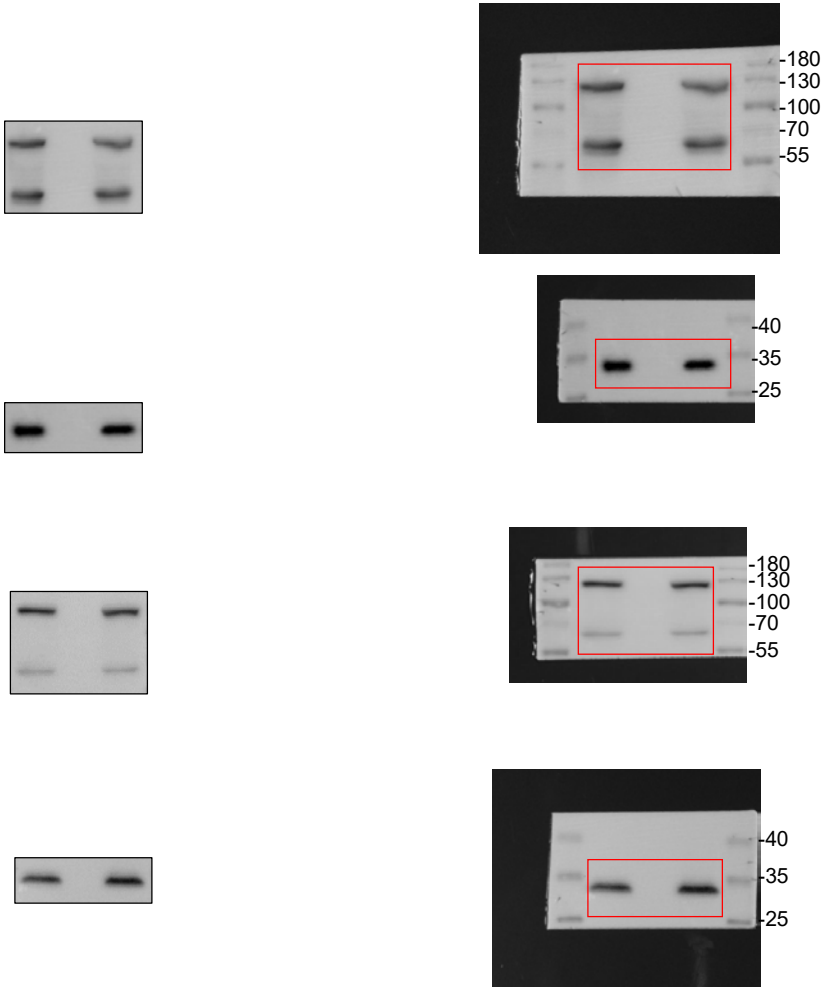

Fig.1C

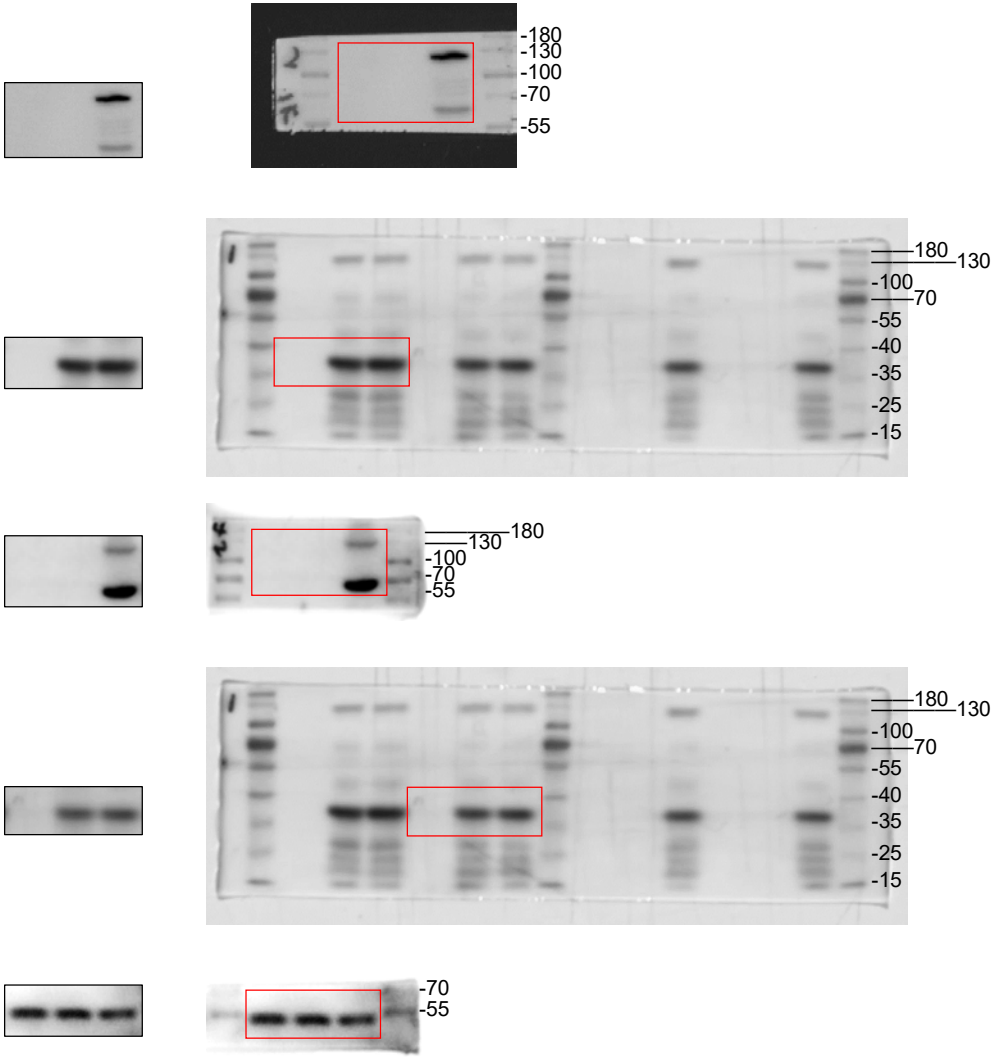

-180
-130
-100
-70
-55
180
130
-100
70
-55
-40
-35
-25
-15
180
130
-100
-70
-55
180
130
-100
70
-55
-40
-35
-25
-15
-70
-55

Fig.1D

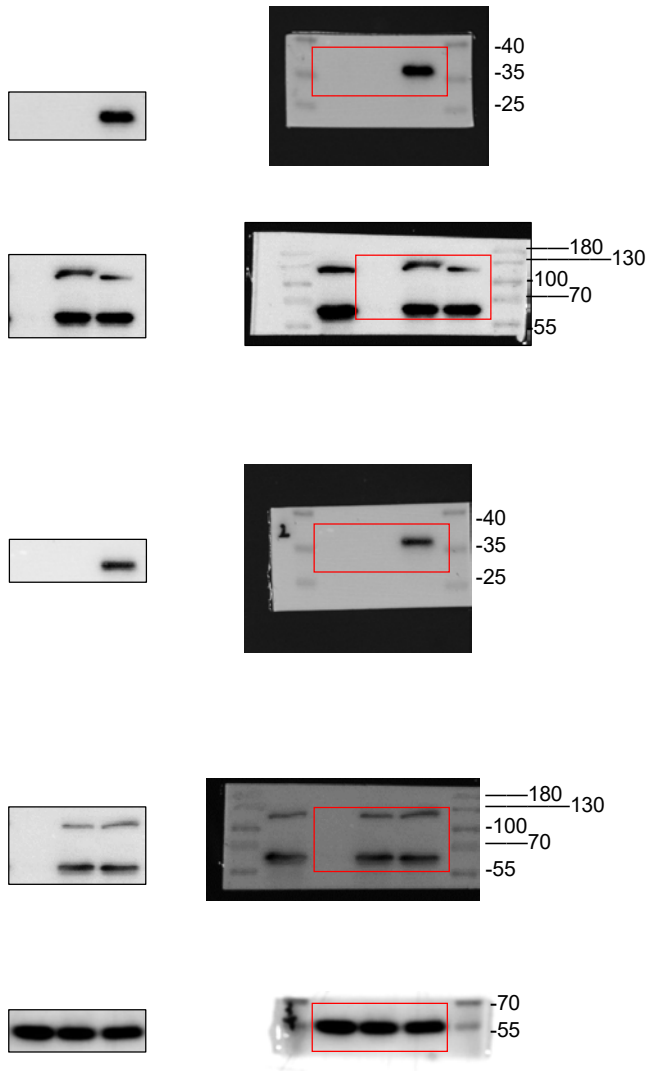

Fig.1E

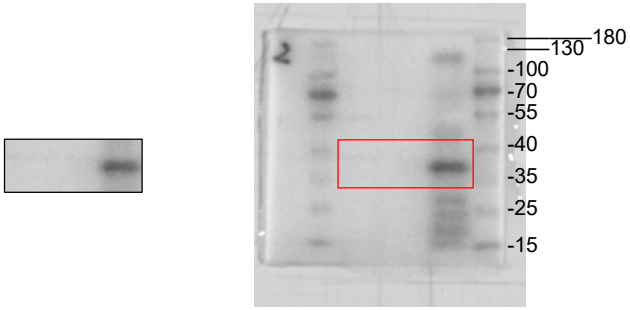

Fig.1G

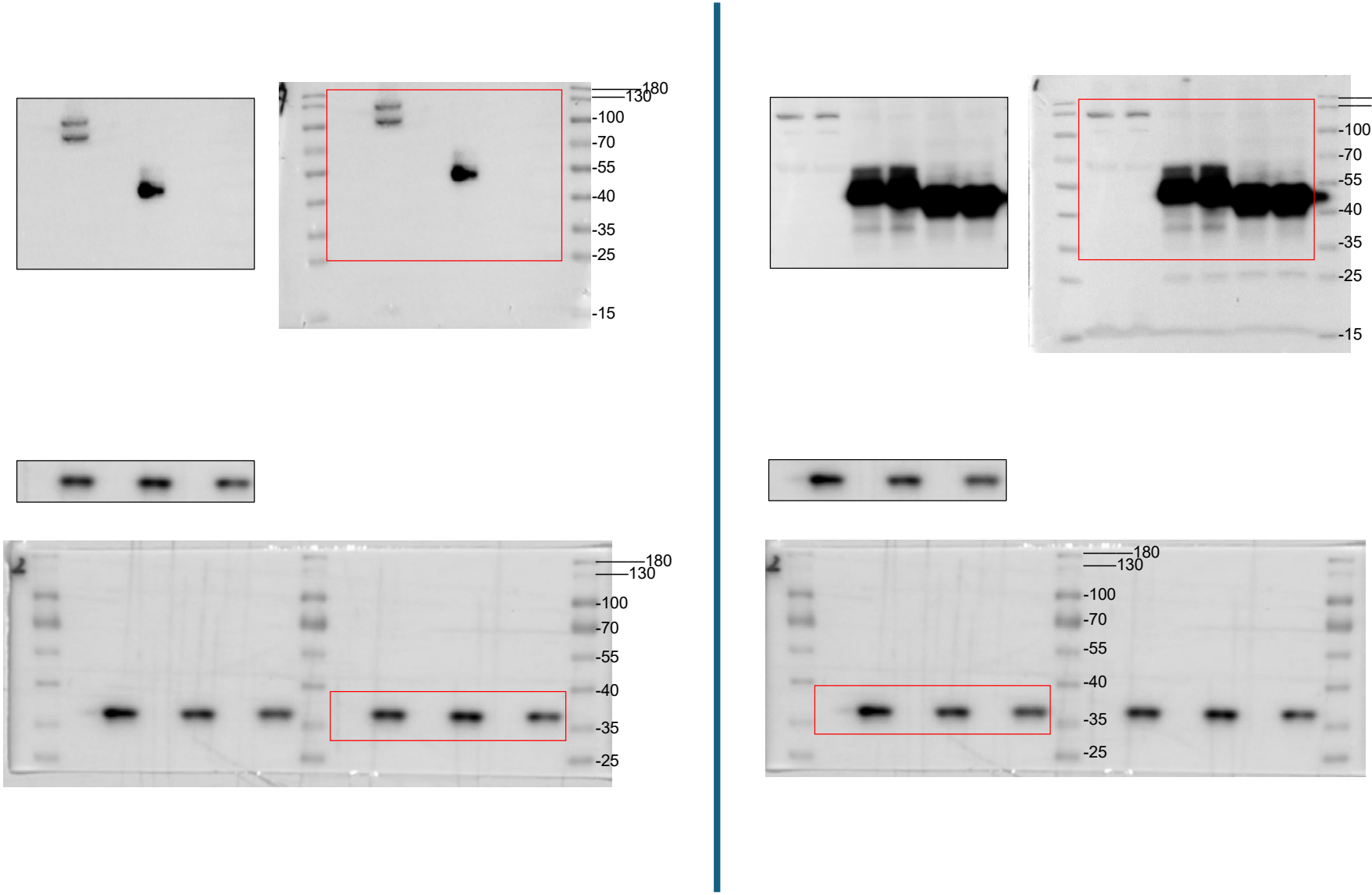

Fig.1H

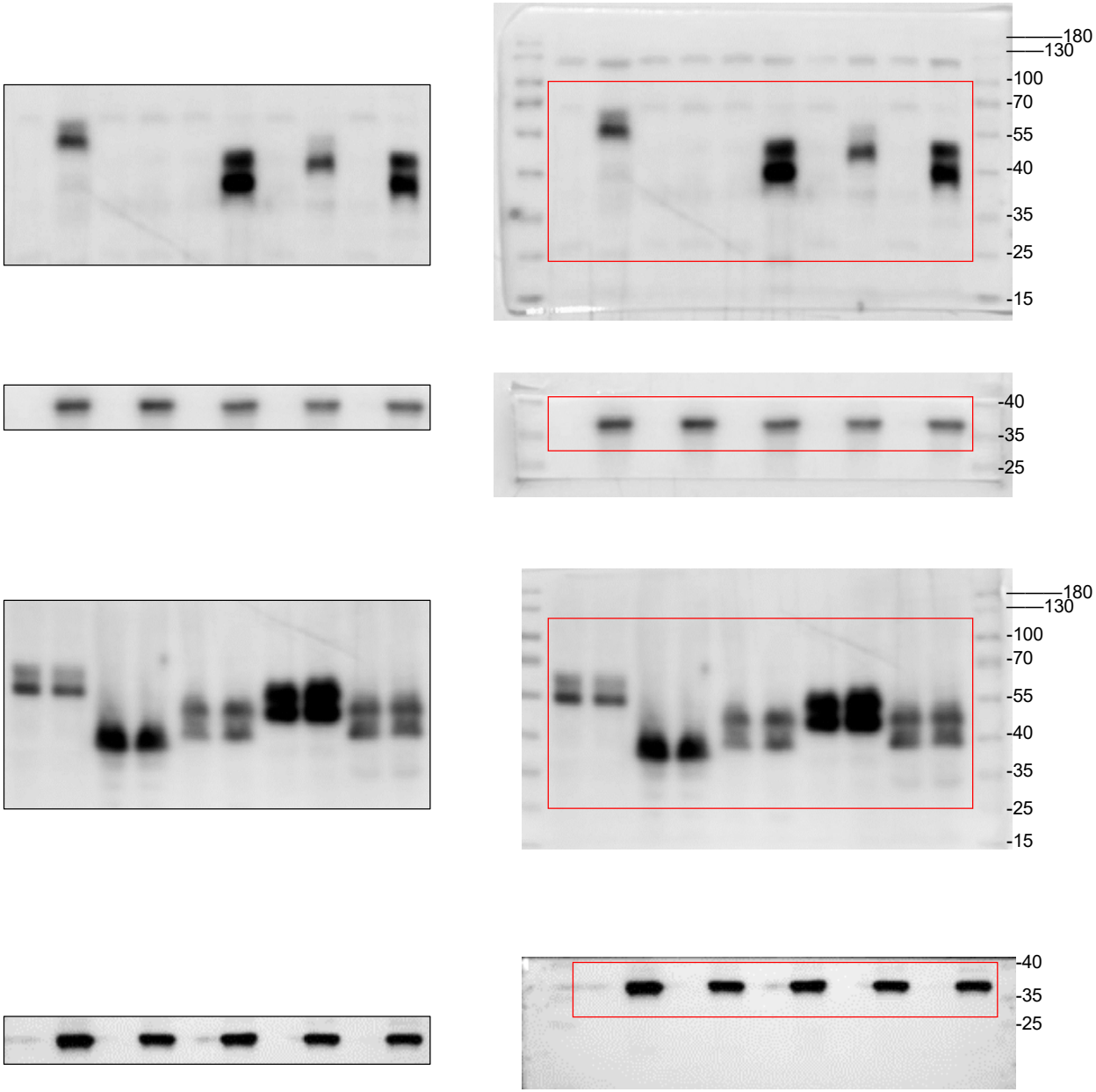
180
130
100
70
55
40
35
25
15
40
35
25
180
130
100
70
55
40
35
25
15
40
35
25

Fig.1l

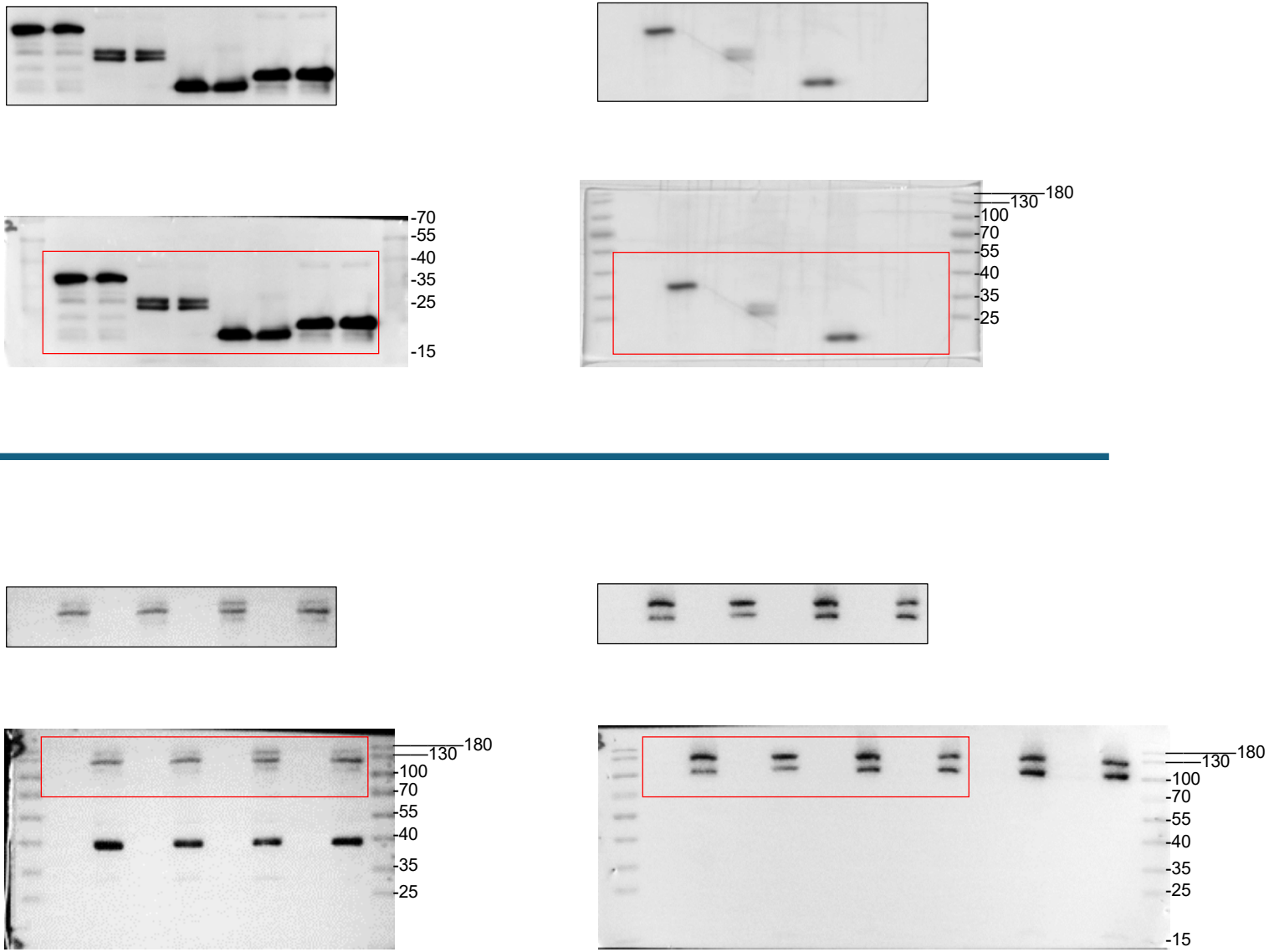

-70
-55
-40
-35
-25
-15
180
130
-100
-70
-55
-40
-35
-25
180
130
-100
-70
-55
-40
-35
-25
180
130
-100
-70
-55
-40
-35
-25
-15

Fig.2A

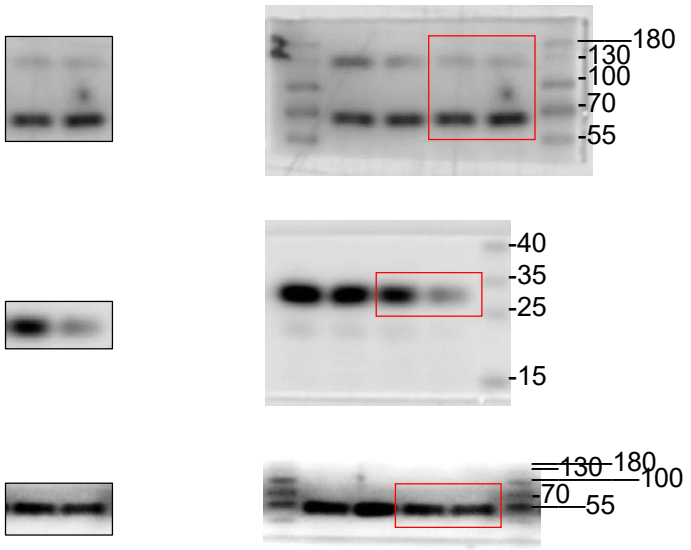

Fig.2B

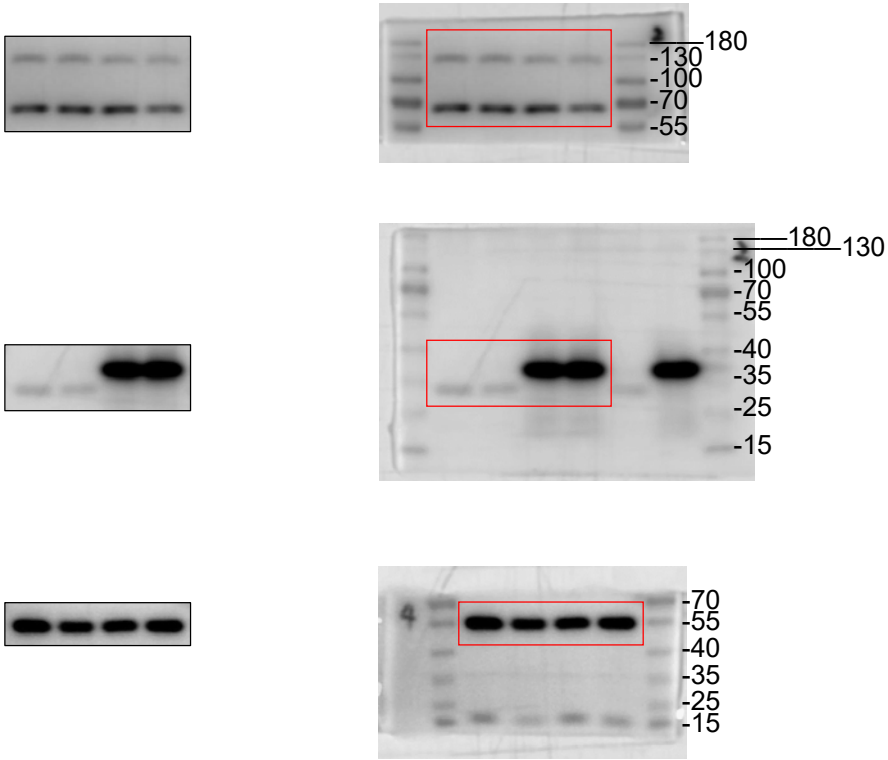

Fig.2C

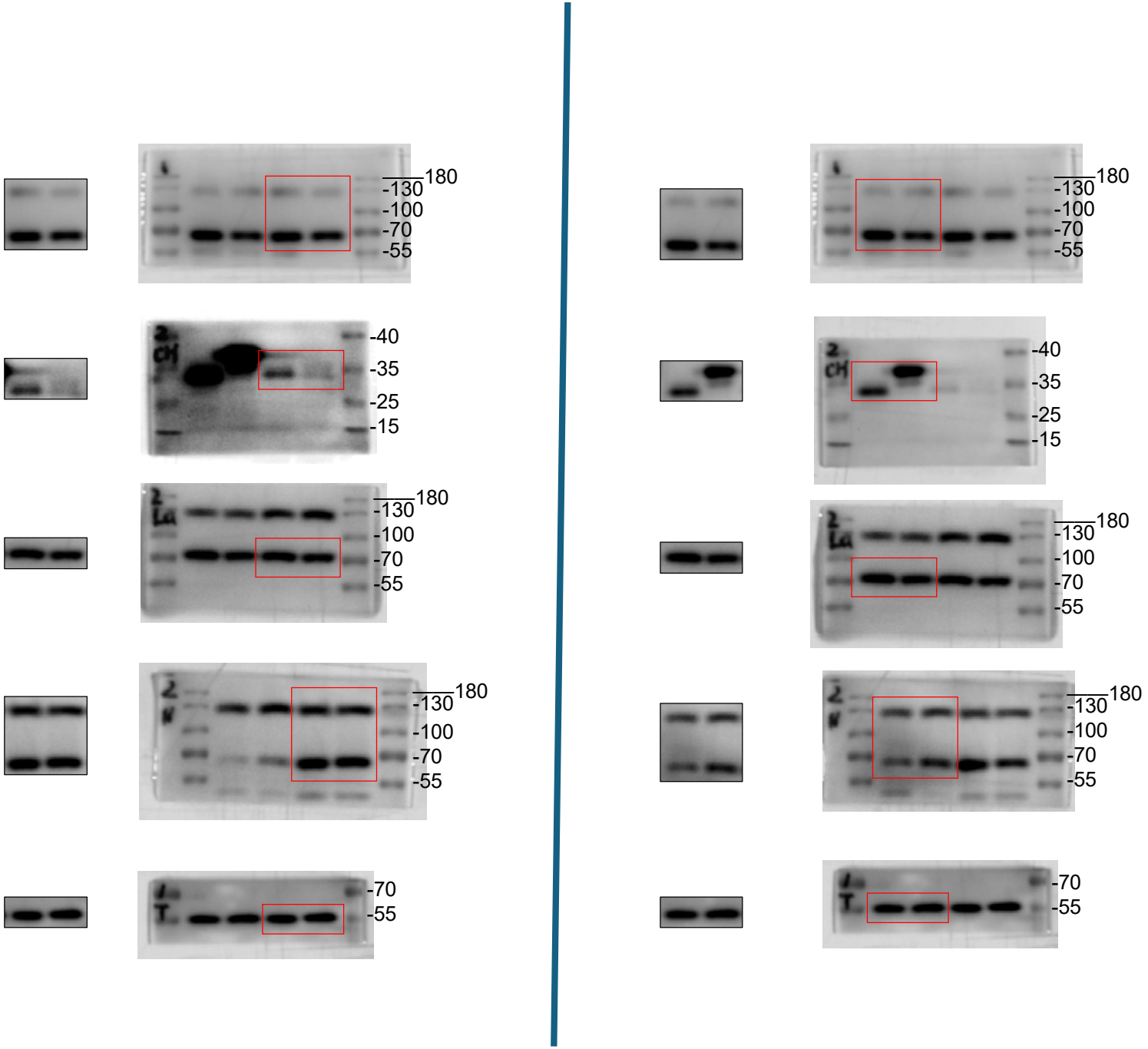
180
-130
-100
-70
-55
-40
-35
-25
-15
180
-130
-100
-70
-55
180
-130
-100
-70
-55
-70
-55
180
-130
-100
-70
-55
-40
-35
-25
-15
180
-130
-100
-70
-55
180
-130
-100
-70
-55
-70
-55

Fig.2D

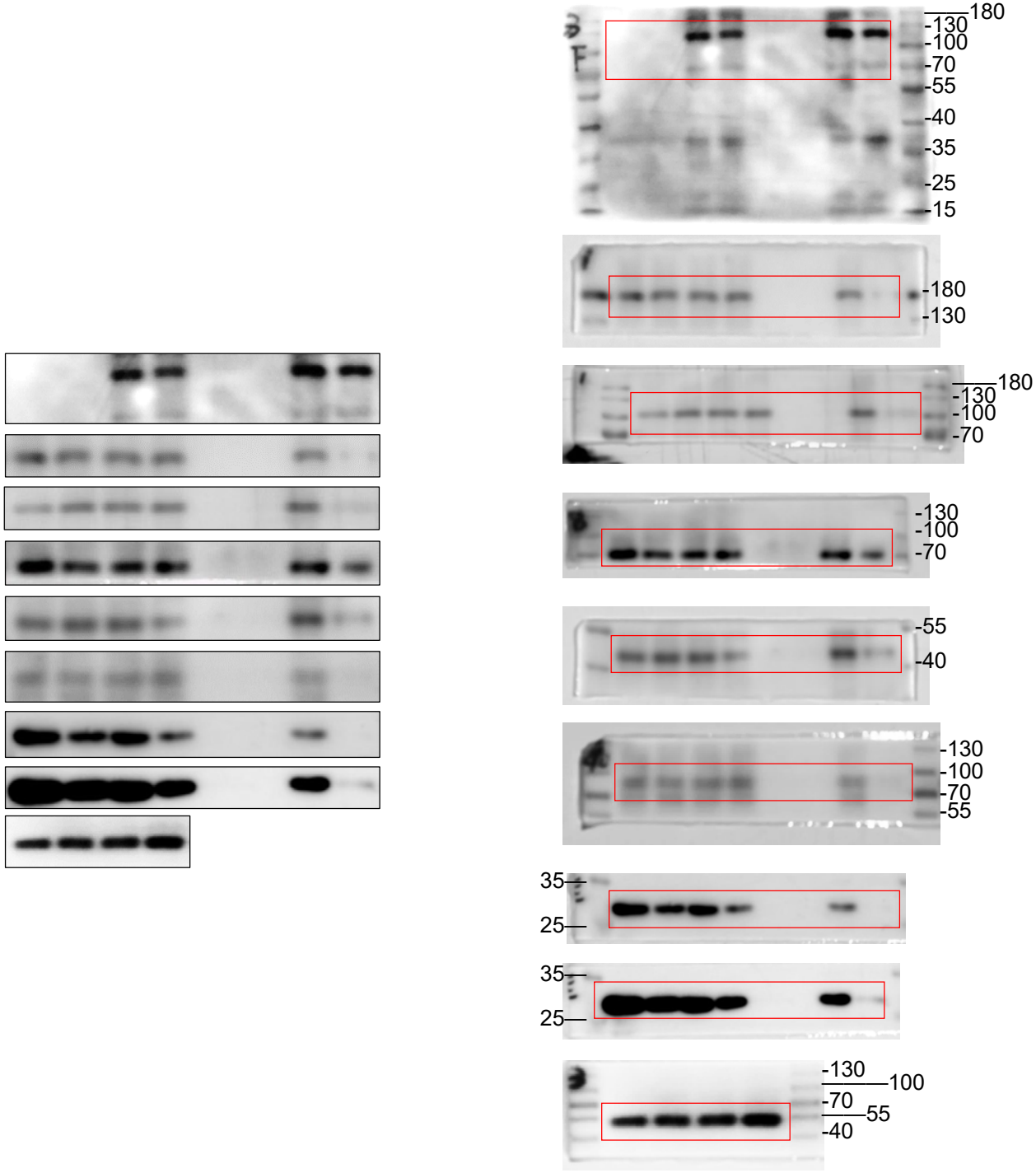

Fig.2E

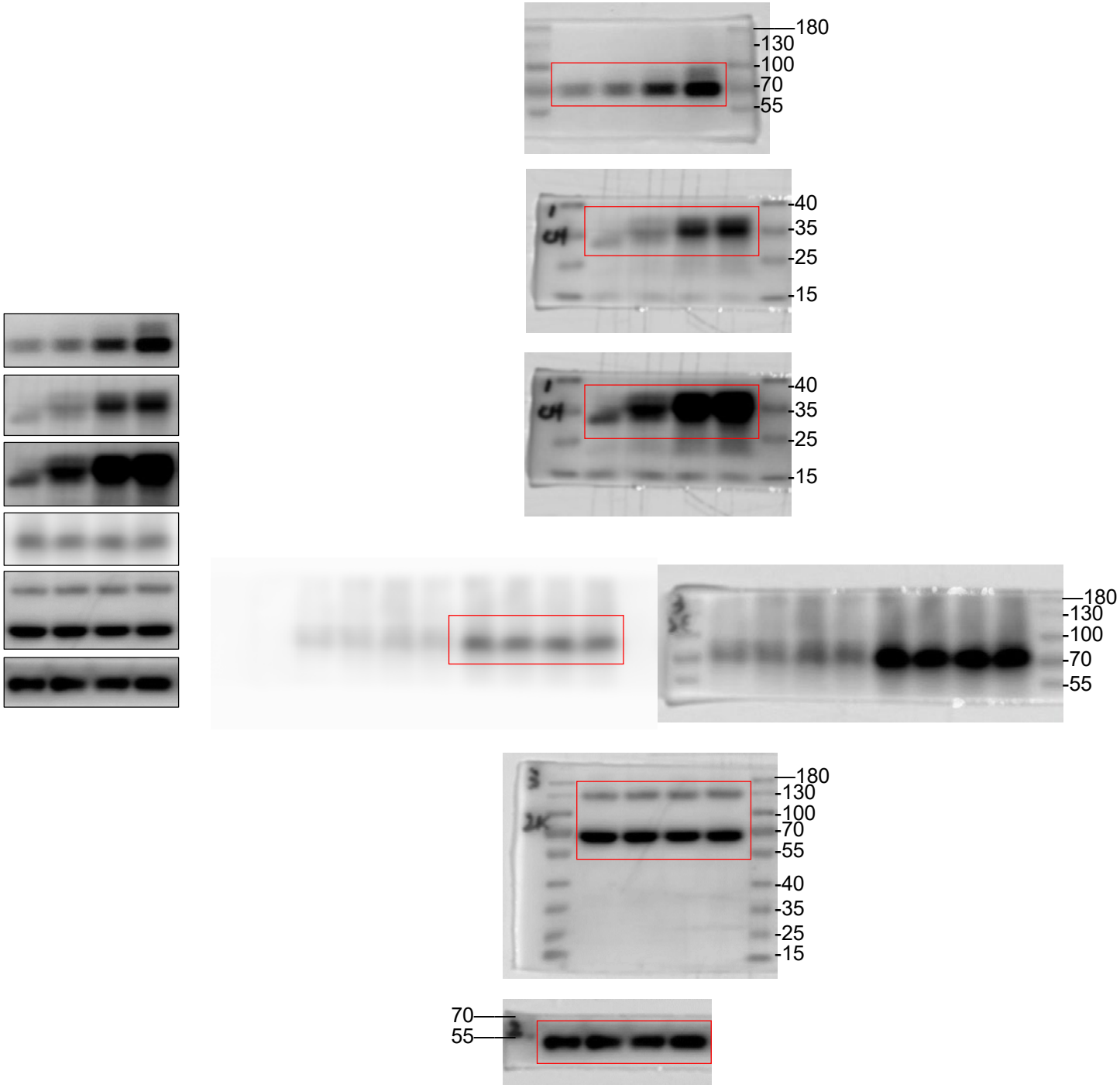

180
130
100
70
55
40
35
25
15
40
35
25
15
180
130
100
70
55
180
130
100
70
55
40
35
25
15
70
55

Fig.2F

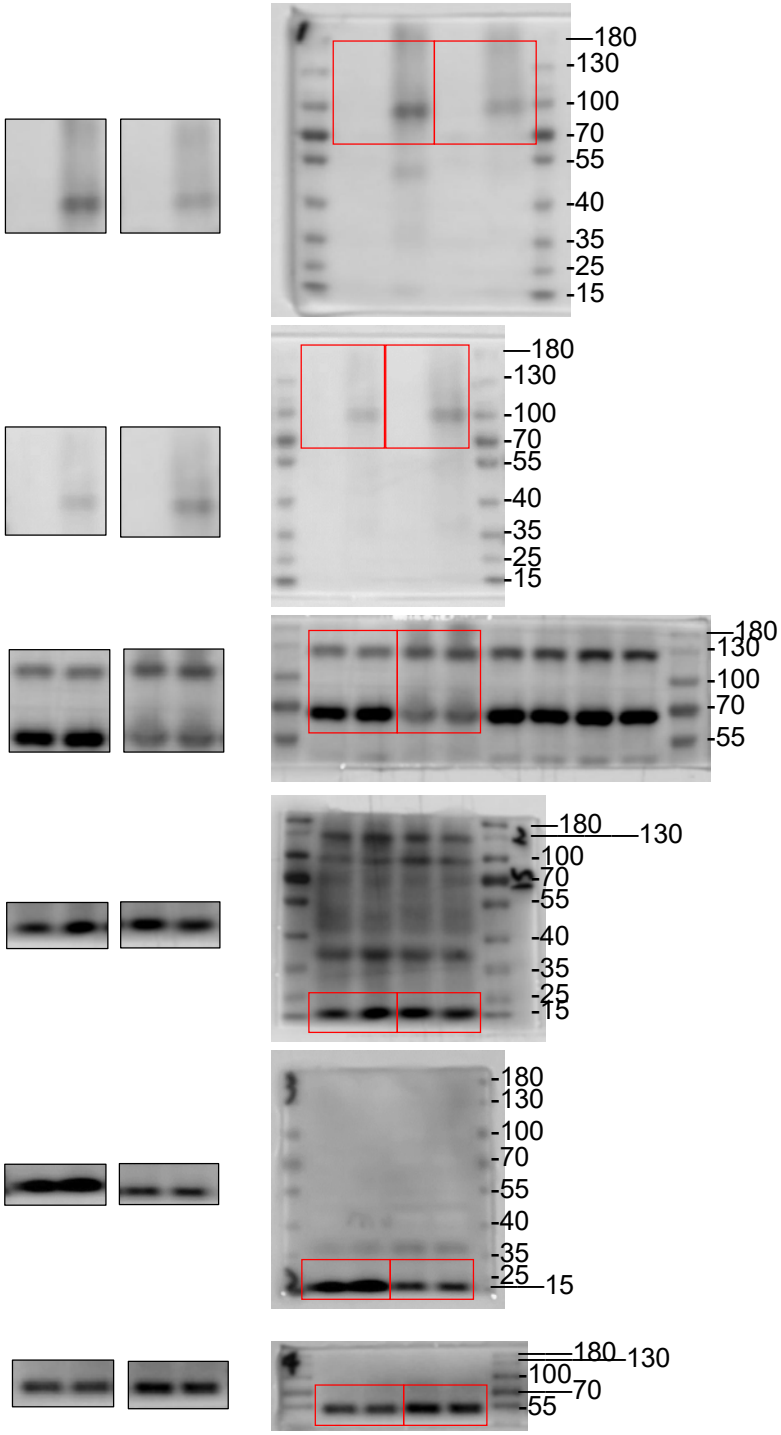

Fig.2G

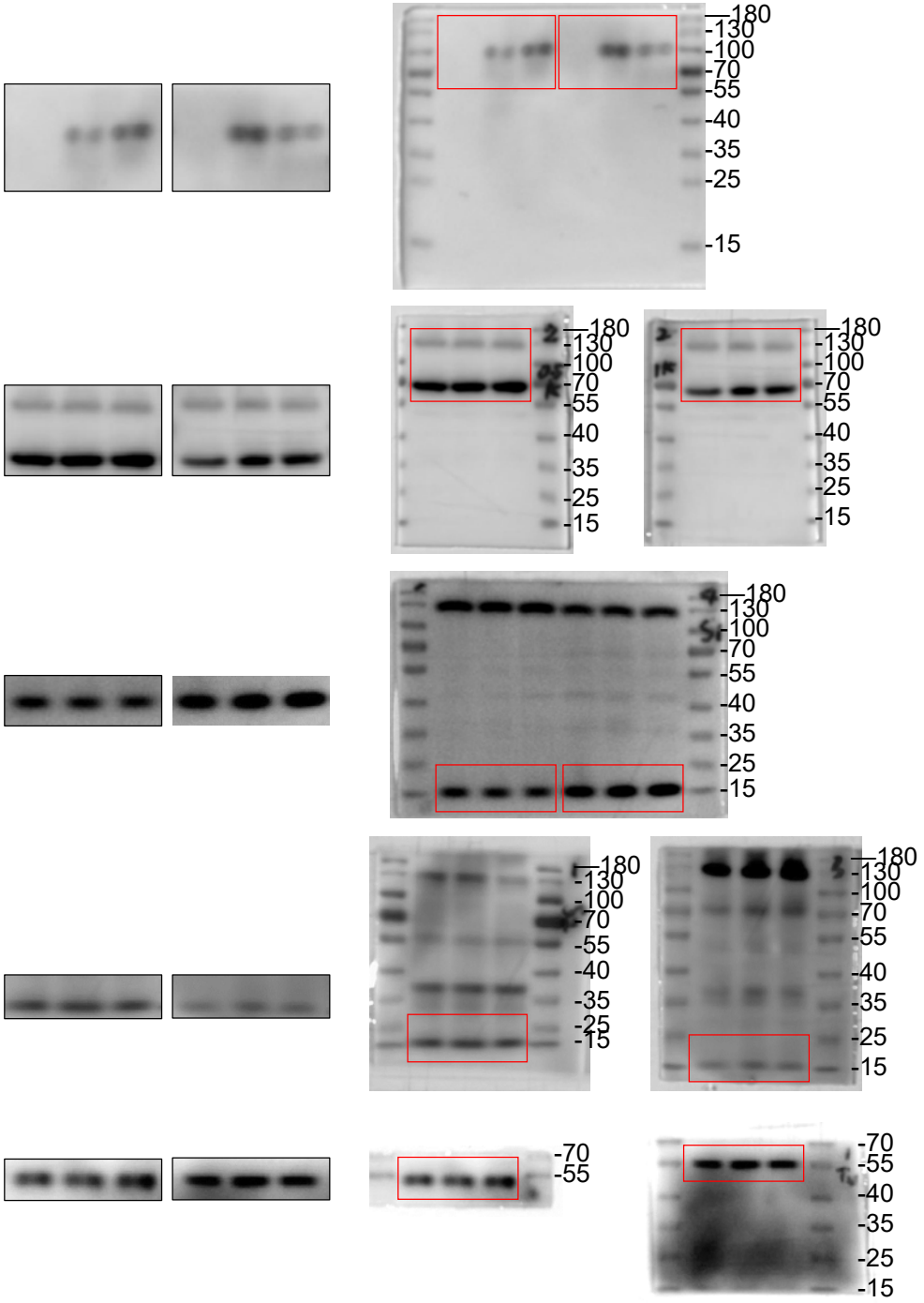

Fig.2H

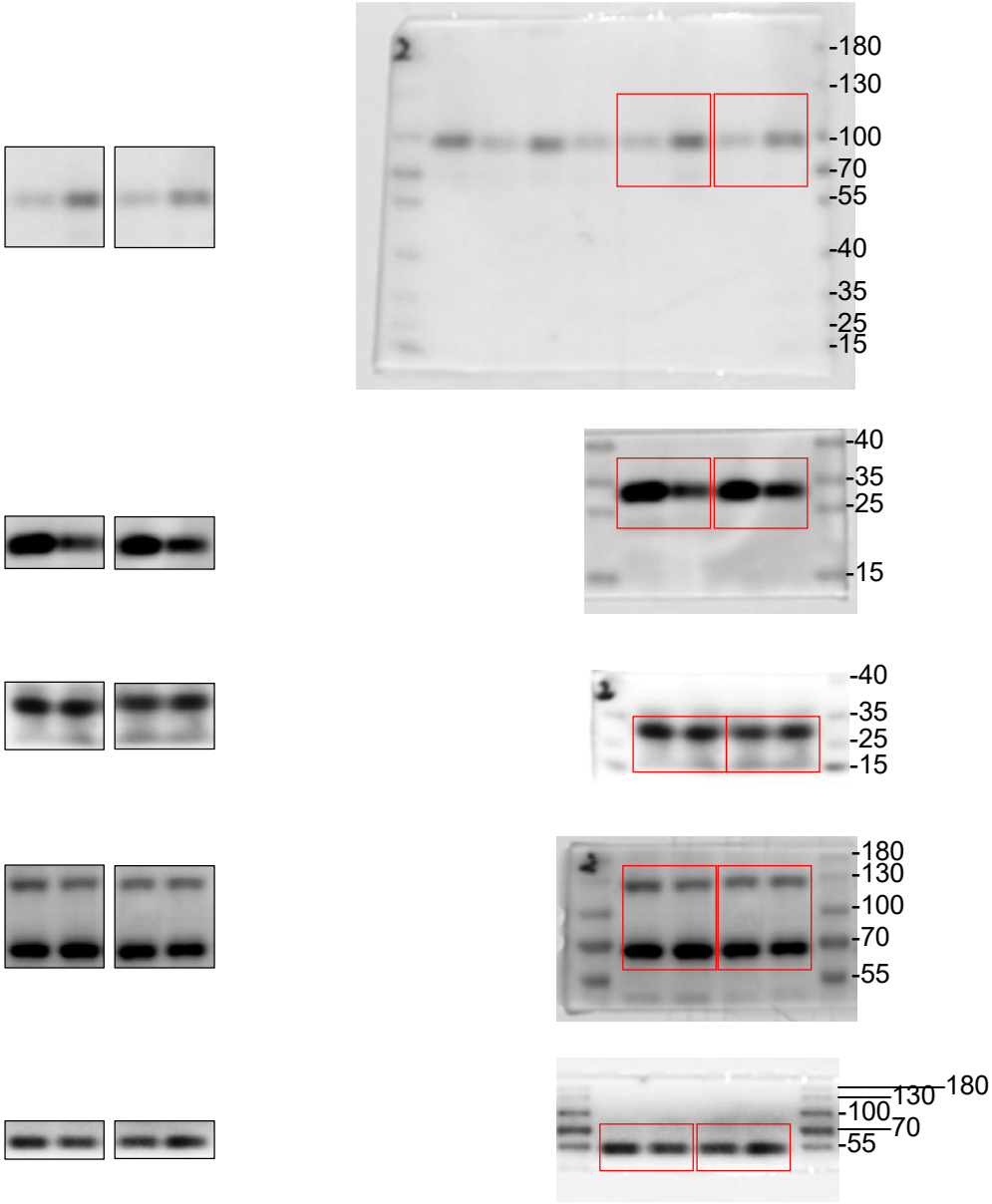

-180
-130
-100
-70
-55
-40
-35
-25
-15
-40
-35
-25
-15
-40
-35
-25
-15
-180
-130
-100
-70
-55
180
130
-100
70
-55

Fig.2I

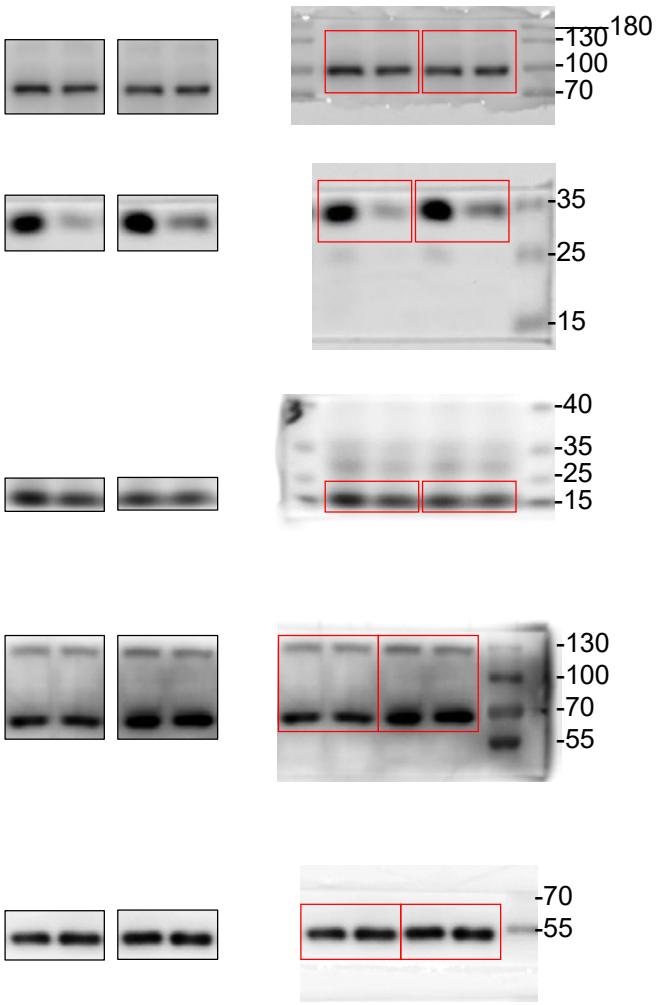

Fig.2J

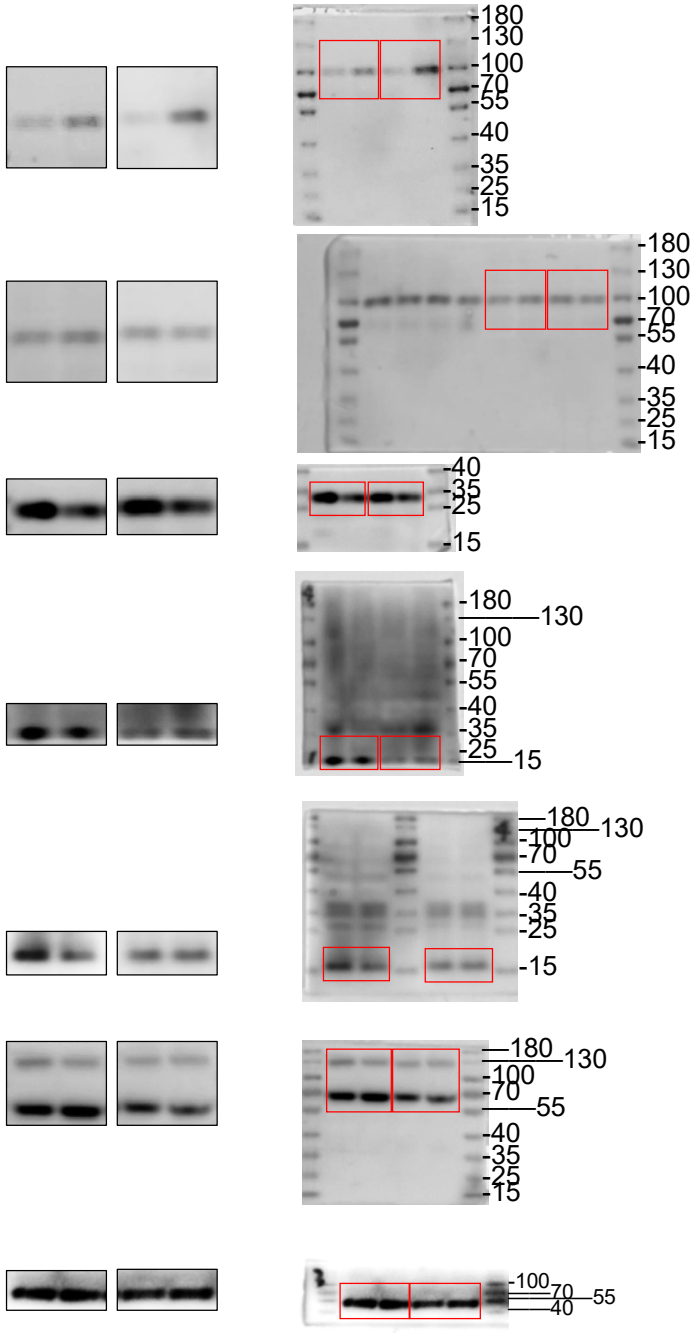

Fig.2K

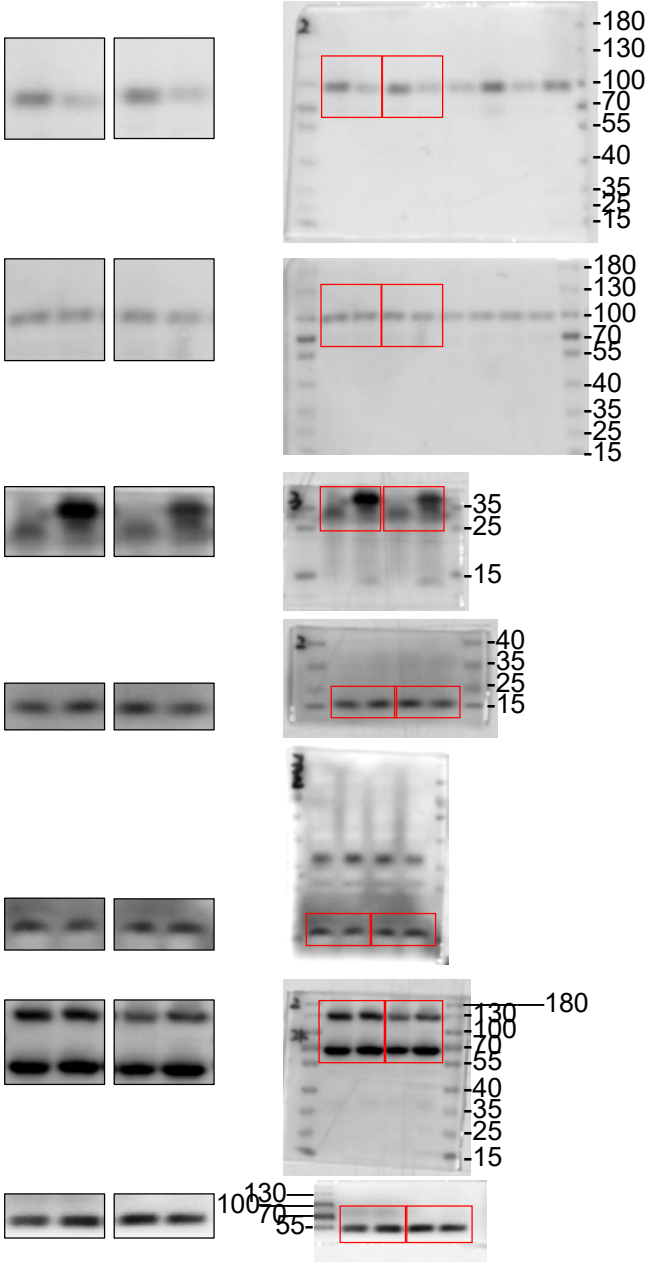

-180
-130
-100
-70
-55
-40
-35
-25
-15
-180
-130
-100
-70
-55
-40
-35
-25
-15
-35
-25
-15
-40
-35
-25
-15
180
-130
-100
-70
-55
-40
-35
-25
-15
130
100
70
55

Fig.2L

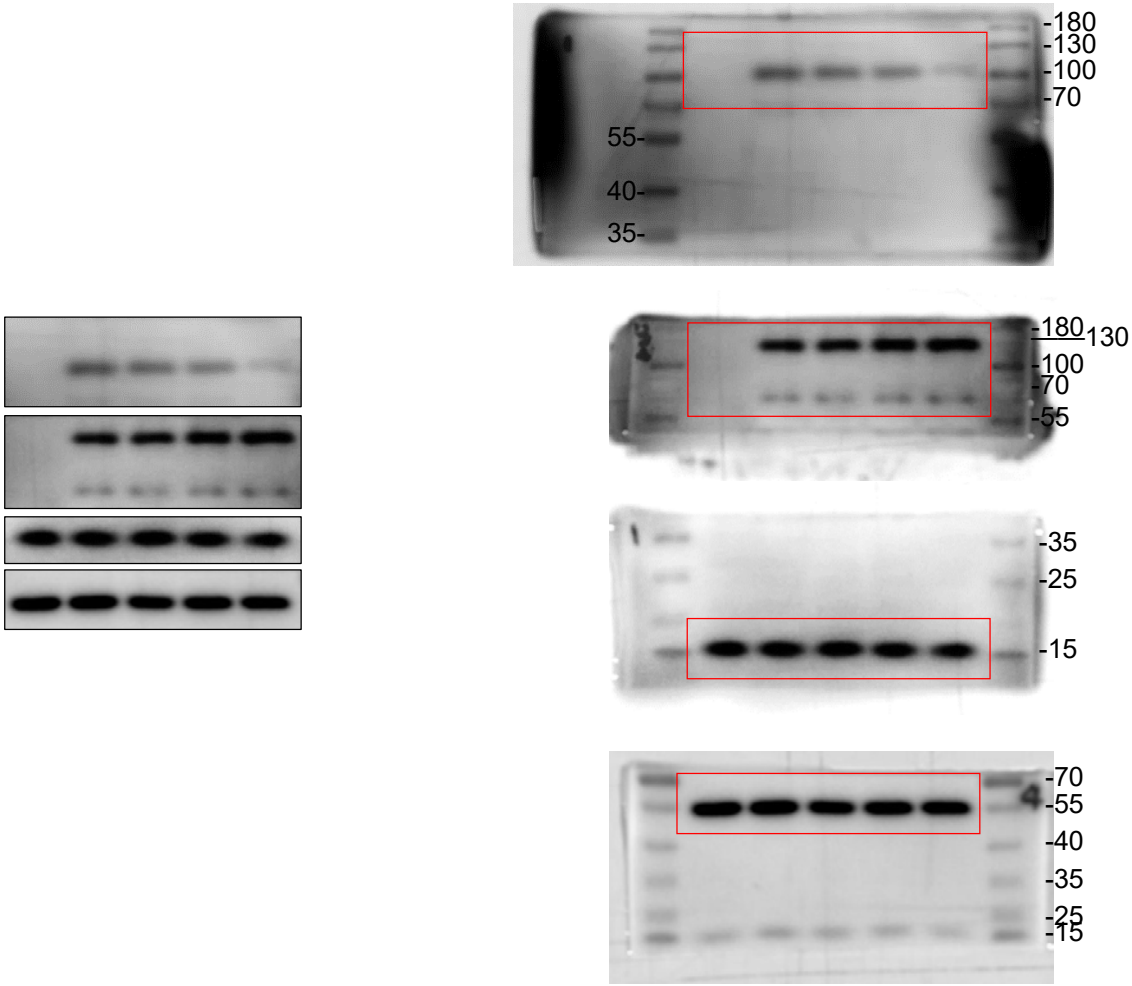

Fig.2M

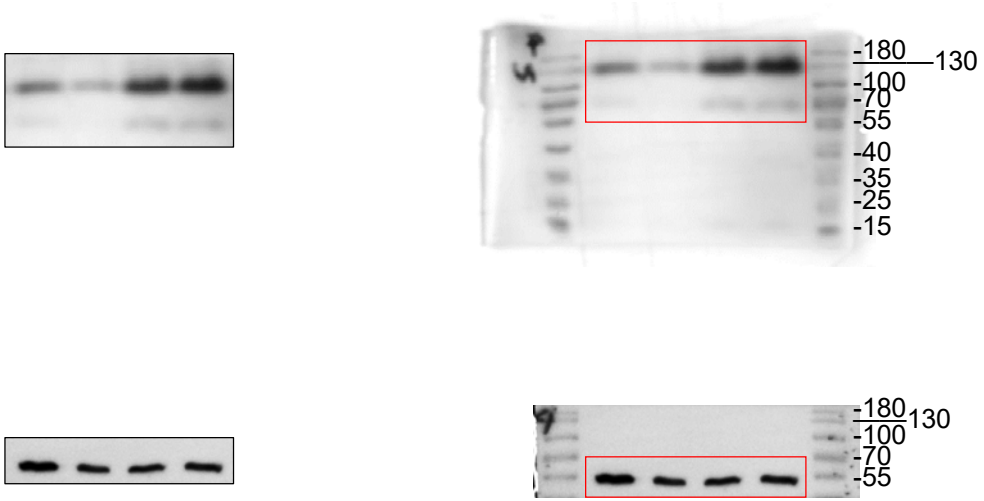

Fig.3C

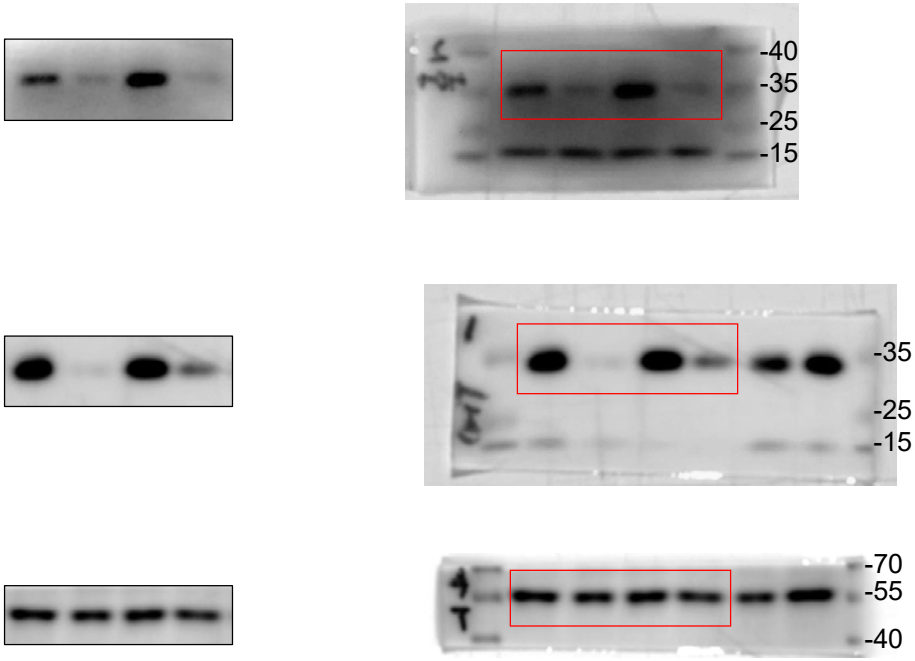

Fig.4B

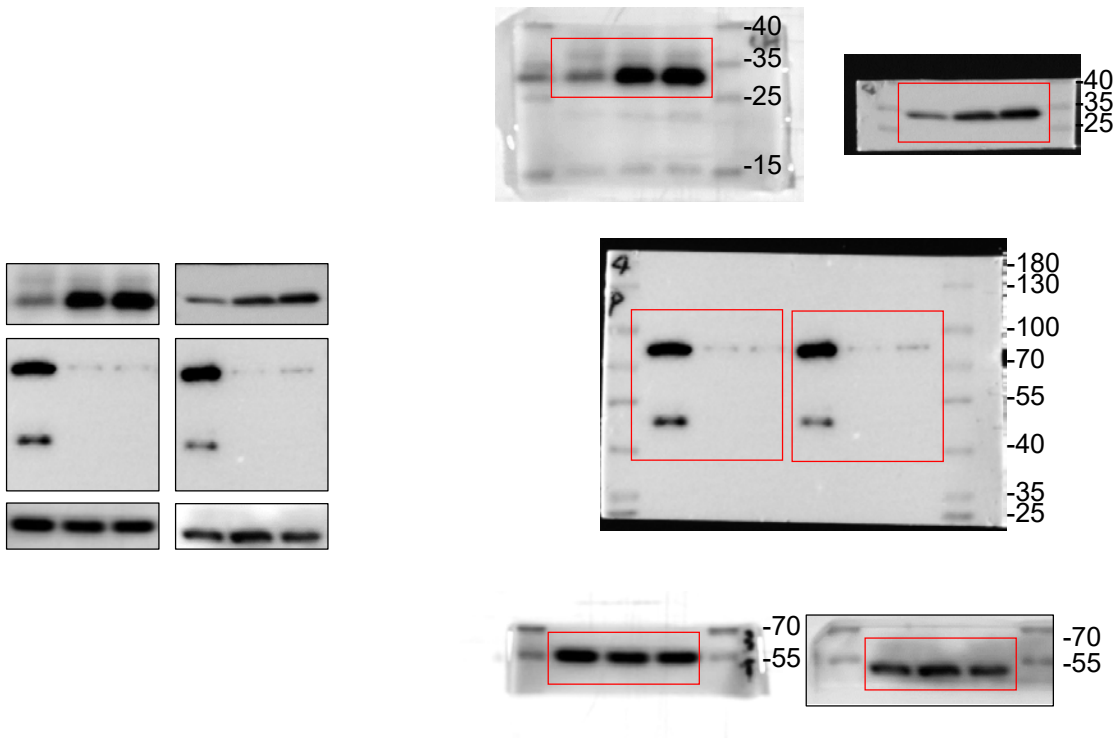

Fig.4C

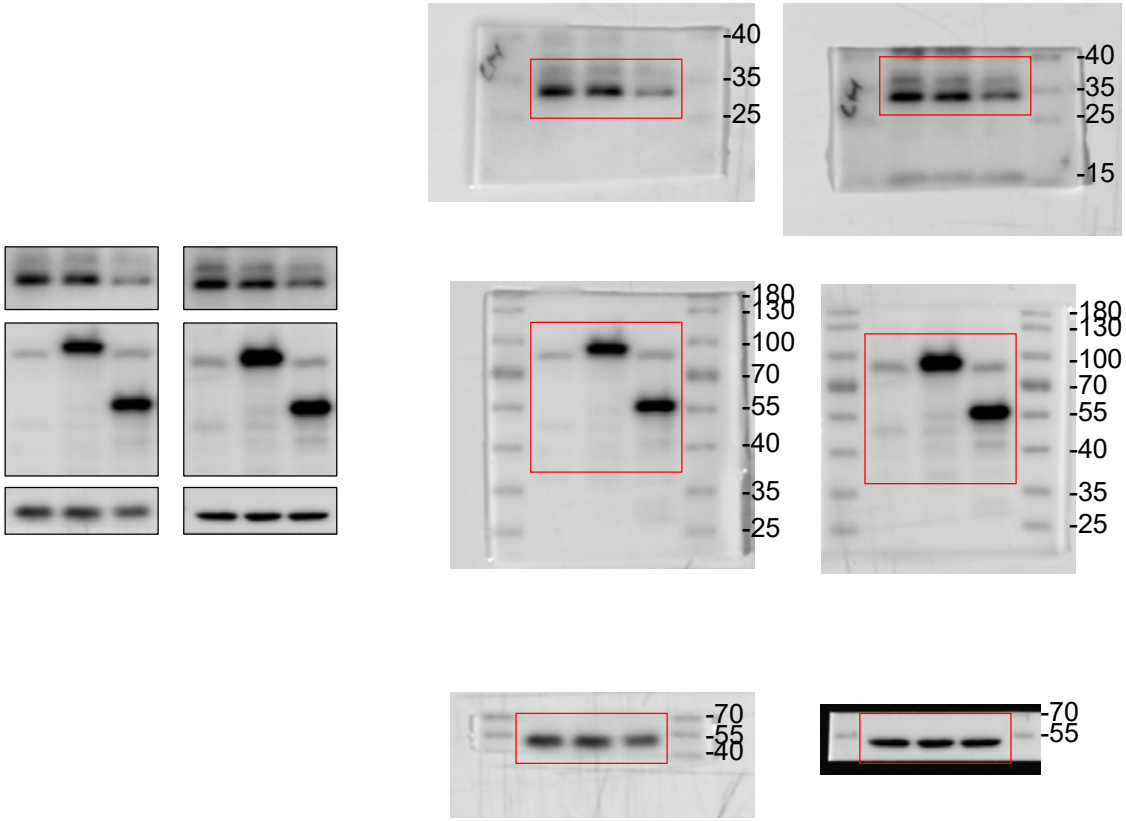
-40
-35
-25
-40
-35
-25
-15
-180
-130
-100
-70
-55
-40
-35
-25
-180
-130
-100
-70
-55
-40
-35
-25
-70
-55
-40
-70
-55

Fig.4D

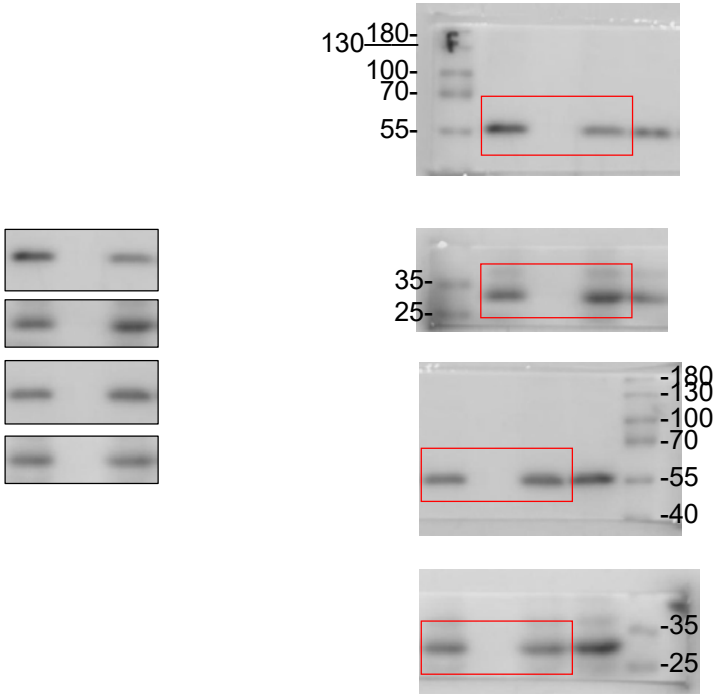

Fig.4E

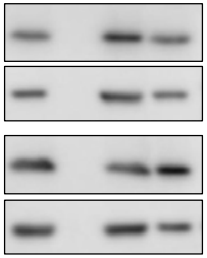

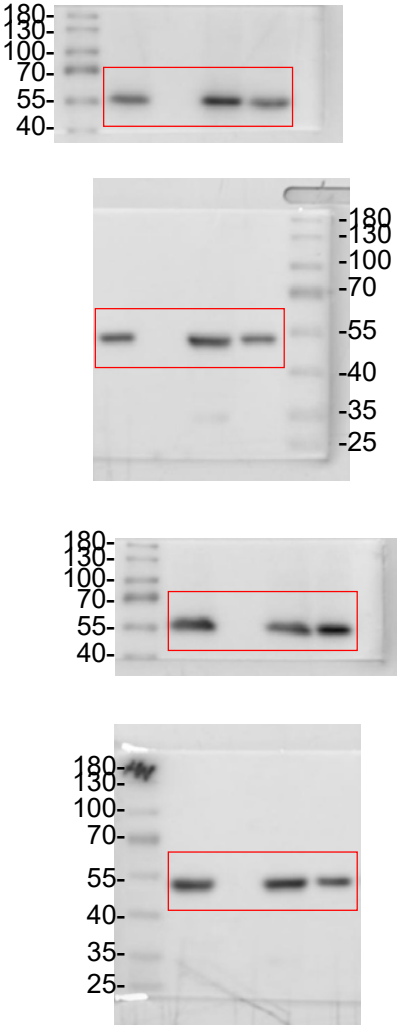
180-
130-
100-
70-
55-
40-
-180
-130
-100
-70
-55
-40
-35
-25
180-
130-
100-
70-
55-
40-
180-
130-
100-
70-
55-
40-
35-
25-

Fig.4F

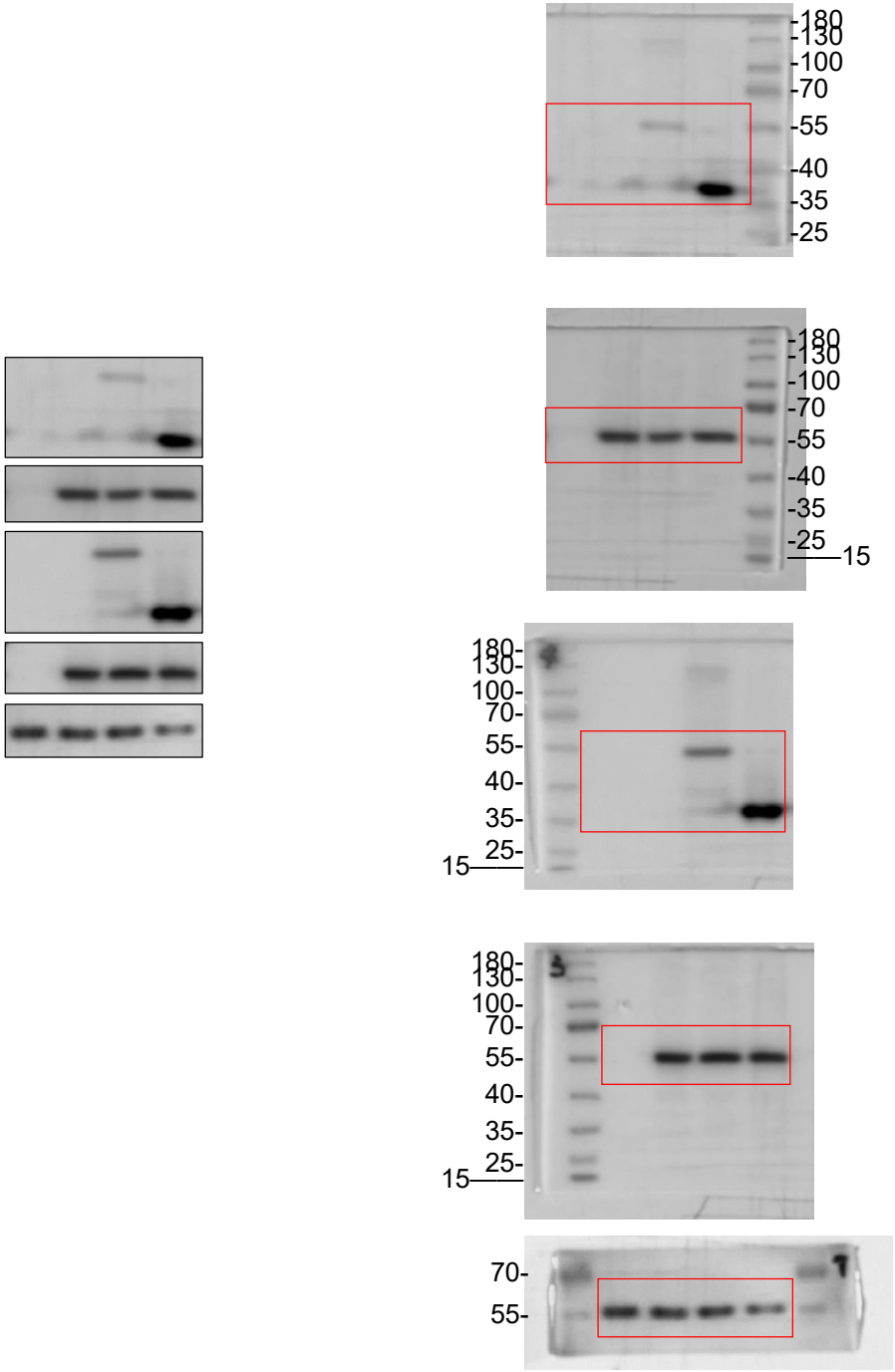

Fig.4G

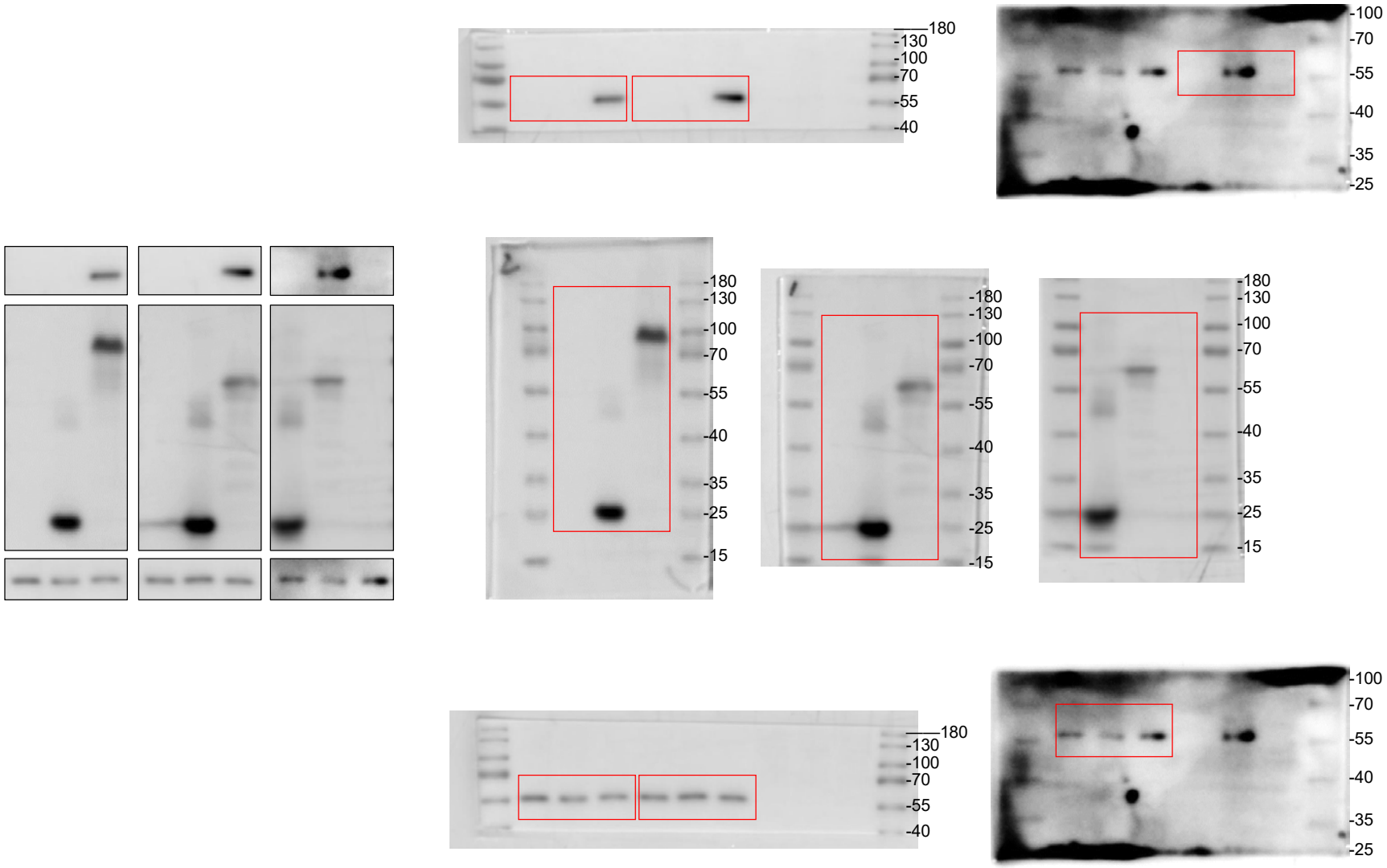

180
130
100
70
55
40
100
70
55
40
35
25
180
130
100
70
55
40
35
25
15
180
130
100
70
55
40
35
25
15
180
130
100
70
55
40
35
25
15
180
130
100
70
55
40
100
70
55
40
35
25

Fig.4J

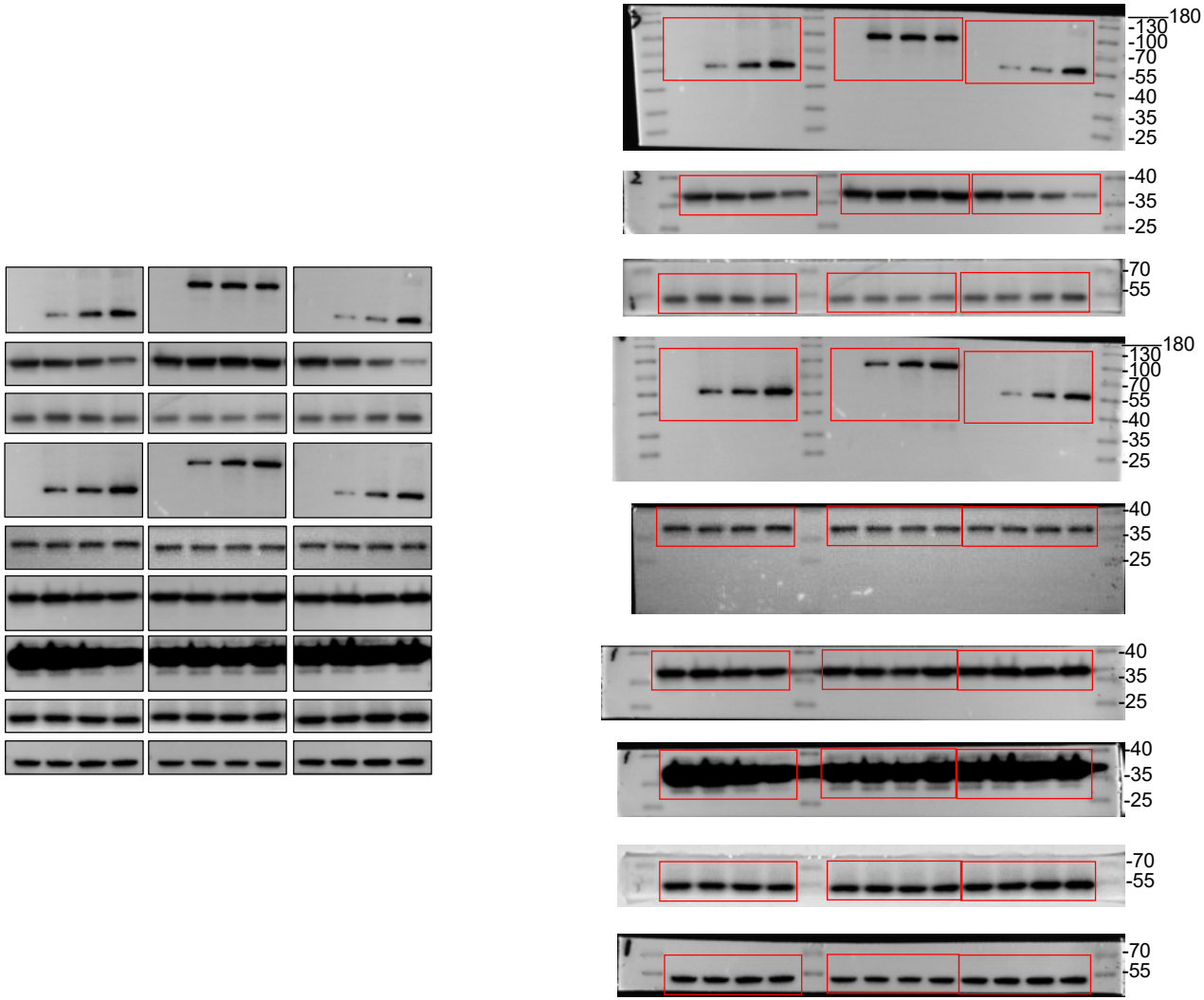

Fig.4K

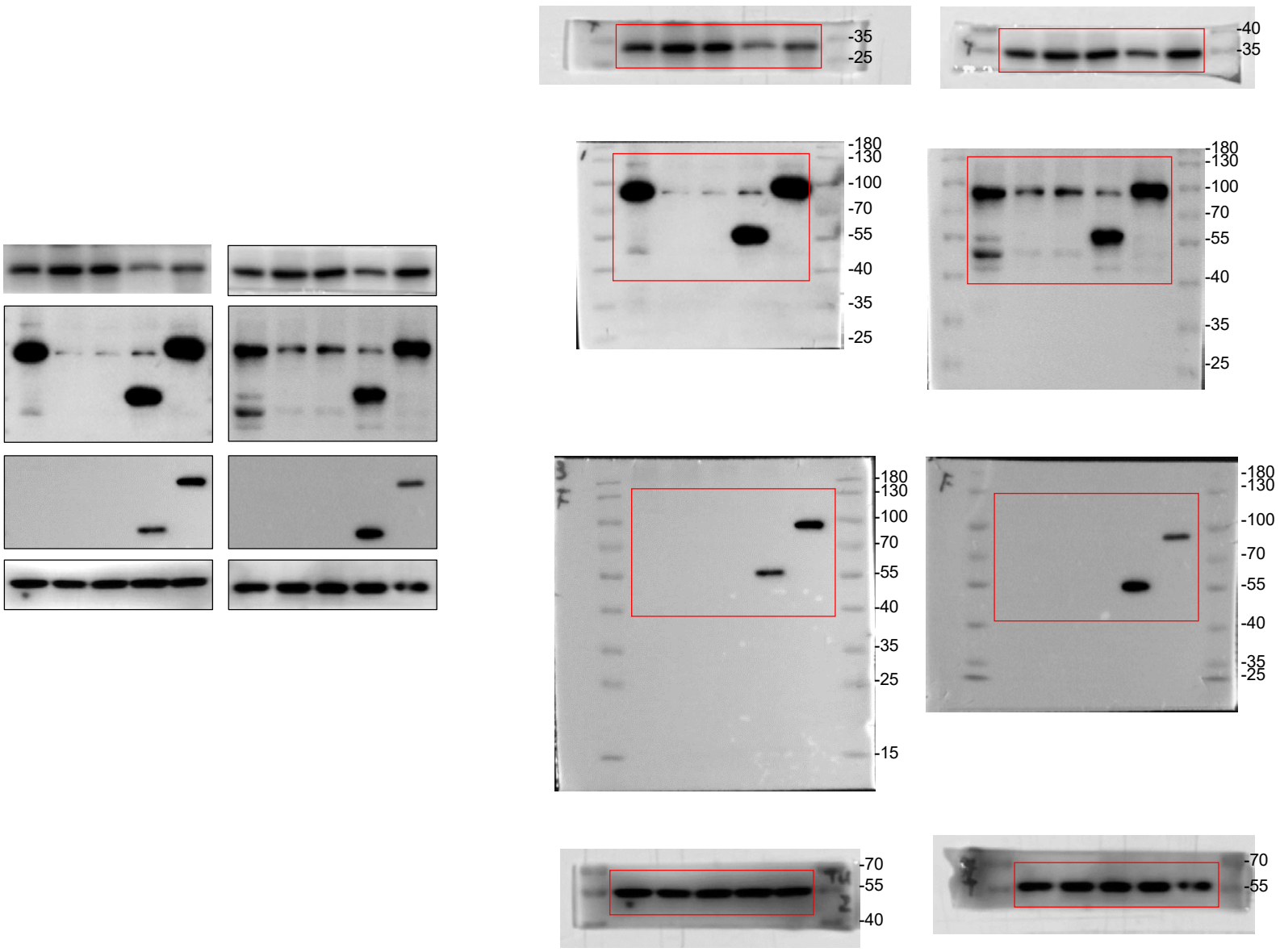
-35
-25
-40
-35
-180
-130
-100
-70
-55
-40
-35
-25
-180
-130
-100
-70
-55
-40
-35
-25
-180
-130
-100
-70
-55
-40
-35
-25
-15
-180
-130
-100
-70
-55
-40
-35
-25
-70
-55
-40
-70
-55

Fig.5A

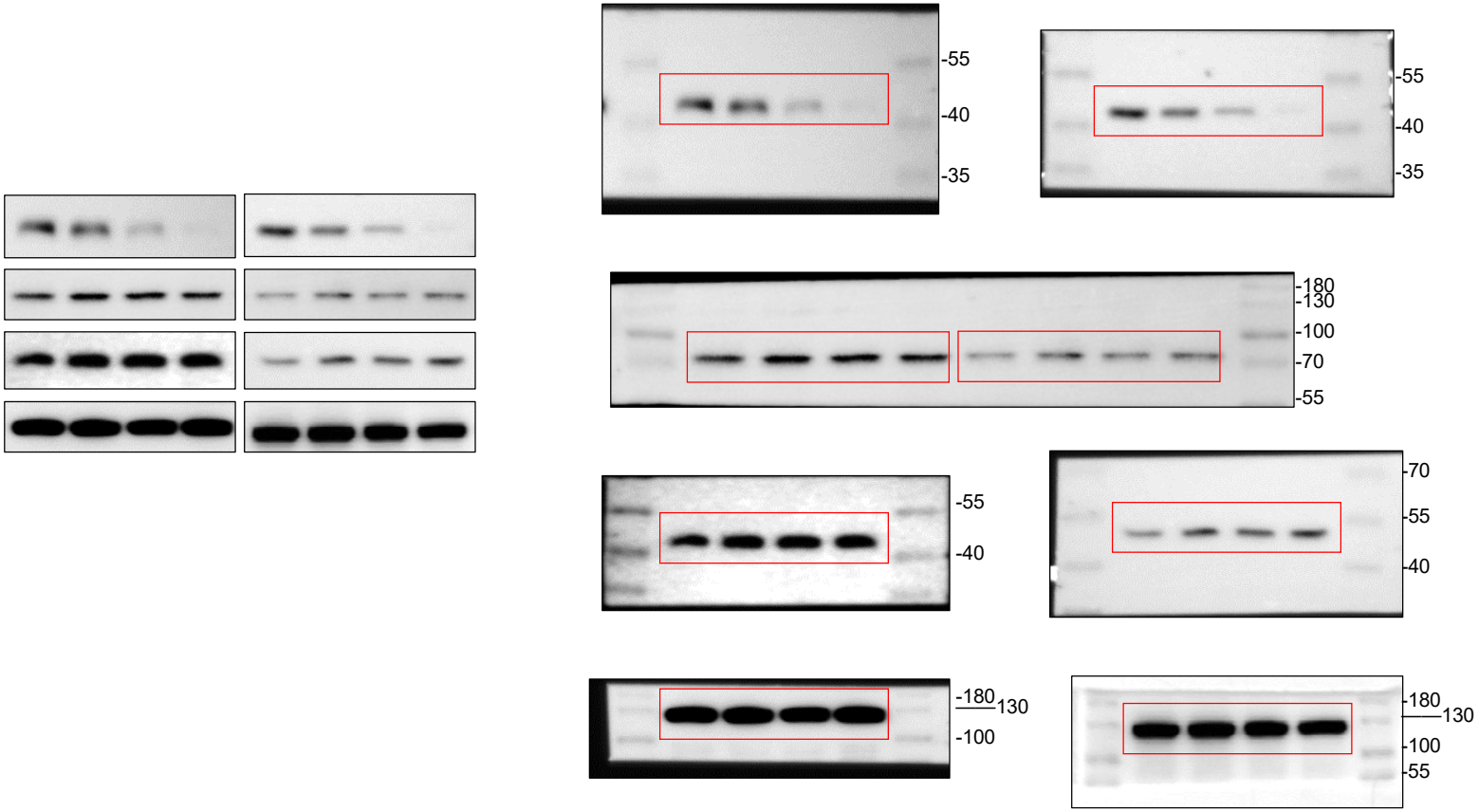
-55
-40
-35
-55
-40
-35
-180
-130
-100
-70
-55
-55
-40
-70
-55
-40
-180
130
-100
-180
130
-100
-55

Fig.5B

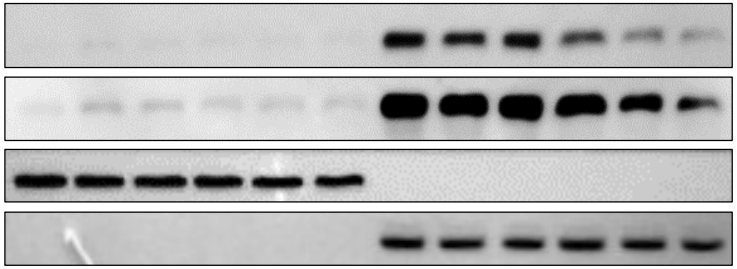

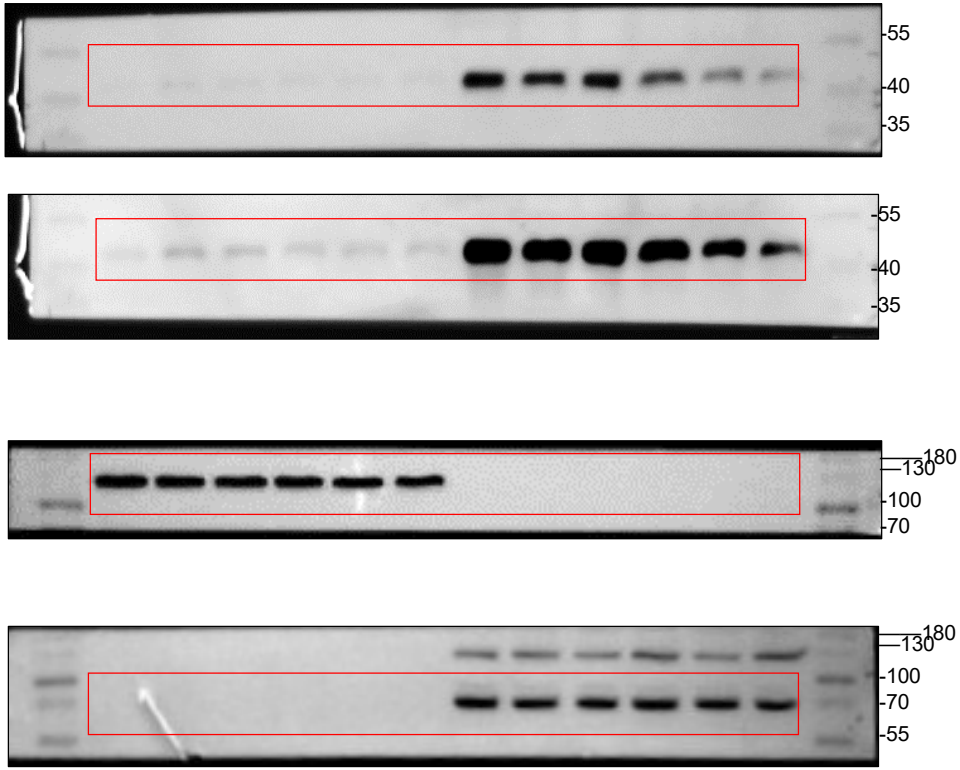

Fig.5C

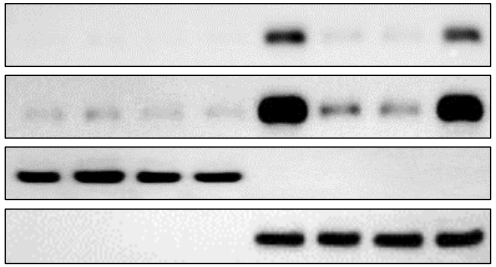

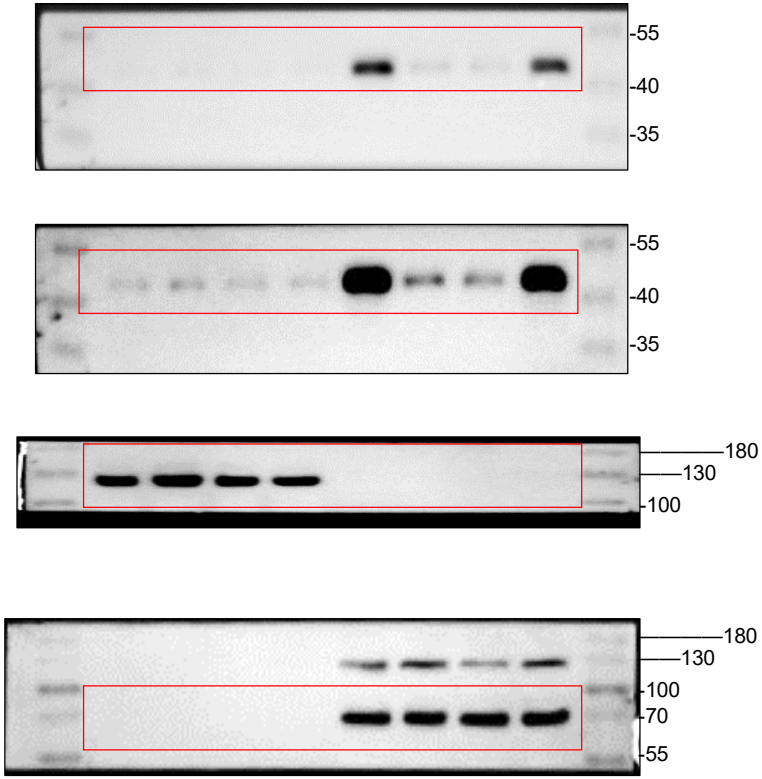

Fig.5D

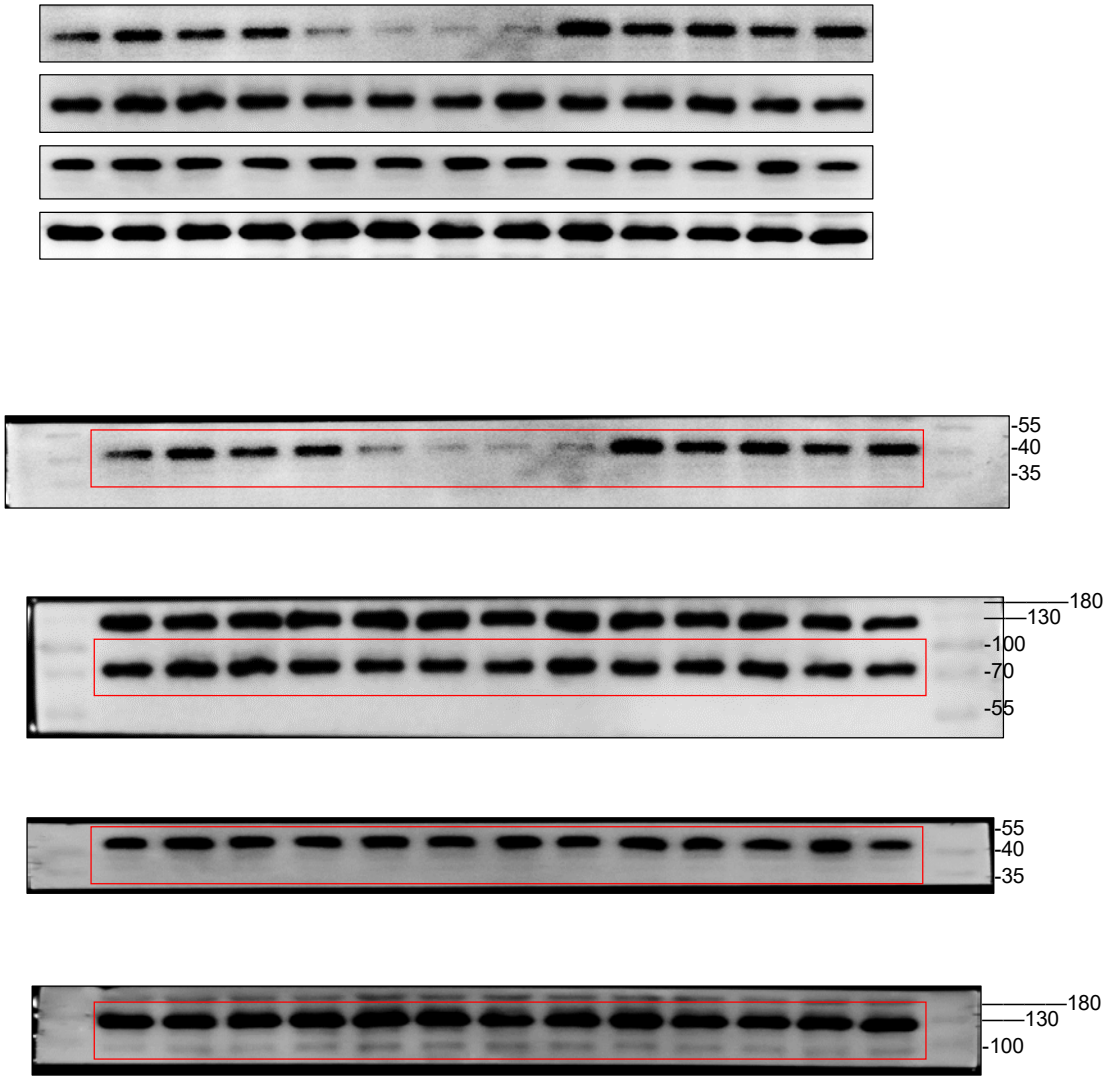

Fig.5E

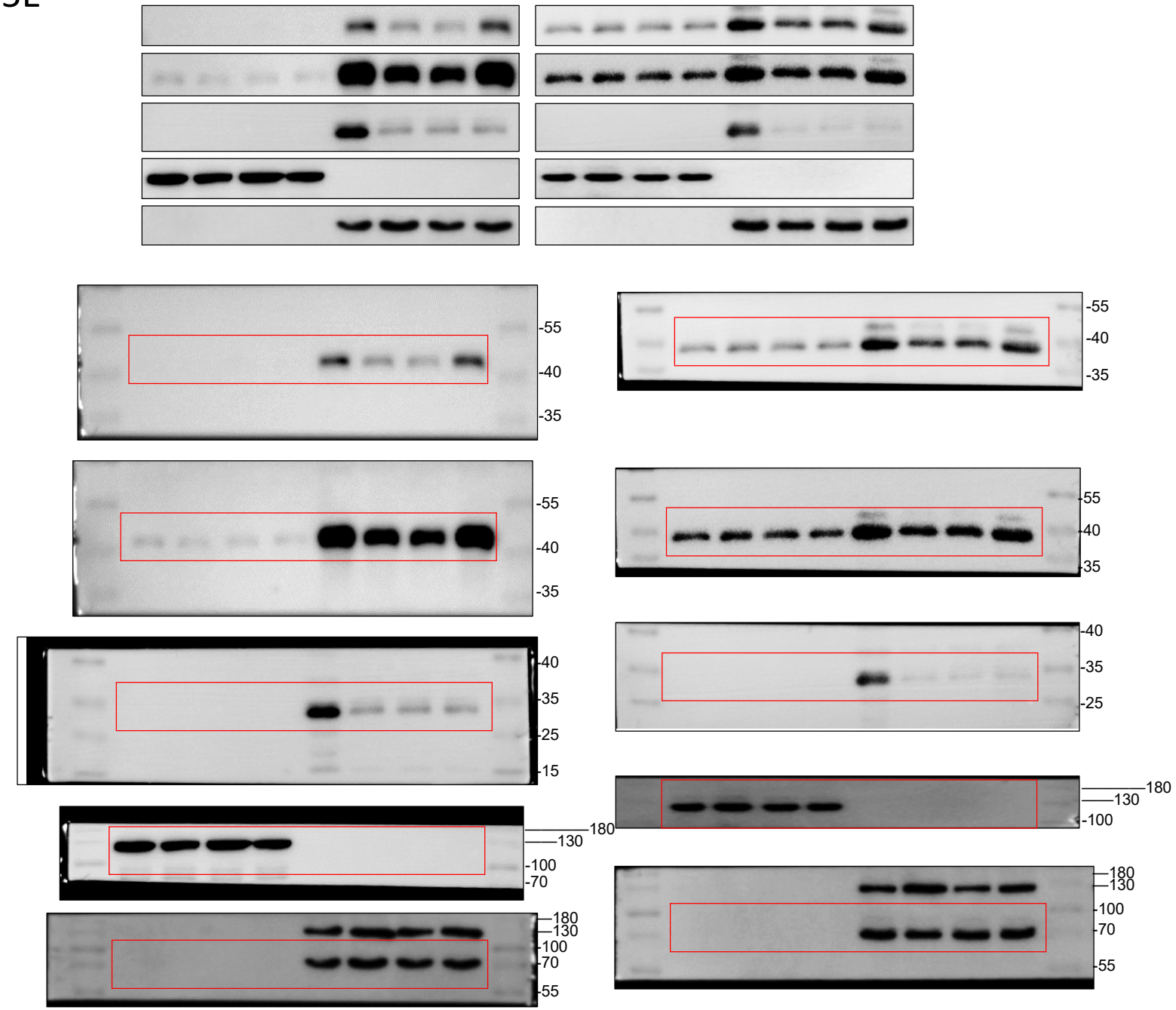
-55
-40
-35
-55
-40
-35
-55
-40
-35
-55
-40
-35
-40
-35
-25
-15
-40
-35
-25
180
130
-100
-70
180
130
-100
180
130
100
70
55
180
130
100
70
55

Fig.5G

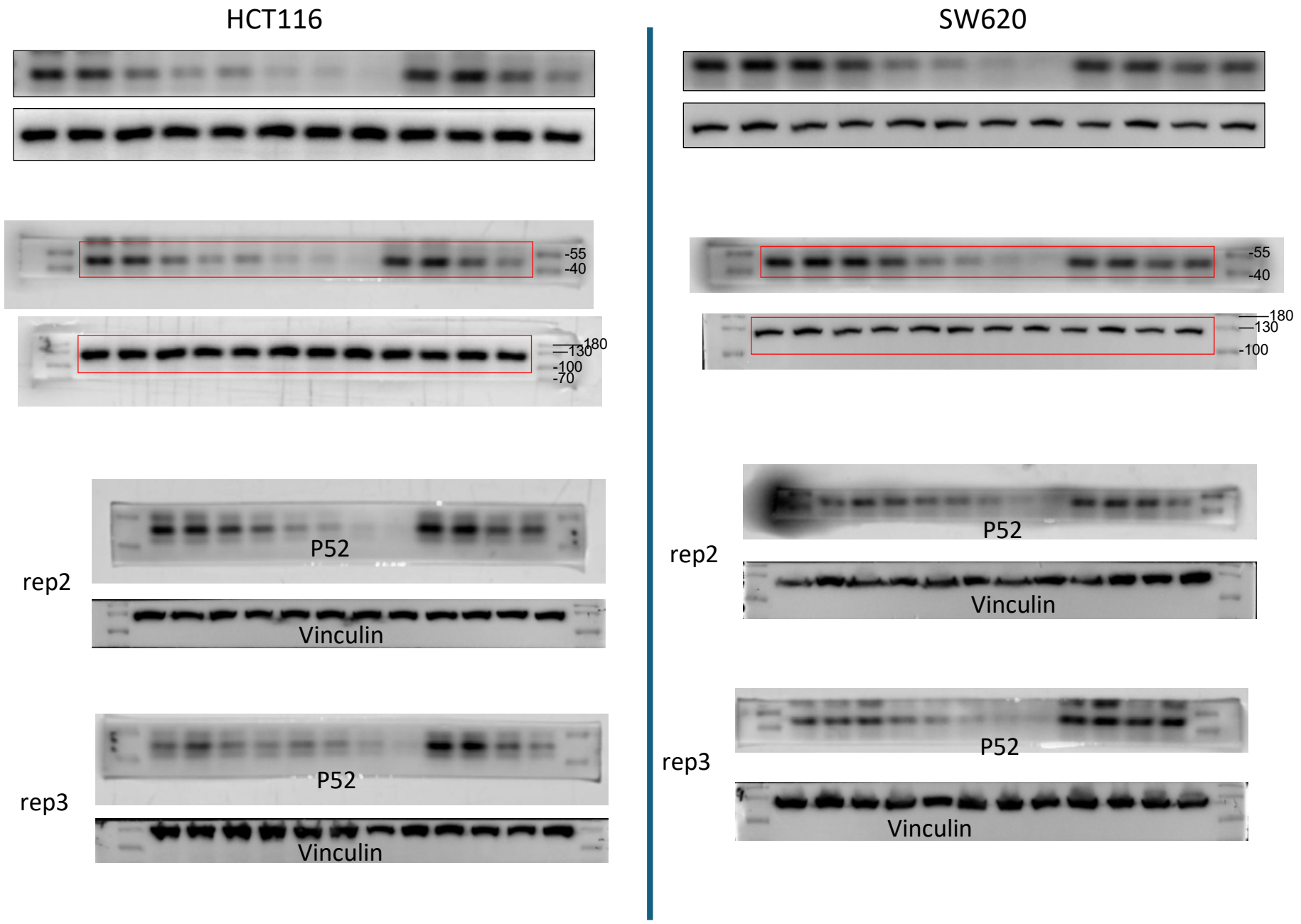
HCT116
SW620
-55
-40
180
130
-100
-70
-55
-40
180
130
-100
rep2
P52
Vinculin
rep3
P52
Vinculin
rep2
P52
Vinculin
rep3
P52
Vinculin

Fig.5H

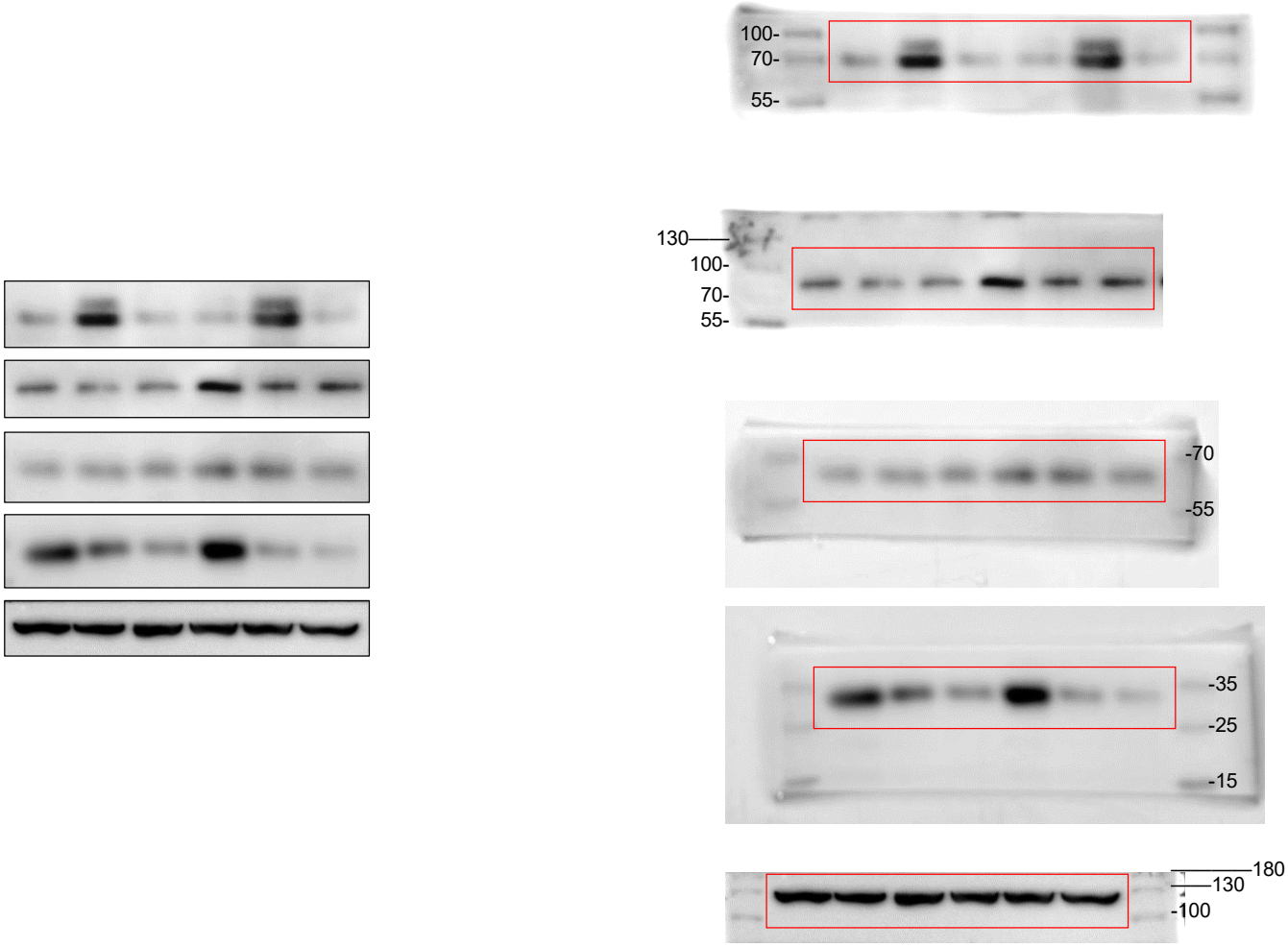

Fig.5I

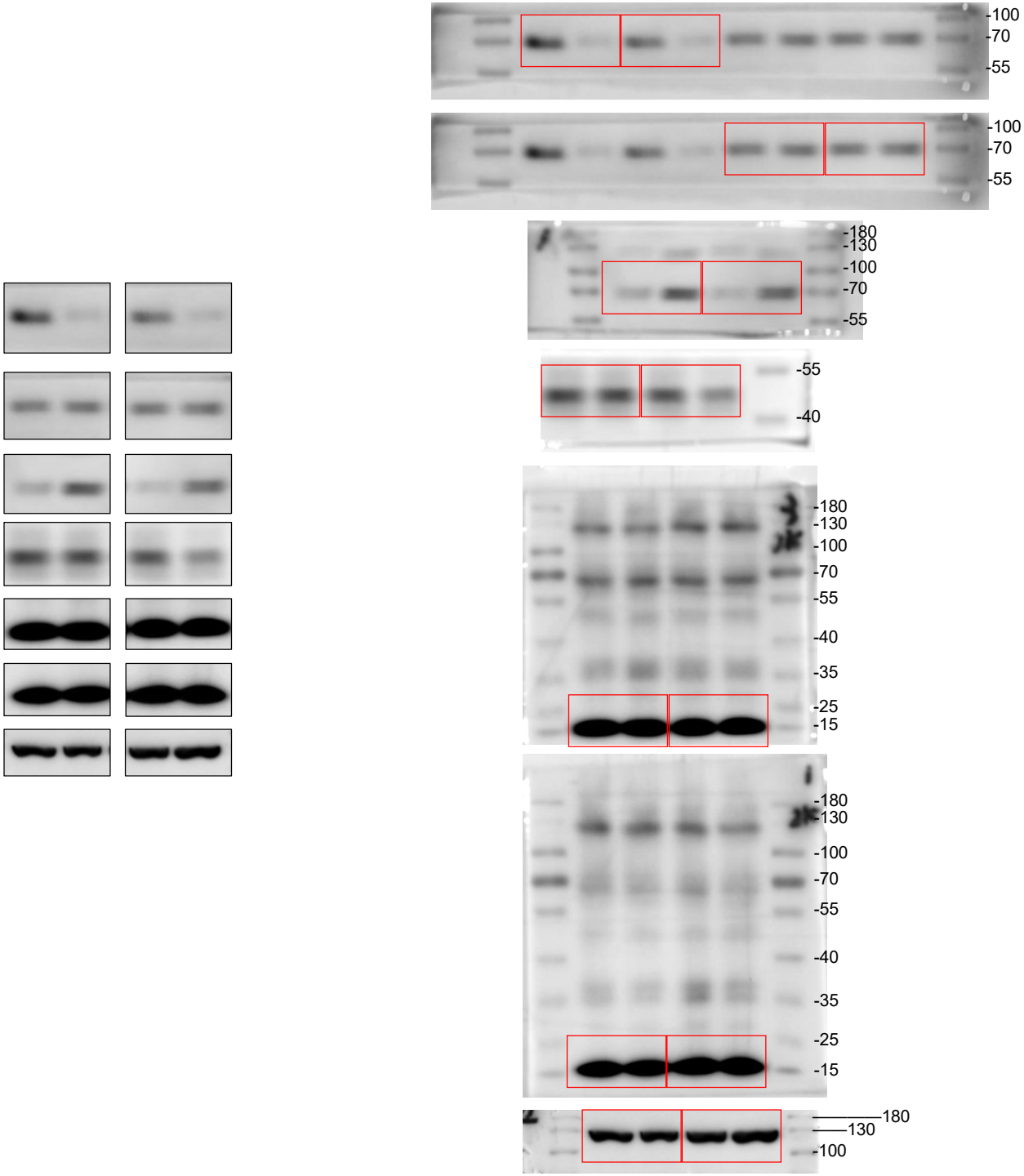
-100
-70
-55
-100
-70
-55
-180
-130
-100
-70
-55
-55
-40
-180
-130
-100
-70
-55
-40
-35
-25
-15
-180
-130
-100
-70
-55
-40
-35
-25
-15
180
130
-100

Fig.5J

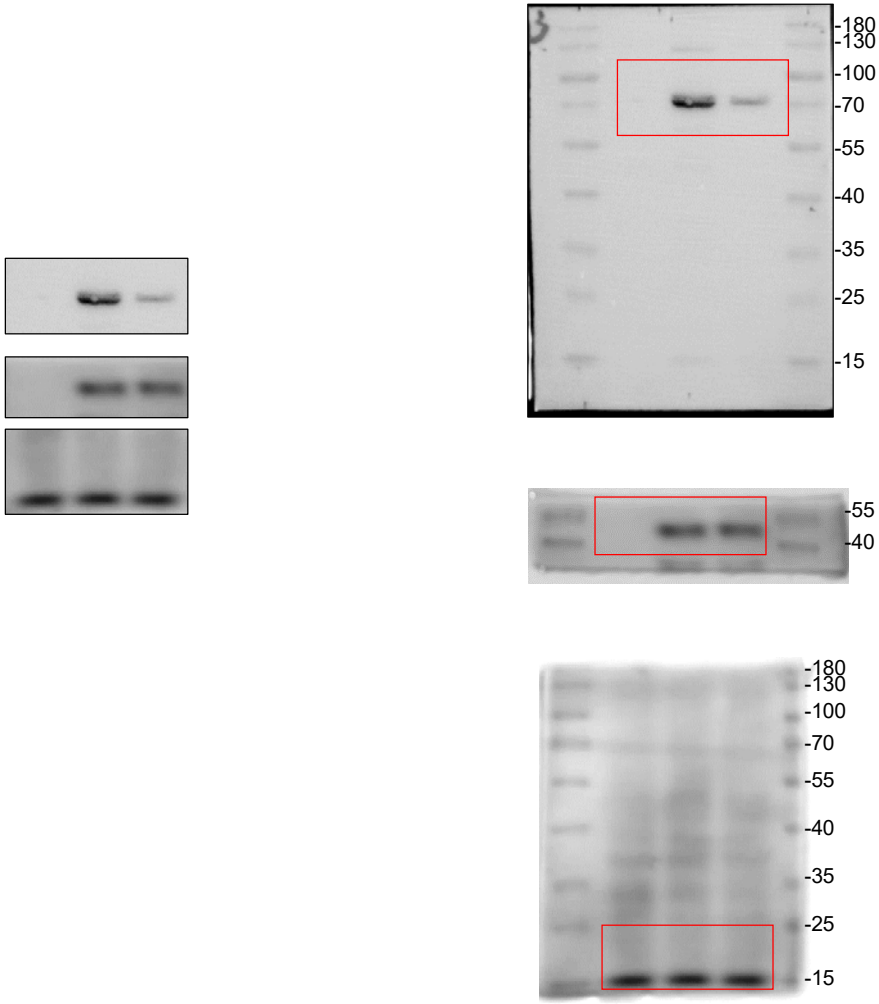

Fig.5K

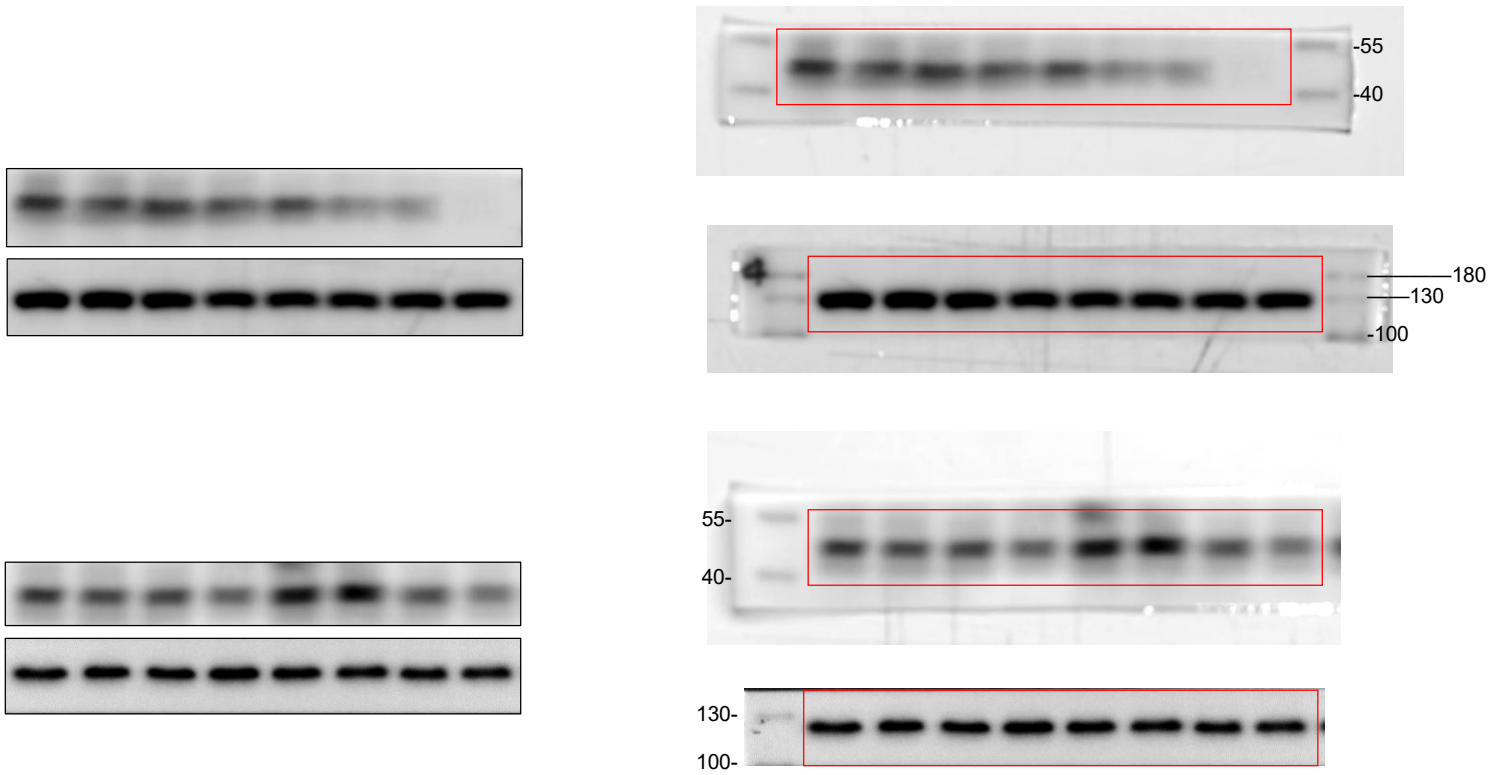

Fig.5L

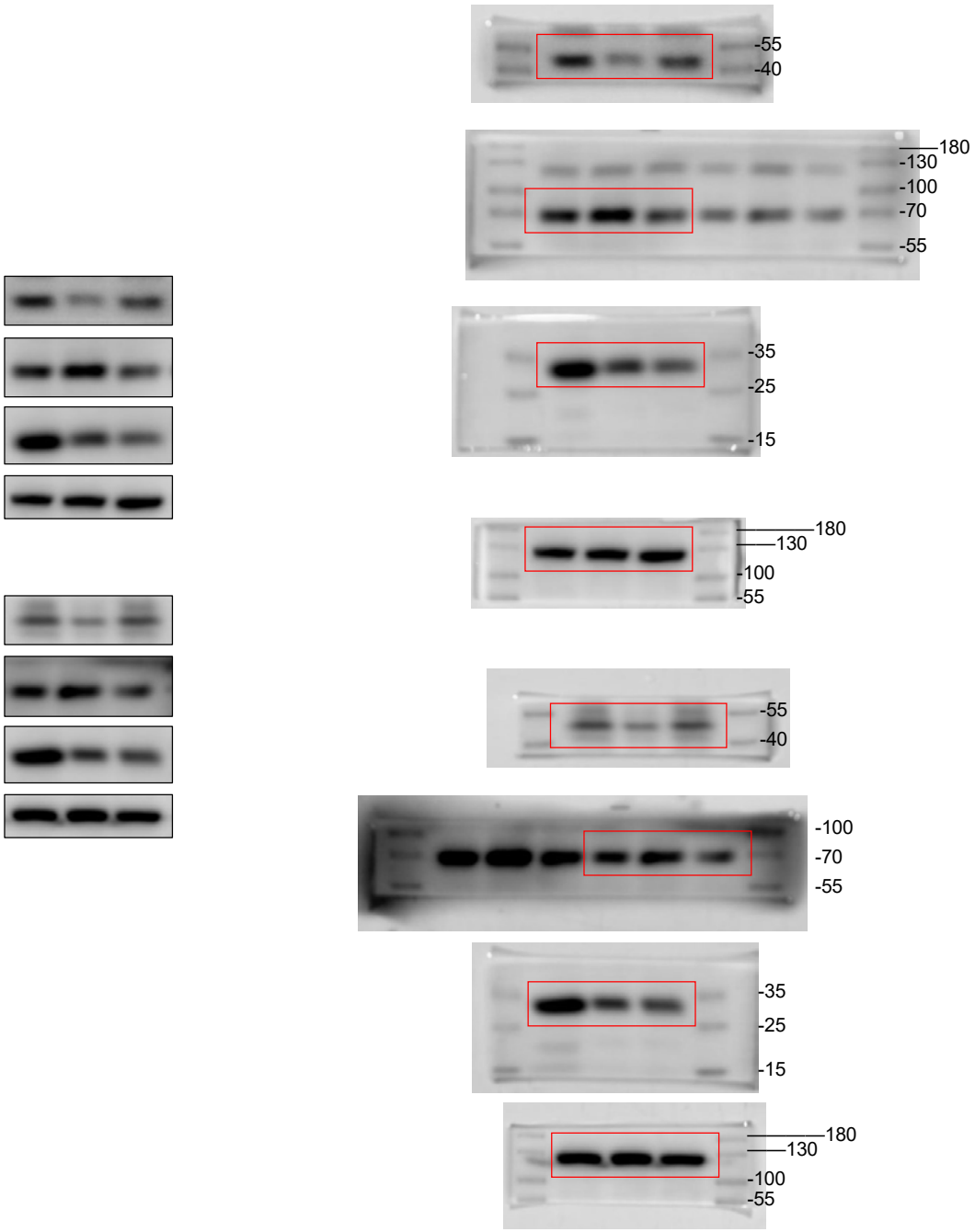

-55
-40
180
-130
-100
-70
-55
-35
-25
-15
180
130
-100
-55
-55
-40
-100
-70
-55
-35
-25
-15
180
130
-100
-55

Fig.6B

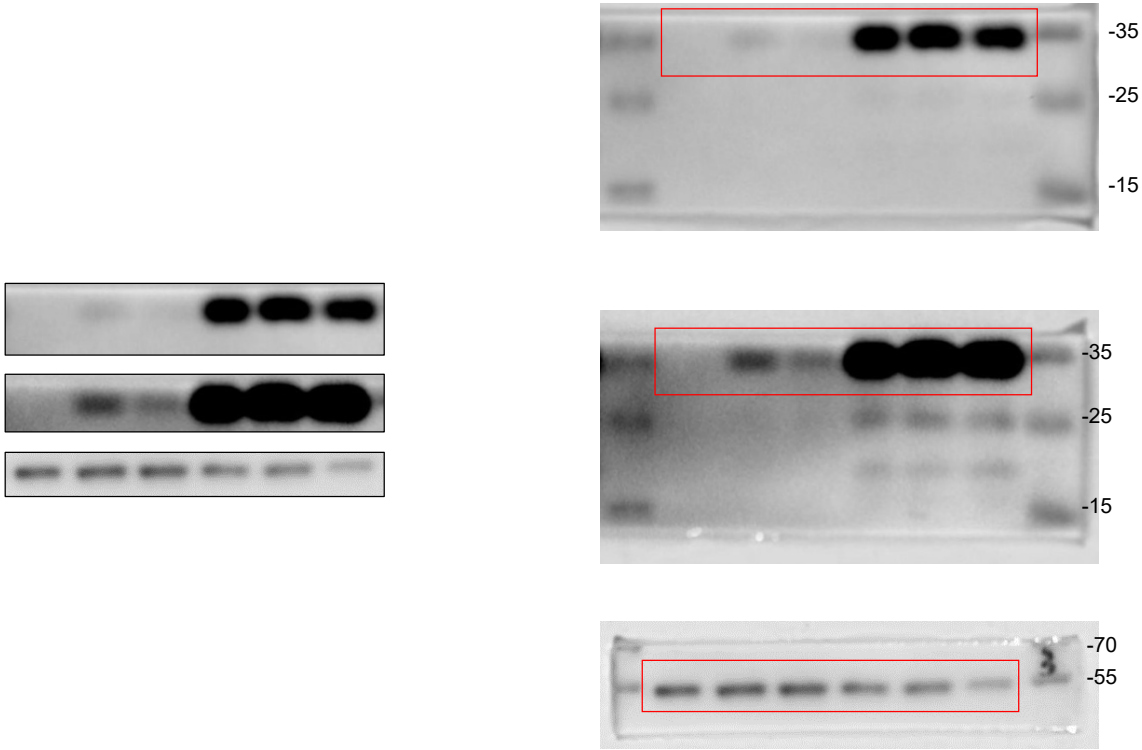

Fig.6D

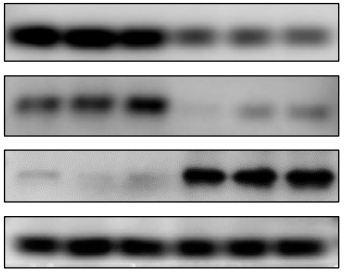

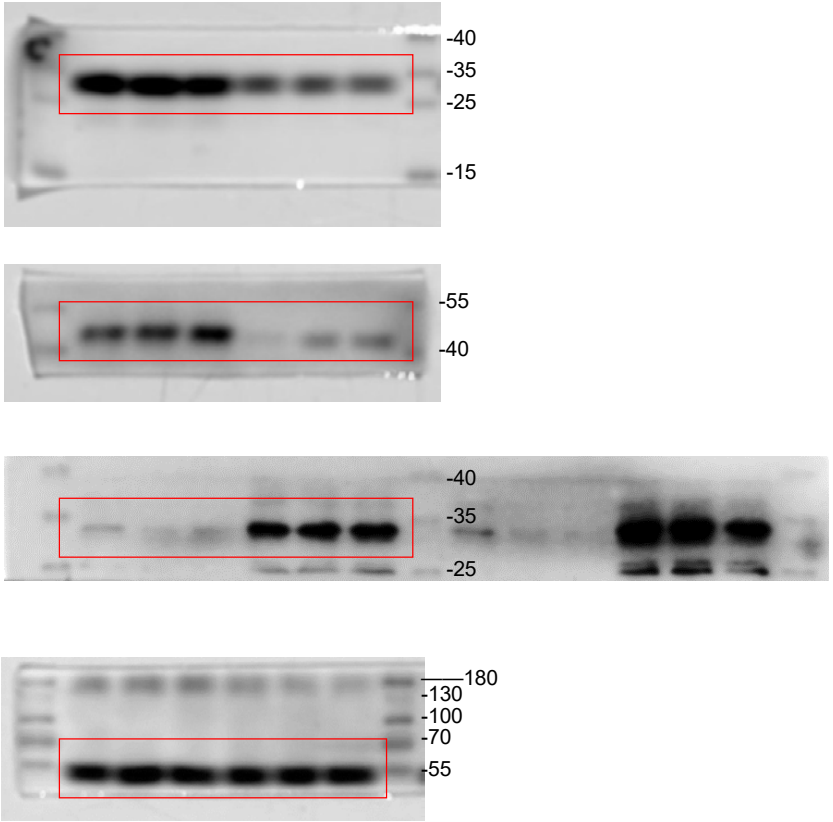

Fig.6E

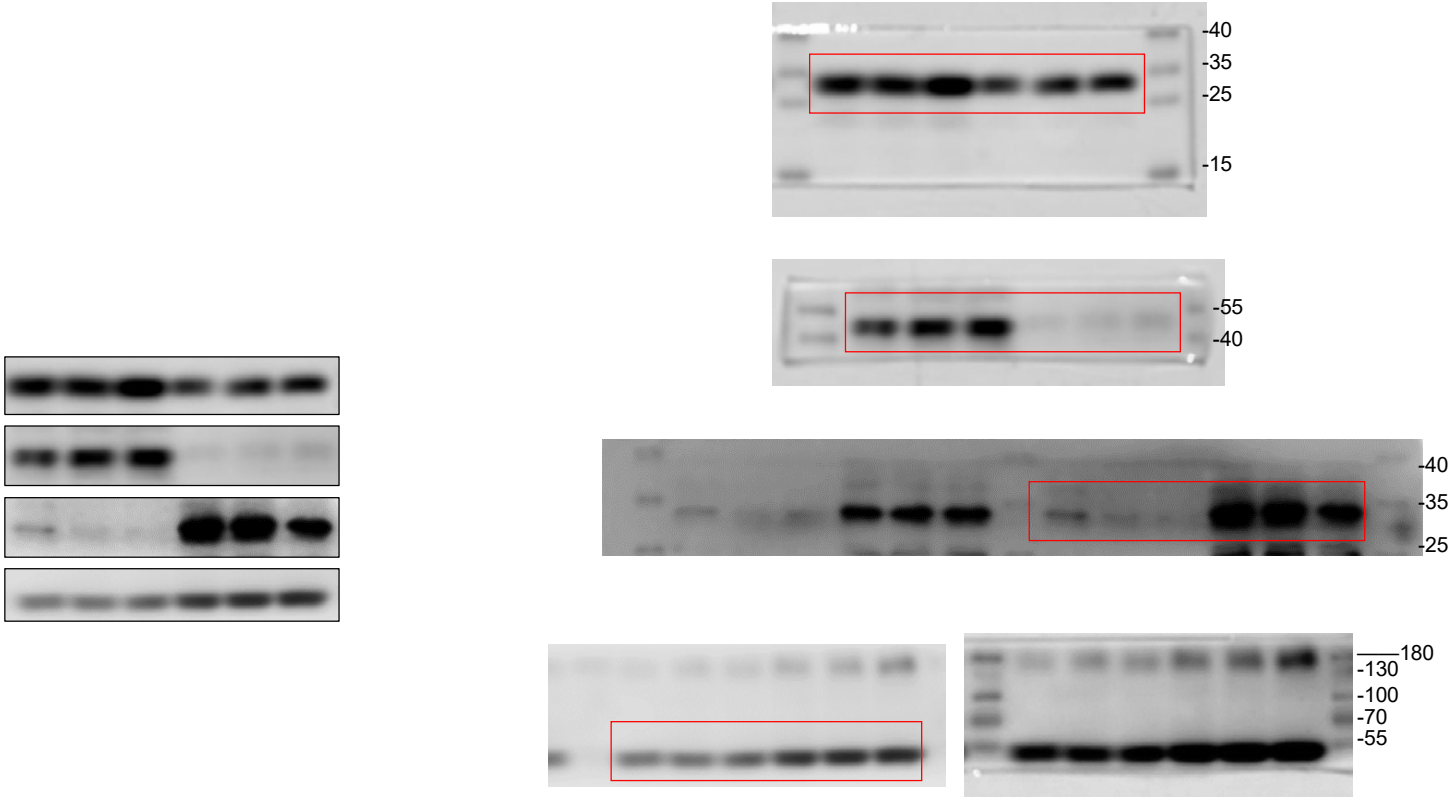

Fig.S1A

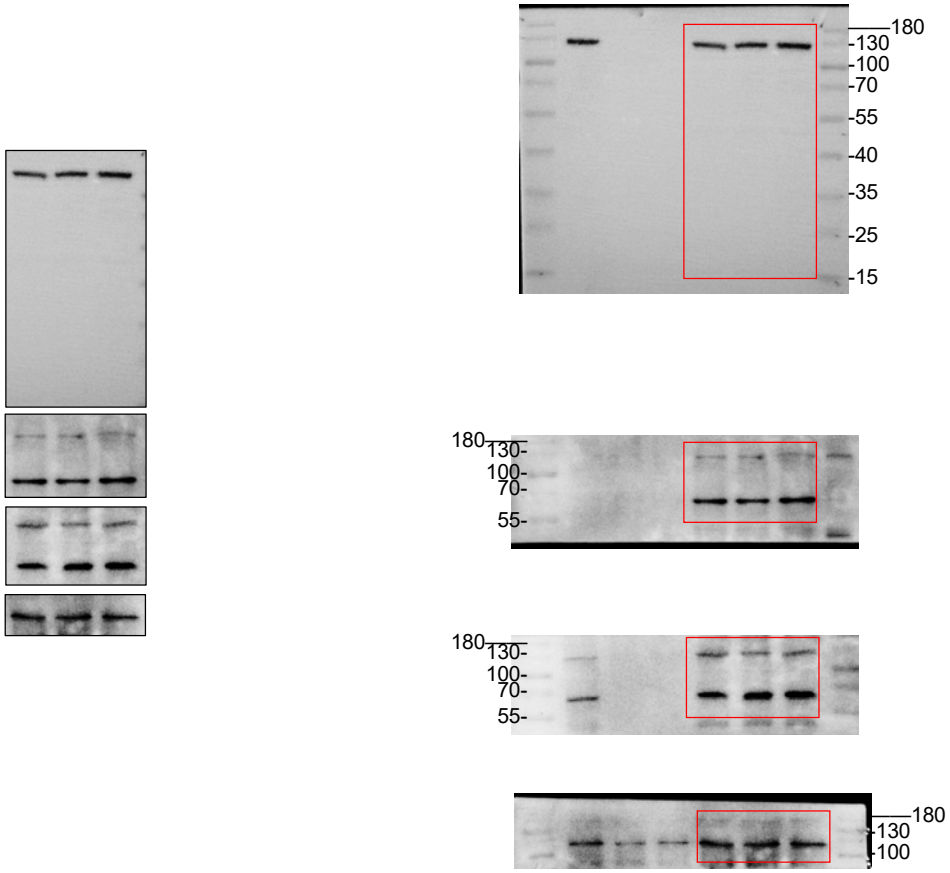

180
130
100
70
55
40
35
25
15
180
130
100
70
55
180
130
100
70
55
180
130
100

Fig.S1B

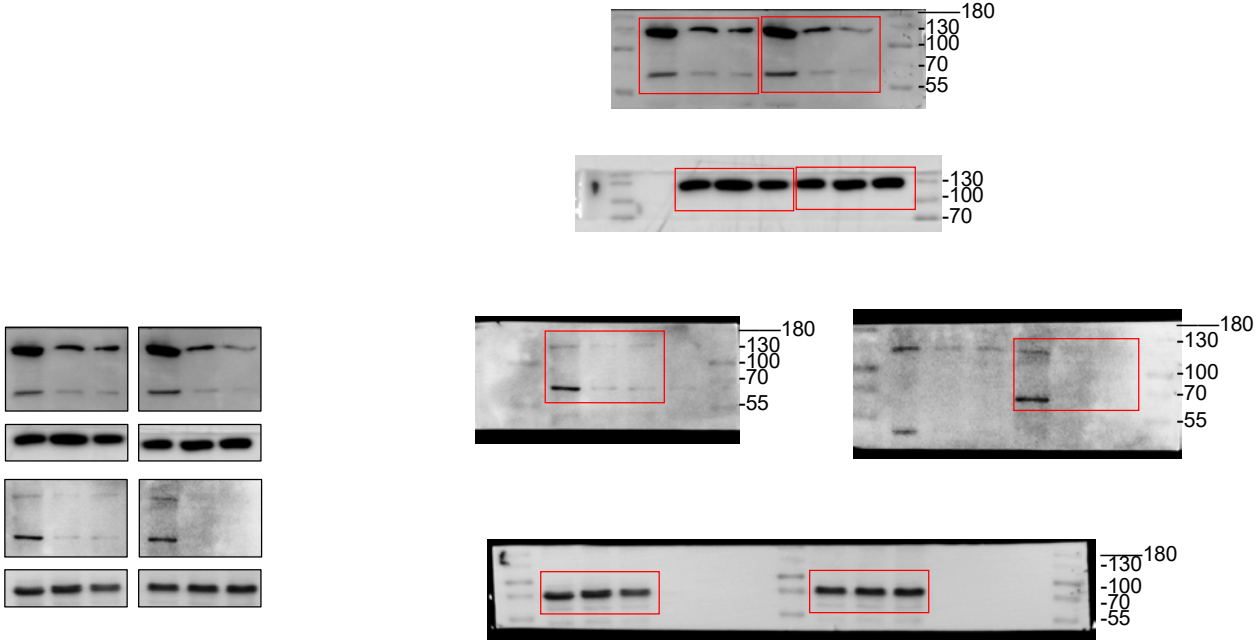
180
130
100
70
55
130
100
70
180
130
100
70
55
180
130
100
70
55
180
130
100
70
55

Fig.S1E

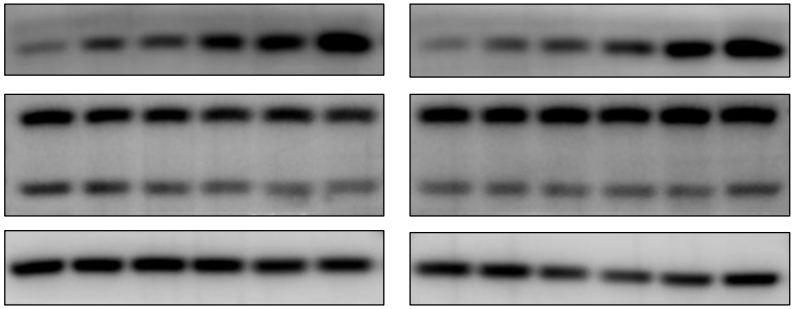

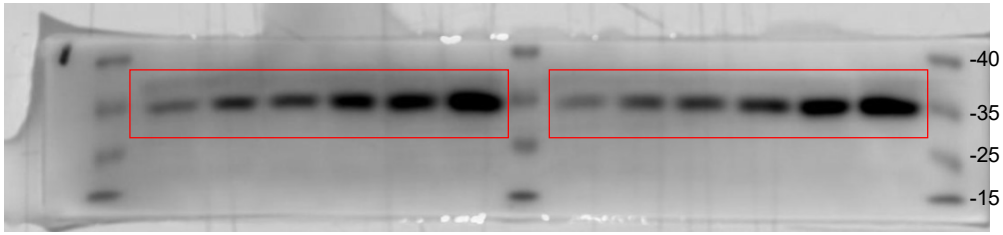

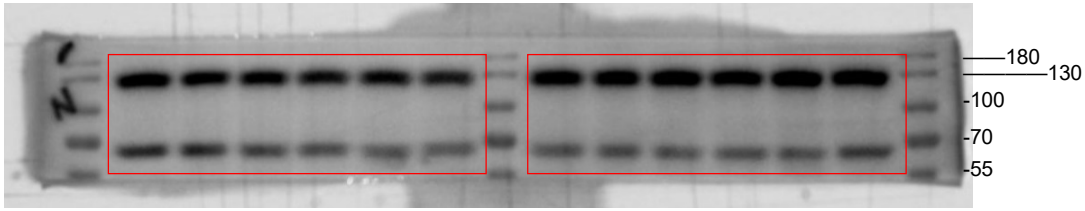

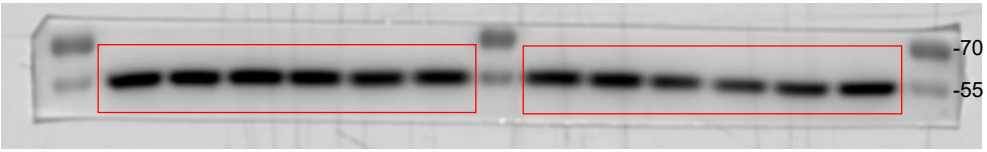

Fig.S1G

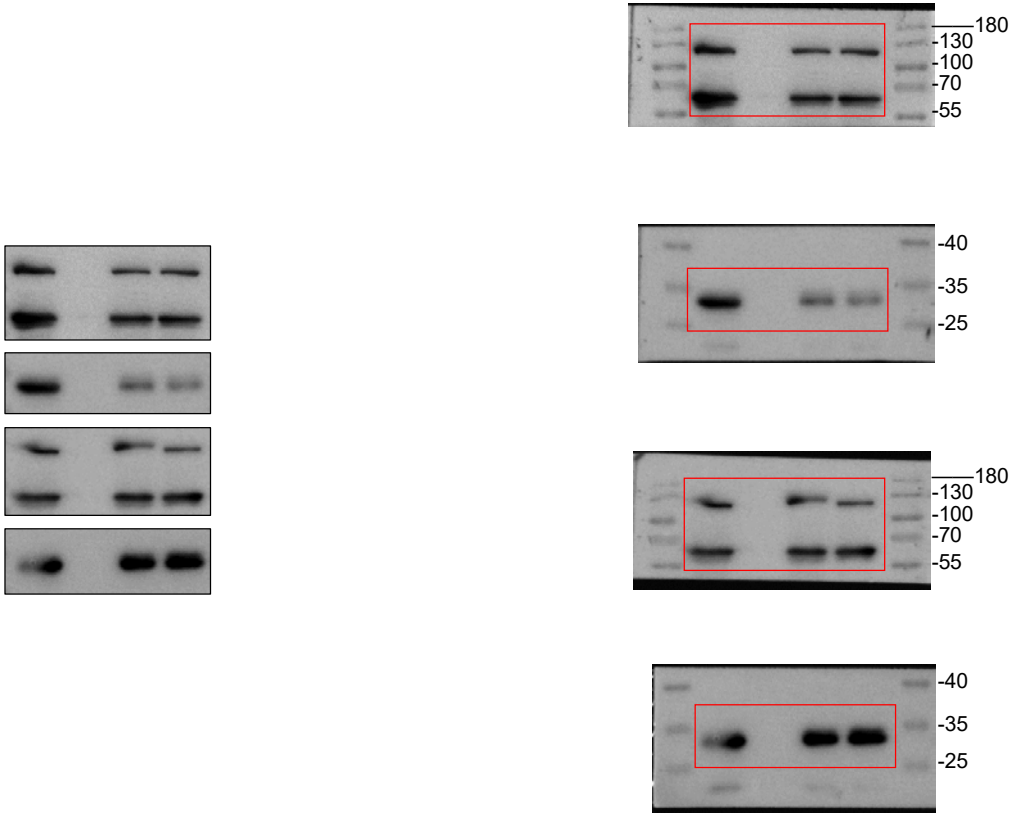

Fig.S1I

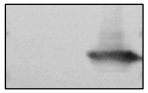

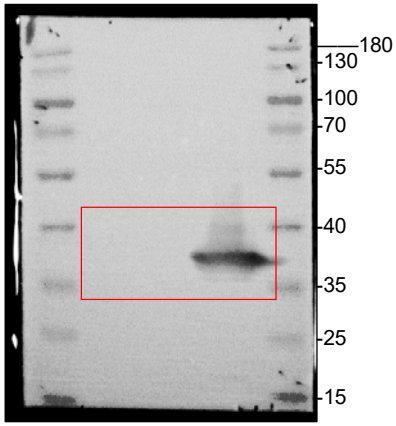

Fig.S2A

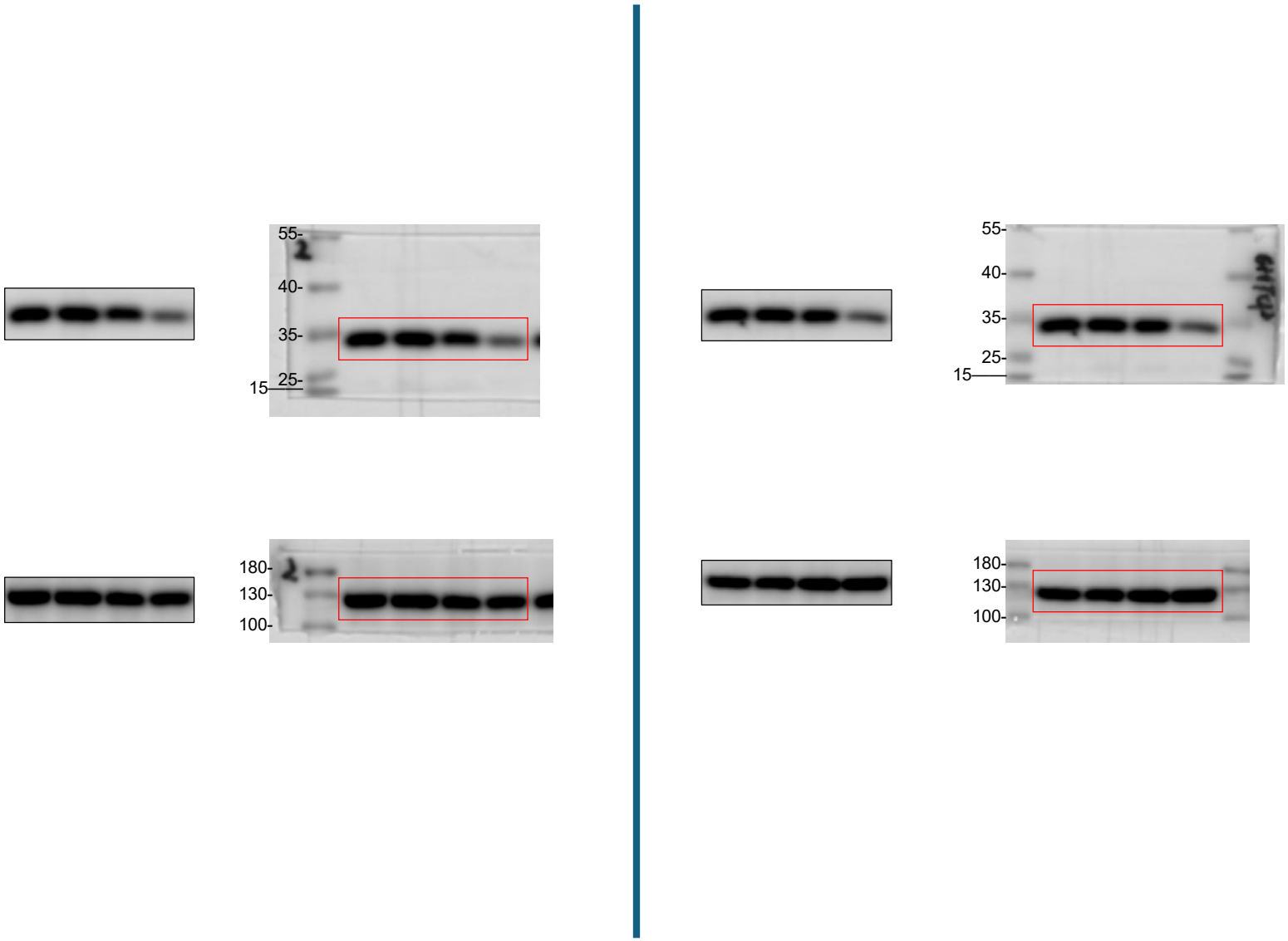

Fig.S2B

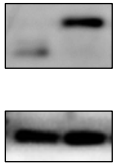

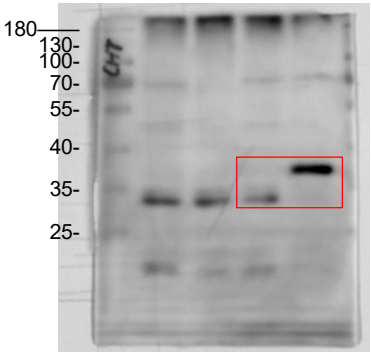

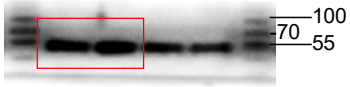

Fig.S2C

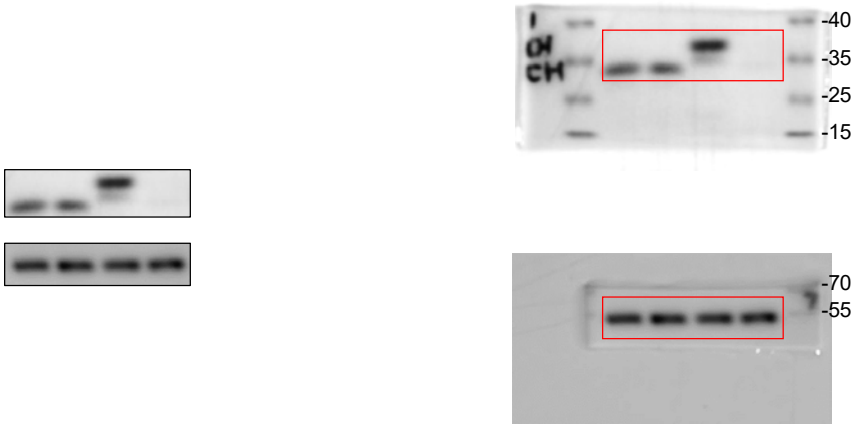

Fig.S2D

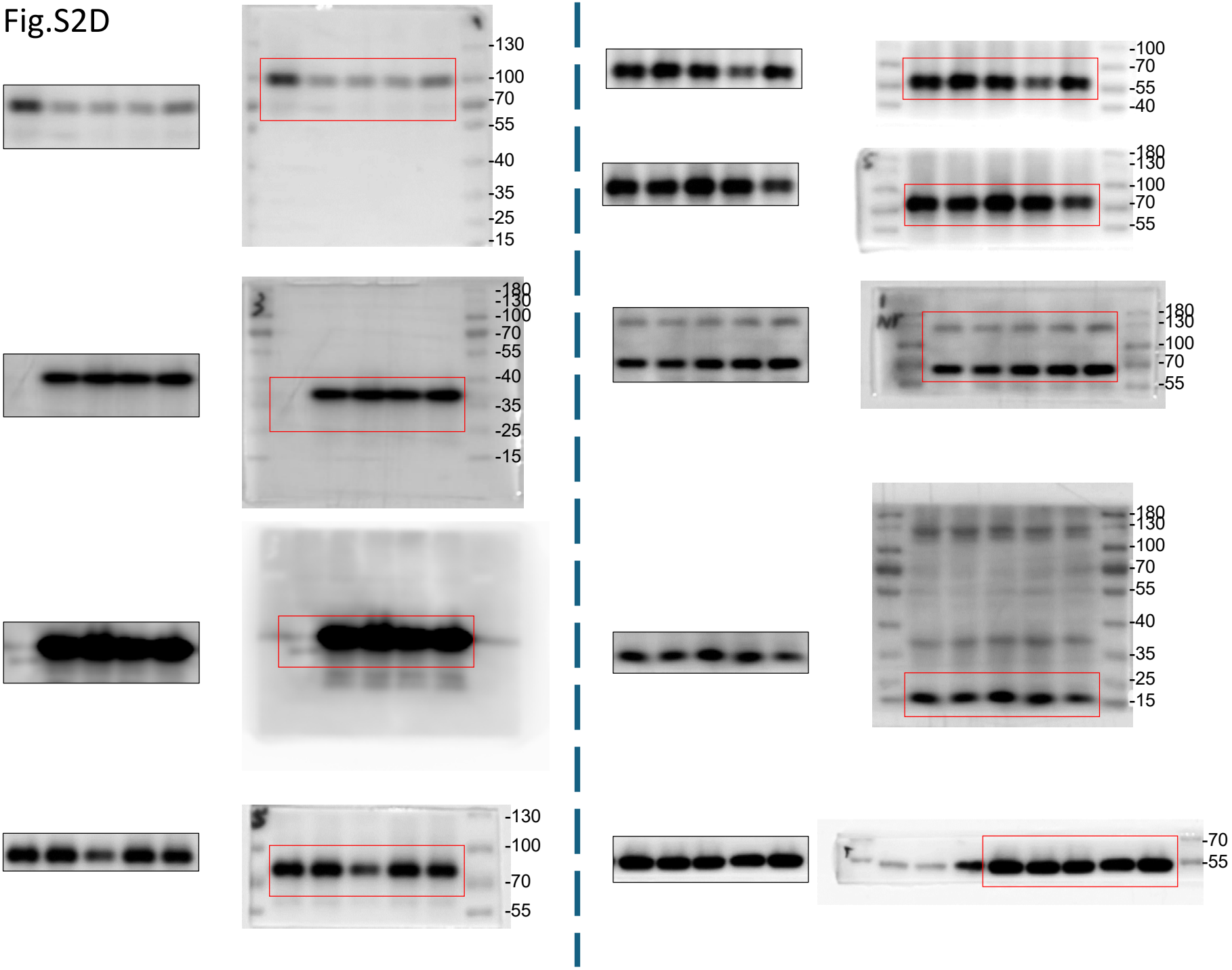

Fig.S2F

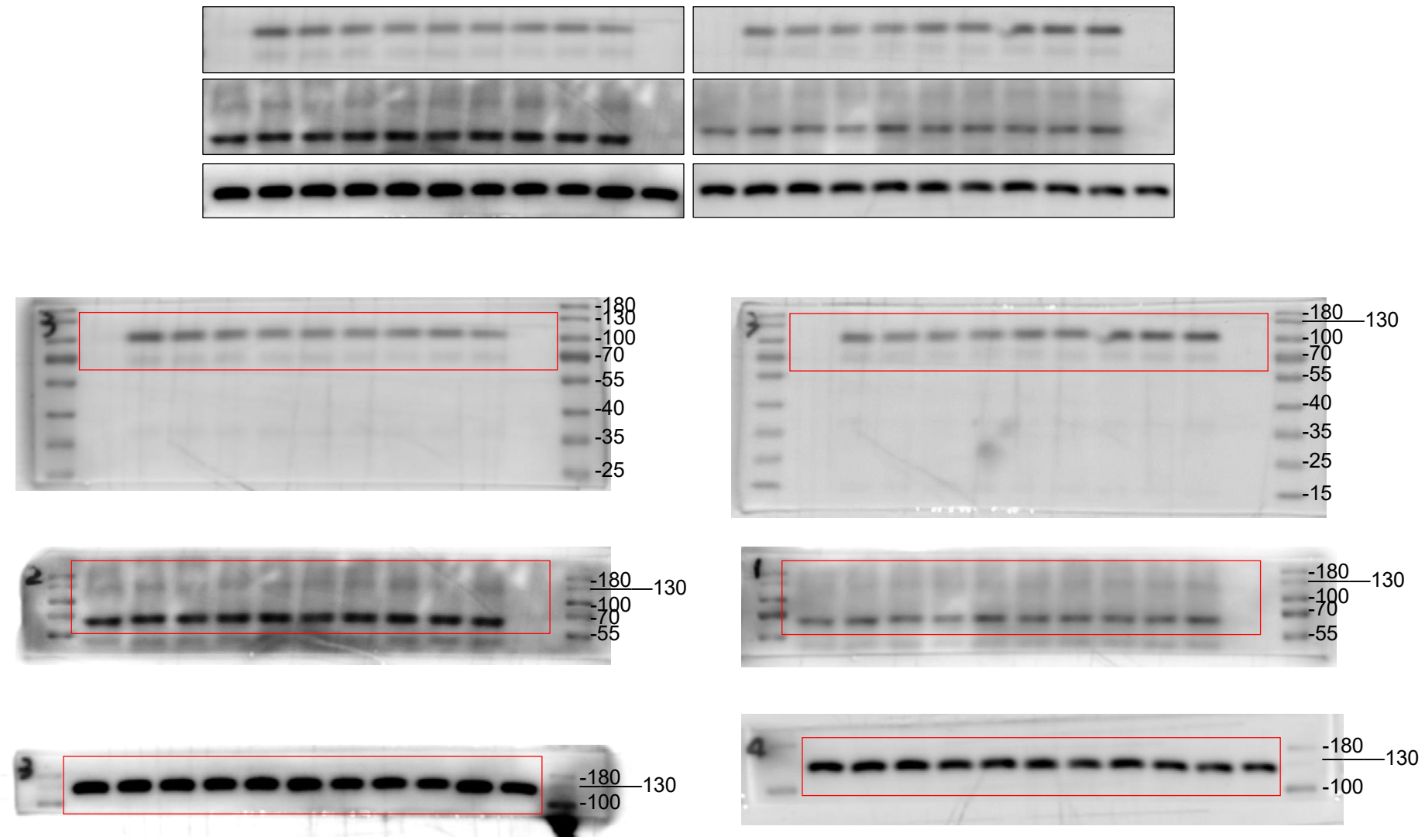

Fig.S2G

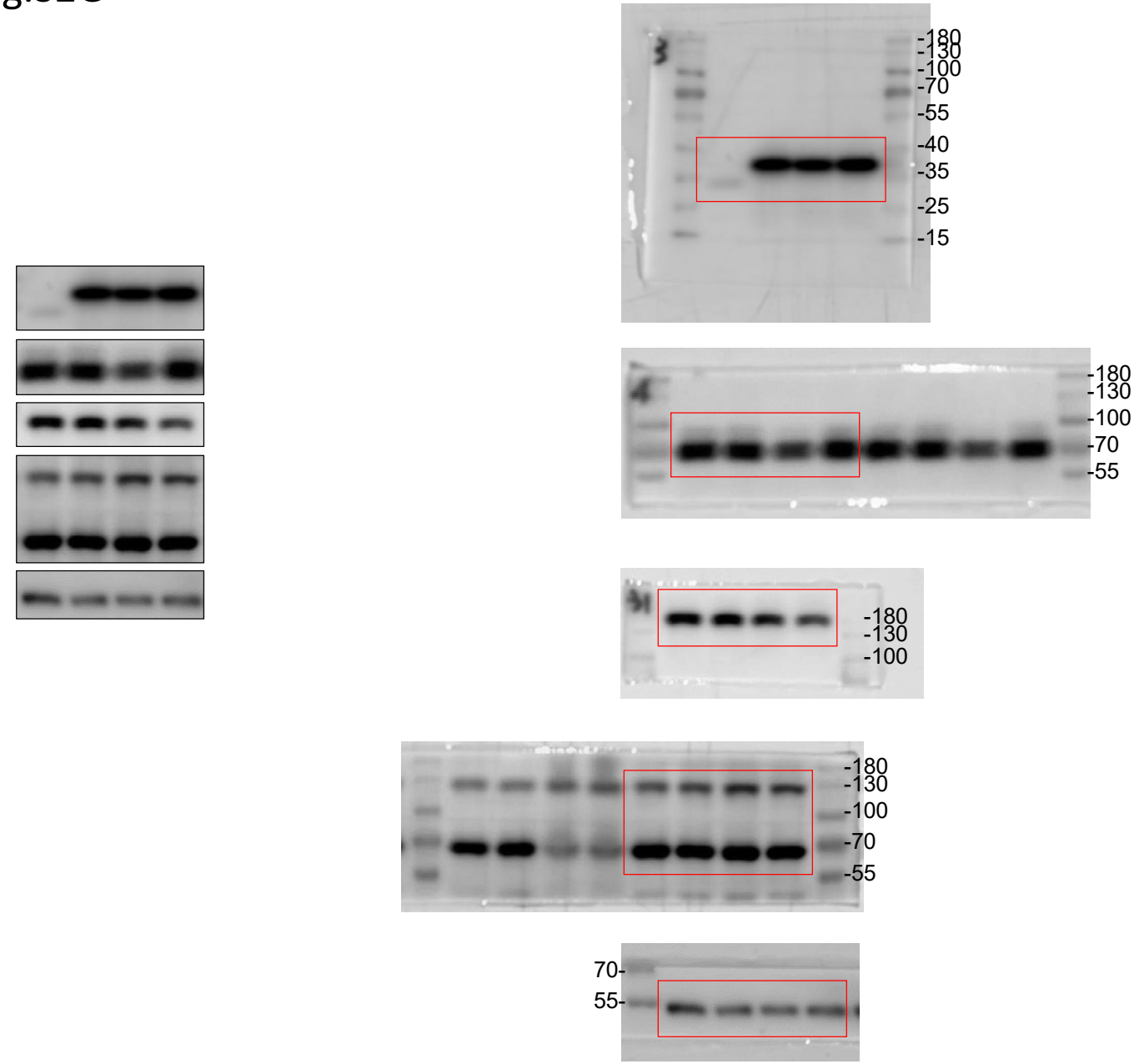

-180
-130
-100
-70
-55
-40
-35
-25
-15
-180
-130
-100
-70
-55
-180
-130
-100
-180
-130
-100
-70
-55
70-
55-

Fig.S2H

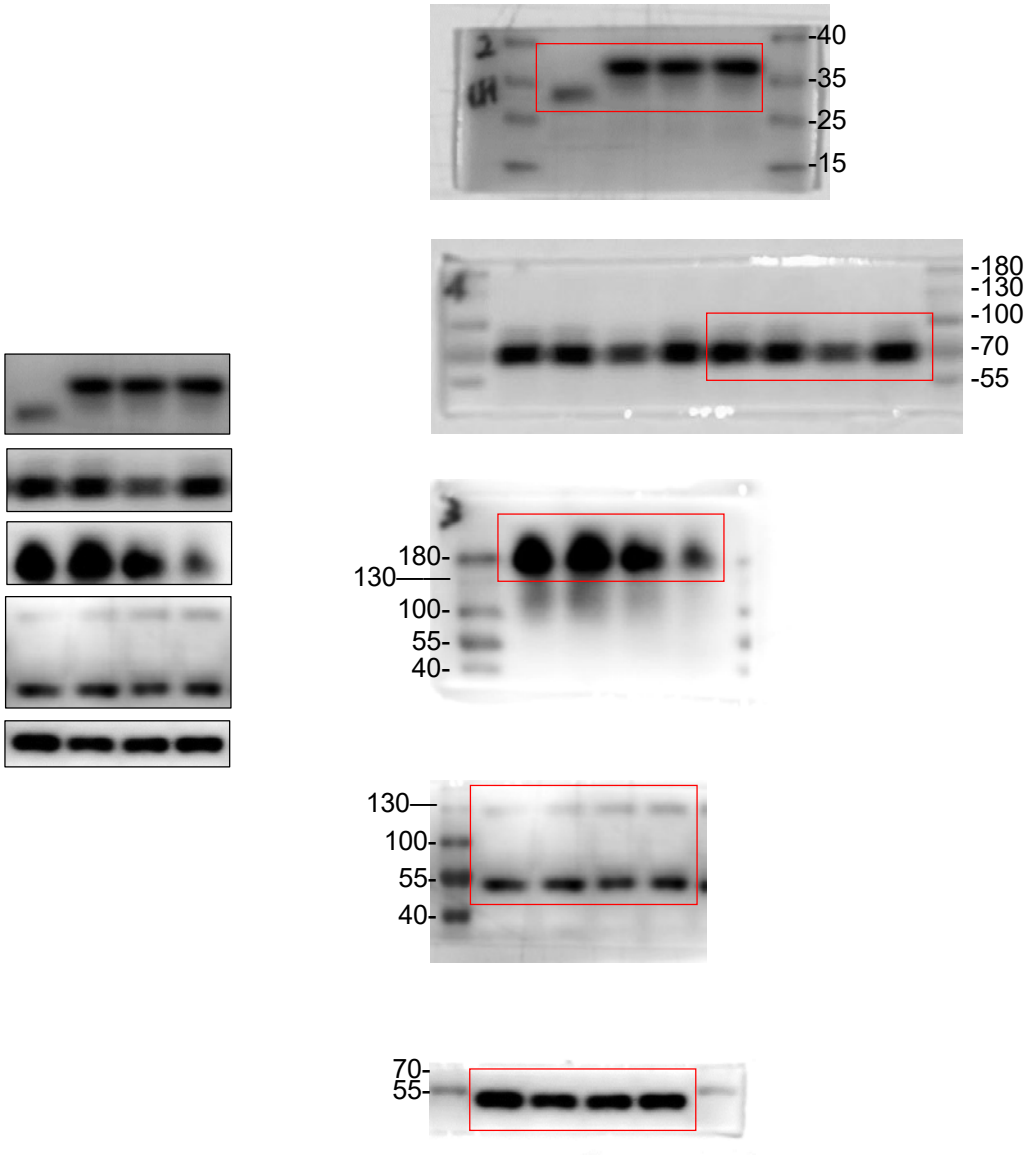

Fig.S4I

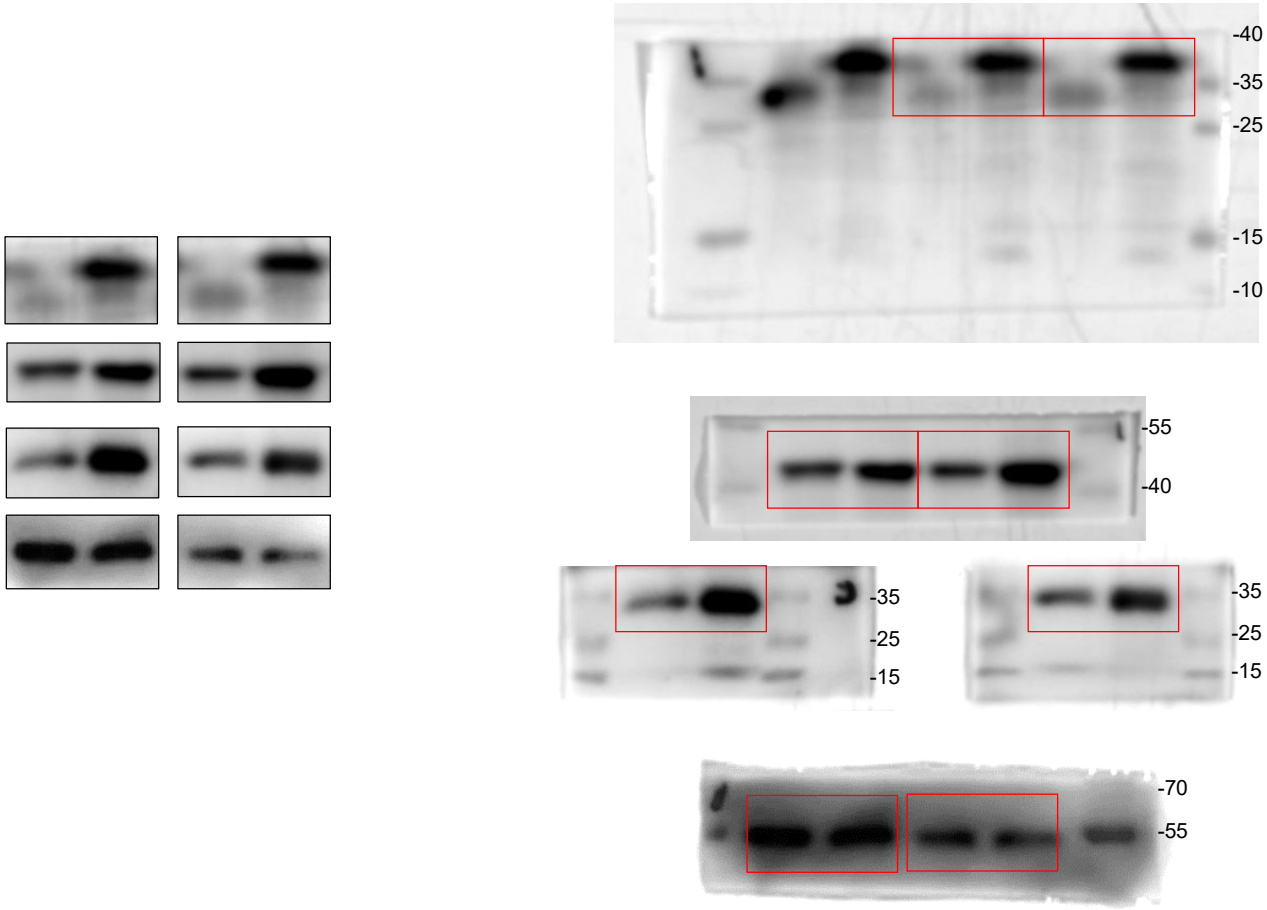

Supplement: Supplementary file 9 [file mmc9.pdf]
